# Room-Temperature Phosphorescence and Cellular Phototoxicity Activated by Triplet Dynamics in Aggregates of Push–Pull Phenothiazine-Based Isomers

**DOI:** 10.1021/acs.jpcb.2c07717

**Published:** 2023-02-03

**Authors:** Tommaso Bianconi, Alessio Cesaretti, Pietro Mancini, Nicolò Montegiove, Eleonora Calzoni, Anupama Ekbote, Rajneesh Misra, Benedetta Carlotti

**Affiliations:** † Department of Chemistry, Biology and Biotechnology, University of Perugia, via Elce di Sotto 8, 06123 Perugia, Italy; ‡ Department of Chemistry, Indian Institute of Technology Indore, Indore 453552, India

## Abstract

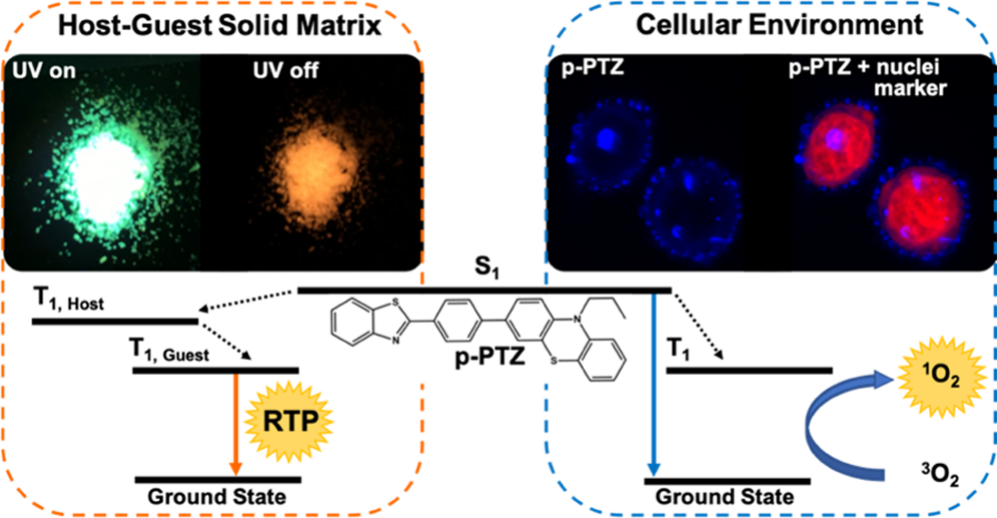

In this study, we report a comprehensive time-resolved spectroscopic investigation of the excited-state deactivation mechanism in three push–pull isomers characterized by a phenothiazine electron donor, a benzothiazole electron acceptor, and a phenyl π-bridge where the connection is realized at the relative *ortho*, *meta*, and *para* positions. Spin–orbit charge-transfer-induced intersystem crossing takes place with high yield in these all-organic donor–acceptor compounds, leading also to efficient production of singlet oxygen. Our spectroscopic results give clear evidence of room-temperature phosphorescence not only in solid-state host–guest matrices but also in highly biocompatible aggregates of these isomers produced in water dispersions, as rarely reported in the literature. Moreover, aggregates of the isomers could be internalized by lung cancer and melanoma cells and display bright luminescence without any dark cytotoxic effect. On the other hand, the isomers showed significant cellular phototoxicity against the tumor cells due to light-induced reactive oxygen species generation. Our findings strongly suggest that nanoaggregates of the investigated isomers are promising candidates for imaging-guided photodynamic therapy.

## Introduction

As a rising star, phototheranostics has attracted live interest for cancer treatment in the last years, allowing at the same time diagnostic imaging and *in situ* therapy triggered by light.^1,2^ Photodynamic therapy (PDT) is considered an excellent alternative to conventional therapeutic approaches for cancer under certain scenarios, due to its noninvasiveness, limited side effects, precise controllability, and good selectivity, together with excellent anticancer effects.^3−5^ In the presence of oxygen and light irradiation, an ideal photosensitizer for PDT undergoes efficient intersystem crossing (ISC) to a long-lived triplet state followed by the generation of reactive oxygen species (ROS). Significant ROS production has often been observed by integrating heavy-metal ions into the molecular structure of the photosensitizer to enhance the spin–orbit coupling and thus promote the ISC. However, the low biocompatibility of most heavy metals has made these organometallic compounds less competitive than metal-free systems. On the other hand, among the various existing diagnostic techniques, fluorescence imaging employing highly biocompatible all-organic fluorophores has captured much attention. Nevertheless, interference from tissue autofluorescence resulting in a low signal-to-noise ratio represents an important drawback. Recently, purely organic room-temperature phosphorescent (RTP) materials have emerged as promising probes for luminescence imaging and time-resolved luminescence imaging, as their long-wavelength emission and long emissive lifetimes allow us to minimize or eliminate the background interference from endogenous fluorescence.^6−11^ Two factors are absolutely essential to achieve significant organic RTP emission: (1) an effective ISC to produce the triplet excited state and (2) a rigid microenvironment, also capable of protecting the emitter from oxygen, to reduce the nonradiative triplet deactivation. A large number of strategies have emerged to boost spin–orbit coupling in organic chromophores, including the incorporation of heteroatom, carbonyl, and halogen units as well as folding-induced or charge transfer-induced ISC.^12−14^ In the latter, the processes of charge transfer and charge recombination induce a change in the angular momentum of the molecular orbitals to compensate for the change in the angular momentum of the electron spin during ISC, thus greatly enhancing ISC. This process is referred to as spin–orbit charge transfer-induced ISC (SOCT-ISC). Several methods have also been proposed to suppress the nonradiative relaxation of triplet excitons, such as crystal engineering, host–guest doping, metal–organic frameworks, self-assembly, and aggregation.^10,15,16^ In particular, the development of organic RTP materials with long-wavelength emission in the red region^17−20^ and ultralong lifetime emission^21−23^ is of great significance for bioimaging applications. Phenothiazine has been recently proposed in a few literature studies as a promising building block to design competitive organic RTP materials.^24−28^

**Chart 1:**
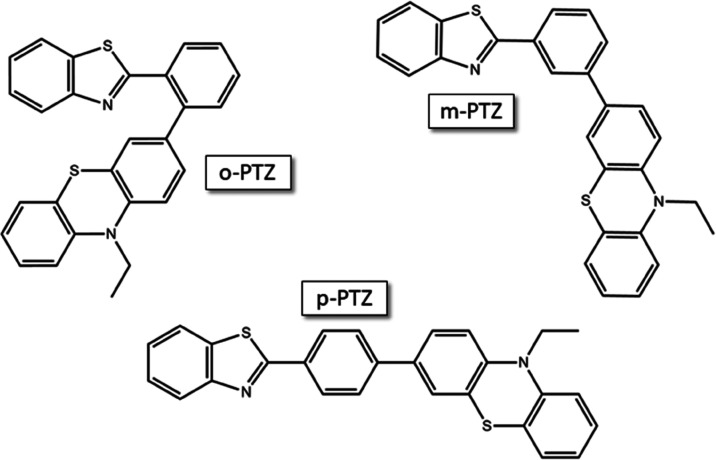
Molecular Structures of the Investigated Isomers

In this work, three donor–acceptor isomers (D−π–A) were taken into consideration. They are characterized by a phenothiazine electron-donor portion (D), a benzothiazole electron-acceptor unit (A), and a phenyl π-bridge, where the connection is realized at the relative *ortho*, *meta*, and *para* positions (***o*-PTZ**, ***m*-PTZ**, and ***p*-PTZ**, respectively, see Chart 1).^29^ The significant intramolecular charge transfer (ICT) in these isomers was used as a strategy to enhance the spin–orbit coupling and activate ISC. An advanced time-resolved spectroscopic investigation, with nanosecond and femtosecond time resolution, both in absorption and in emission, allowed us to gain a comprehensive mechanistic insight into their excited-state dynamics involving both ICT and ISC processes.^30^ Interestingly, these push–pull isomers proved to exhibit aggregation-induced emission (AIE).^29^ AIE is a photophysical phenomenon through which aggregate species produced in water dispersions or in the solid state are surprisingly found to show enhanced emission relative to their monomer in solution.^31,32^ With the present study, the AIE behavior and the significant ISC of the investigated isomers were exploited further to trigger their RTP in highly biocompatible aggregates obtained in water as well as in solid-state host–guest matrices.^11,33,34^ Moreover, their potential for biomedical applications was verified through experiments in a cellular environment involving lung cancer and melanoma cells. Not only the ability of aggregates of the isomers to be internalized by the cancer cells and display luminescence was tested, but also their effects of dark cytotoxicity and controlled cellular phototoxicity were thoroughly investigated and correlated to light-induced ROS generation.^35−40^

## Methods

### Chemicals

The synthetic procedures of ***o*-PTZ**, ***m*-PTZ**, and ***p*-PTZ** compounds have been already described in a previous paper.^29^ Spectral and photophysical characterizations were performed in several solvents of spectroscopic grade obtained from Sigma-Aldrich: pentane (Pent), *n*-hexane, cyclohexane, methylcyclohexane, 3-methylpentane, toluene (Tol), ethyl acetate (EtAc), acetone, acetonitrile, and dimethyl sulfoxide (DMSO). Spectral and photophysical measurements were also carried out in DMSO and water (W) mixtures. The host–guest doping materials were prepared by the melt-casting method. The host and guest materials mixed according to the desired molar ratio were placed in an oxygen-free sealed and clean reaction tube and then heated to melt. The temperature was determined by the melting point of the host compounds triphenylphosphine (TPP, 81.7 °C) or benzophenone (BPO, 48.5 °C).^30^ Dulbecco’s modified Eagle’s medium (DMEM), fetal bovine serum (FBS), Trypsin, and penicillin/streptomycin were purchased from Euroclone (Pero, Italy). Trypan Blue powder and 3-(4,5-dimethylthiazol-2-yl)-2,5-diphenyltetrazolium bromide (MTT) were purchased from Sigma-Aldrich (Saint Louis, MO) and Becton, Dickinson and Company (Franklin Lakes, NJ).

### Photophysical Measurements

The absorption spectra of the compounds’ solutions (≈1 × 10^–5^ M) were recorded using a Cary 4E (Varian) spectrophotometer. Fluorescence and excitation spectra were instead detected by an FS5 spectrofluorometer by Edinburgh Instruments with the appropriate instrumental response corrections. The fluorescence quantum yields (ϕ_F_, experimental error ±10%) of dilute solutions (1 × 10^–6^ M) were obtained by employing 9,10-diphenylanthracene (ϕ_F_ = 0.73 in air-equilibrated cyclohexane)^41^ as reference compound. Fluorescence lifetimes were measured using the time-correlated single-photon counting (TC-SPC) method through an Edinburgh Instrument FS5 spectrofluorometer, equipped with a LED source centered at 375 nm, with a 0.2 ns temporal resolution.

Singlet oxygen in air-equilibrated solution (≈1 × 10^–5^ M) was produced by sensitization experiments from the three isomers in cyclohexane and Tol. The ^1^O_2_ phosphorescence spectra were detected through a spectrofluorometer FS5 (Edinburgh Instrument) equipped with an InGaAs detector. Phenalenone (ϕ_Δ_ = 0.95 in cyclohexane and 0.99 in Tol) was used as the reference compound to obtain the singlet oxygen quantum yield (ϕ_Δ_, experimental error ±10%).^42^ The same spectrofluorometer was employed to acquire the phosphorescence spectra of the isomers by exciting the sample with a microsecond pulsed lamp with tunable excitation frequency (from 40 to 0.25 Hz) in the glass matrix constituted by methylcyclohexane (MeCH) and 3-methylpentane (3-MePent) 9:1 v/v ratio at 77 K as well as in host–guest solid-state matrices and in DMSO/W mixtures (1:99 v/v ratio).

Triplet properties were measured by laser flash photolysis (Edinburgh LP980) with pump pulses centered at 355 nm (third harmonic of a Quanta-Ray/Spectra Physics INDI Pulsed Nd:YAG Laser) with nanosecond-resolution (pulse width 7 ns and laser energy <1 mJ/pulse) coupled with a PMT for signal detection. A pulsed xenon lamp was then used to probe the absorption properties of the produced excited states. Energy transfer experiments in de-aerated conditions were exploited to determine the triplet absorption coefficient. Triplet–triplet absorption coefficients (ε_T_) were measured by energy transfer from 2,2′-dithienylketo (**DTK**, ε_T_ = 5000 M^–1^ cm^–1^ at 680 nm)^43,44^ to the isomers in acetonitrile. An actinometry approach was then used to measure the triplet quantum yields considering Benzophenone in acetonitrile (ϕ_T_ = 1.0 and ε_T_ = 6500 M^–1^ cm^–1^ at 520 nm)^45^ as a reference with known ϕ_T_ and ε_T_ values. The uncertainties were estimated to be about ±15% on ϕ_T_ and ±10% on the product ϕ_T_ × ε_T_. All measurements were performed by purging the sample with pure nitrogen.

The experimental setup for the femtosecond transient absorption and fluorescence up-conversion measurements has been widely described elsewhere.^46−49^ Particularly, the 800 nm radiation is amplified by the Ti:Sapphire laser system (Spectra Physics) and, successively, converted into the 400 nm excitation pulses (ca. 60 fs) by Apollo (2nd and 3rd harmonic generator). A small portion of the fundamental laser beam (800 nm light) enters the transient absorption spectrometer (Helios, Ultrafast Systems), passes through an optical delay line (time window of 3200 ps), and is finally focused onto a Sapphire crystal (2 mm thick) to generate a white-light continuum (450–800 nm), used as the probe. The temporal resolution is about 150 fs, and the spectral resolution is 1.5 nm. In the Up-Conversion setup (Halcyone, Ultrafast System), the 400 nm pulse excites the sample, whereas the fundamental laser beam acts as the “gate” light, after passing through a delay line. The fluorescence of the sample is collected and focused onto a BBO crystal together with the delayed gate beam to realize sum-frequency generation. A CCD detects the up-converted fluorescence. Movements of the crystal through a rotational stage allow for broadband detection of the emission at each delay and thus acquisition of the entire time-resolved fluorescence spectra. The time resolution is about 200 fs while the spectra resolution is 1.5 nm. Most measurements were carried out under the magic angle condition in a 2 mm cell having 0.5 < *A* < 1 at λ_pump_. The solution was stirred during the experiments to avoid photoproduct interferences. The absence of relevant photodegradation was checked by recording the absorption spectra before and after the time-resolved measurement, where no significant change was observed. The experimental 3D data matrixes were first analyzed by performing the Global Analysis by the Surface Xplorer PRO (Ultrafast Systems) software, and successively through the GloTarAn software to obtain the Evolution-Associated Spectra (EAS) considering a consecutive kinetic model.^50^

### Quantum Mechanical Calculations

Quantum mechanical calculations were performed using the Gaussian 16 package. B3LYP was chosen as the method to perform both the S_0_ geometry optimization. Every calculation was submitted setting 631-G+(d,p) as basis set.

### A549 and MEL-501 HeLa CCL-2 Cell Culture

MEL-501 human melanoma cells and A549 (CCL-185) human alveolar basal epithelial adenocarcinoma cells (ATCC, Manassas, VA) were cultured in a DMEM containing 10% (v/v) heat-inactivated FBS and penicillin (10 000 U/mL)/streptomycin (10 mg/mL). The cell concentration was monitored by Trypan blue dye staining, using an automated cell counter (Invitrogen Countess, Thermo Fisher Scientific, Waltham, MA).^51^

### MTT Assay

The MTT assay was used to study the effect of the isomers on cell proliferation.^52^ 2 × 10^3^ cells A549 or MEL-501 cells were seeded in Falcon 96-well clear flat-bottom microplates (Becton, Dickinson and Company, Franklin Lakes, NJ) with 200 μL of DMEM. After 24 h of incubation, the medium was replaced with 198 μL of fresh DMEM and 2 μL of different dilutions of the ***o*-PTZ** and ***m*-PTZ** isomers’ stock solution (1 mM in DMSO) were added into each well to reach concentrations ranging from 10 to 0.001 μM in quadruplicate. In the case of the ***p*-PTZ** compound, whose stock solution had a lower concentration (0.7 mM in DMSO) because of solubility issues, 3 μL of different dilutions were added to 197 μL of fresh DMEM into each well to attain the same concentration explored for the other isomers. A quadruplet was kept as control (200 μL of DMEM) and another quadruplet was used to take into account the contribution of DMSO (vehicle control 198 μL of DMEM + 2 μL of DMSO or 197 μL of DMEM + 3 μL of DMSO). After 72 h of incubation in a humidified atmosphere with 5% CO_2_ at 37 °C, 20 μL of a 5 mg/mL MTT dye solution was added to each well to reach a final concentration of 0.5 mg/mL. The cells were then incubated in a humidified atmosphere with 5% CO_2_ at 37 °C for 3 h to allow the formation of formazan crystals, which were subsequently dissolved in 150 μL of DMSO at 37 °C for at least 30 min. After a brief mechanical shaking of the microplates, the optical density at 570 nm was determined using a microplate reader (Beckman Coulter DTX880, Beckman Coulter, Inc., Brea, CA). Cell viability was expressed as the optical density percentage in treated cells compared with vehicle controls, assuming the absorbance of controls was 100% (absorbance of treated wells/absorbance of control wells × 100). All measurements were performed in two independent experiments.

### Fluorescence Microscopy

1500 A549 and MEL-501 cells were seeded on round glass coverslips previously sterilized by 30 s of immersion in 70% ethanol, rinsed with sterile phosphate buffer saline (PBS), and placed in a Falcon 24-well clear flat-bottom multiwell cell culture plates (Becton, Dickinson and Company, Franklin Lakes, NJ). The cells were then incubated for 45 min in a humidified atmosphere with 5% CO_2_ at 37 °C, and subsequently, 500 μL of DMEM was gently added to each well. After that, cells were incubated for 24 h under canonical culture conditions (humidified atmosphere with 5% CO_2_ at 37 °C). 200 μL of an isomer compound solution diluted in DMEM at the final concentration of 10 μM and 200 μL of 10 μM C5, a nucleus fluorescent marker,^53^ were then administered to the cells, incubated for 2 h in a humidified atmosphere with 5% CO_2_ at 37 °C. Cells on round glass coverslips were then rinsed twice with PBS and fixed in 4% paraformaldehyde for 20 min at room temperature. After washing with PBS, samples were mounted with Mowiol 4-88 (Sigma-Aldrich, Saint Louis, MO). Image acquisition was performed using a fluorescence microscope (Eclipse TE2000-S, Nikon, Tokyo, Japan) equipped with the F-View II FireWire camera (Olympus Soft Imaging Solutions GmbH, Münster, Germany) and through the use of Cell^F^ Imaging Software (Olympus Soft Imaging Solutions GmbH, Münster, Germany). The DAPI filter (λ_exc_ = 385–400 nm; λ_em_ = 450–465 nm) was used to look at the fluorescence of the three isomers, while the TRITC filter (λ_exc_ = 545–565 nm; λ_em_ = 580–620 nm) was employed to observe the red luminescence of the nuclei marker C5.

### Phototoxicity Assay

MEL-501 and A549 cells were seeded in Falcon 96-well clear flat-bottom microplates (Becton, Dickinson and Company, Franklin Lakes, NJ) (2 × 10^3^ cells/well), in 200 μL of culture medium (DMEM). The following day, the medium was substituted with a fresh medium containing different concentrations of the isomer compounds. A quadruplet was kept as control (200 μL of DMEM) and another quadruplet was used to take into account the contribution of DMSO (vehicle control 198 μL of DMEM + 2 μL of DMSO or 197 μL of DMEM + 3 μL of DMSO). The cells were photoexposed for 25 or 50 min in a LED chamber (λ_exc_ = 390–400 nm) producing a power of about 1.7 mW/cm^2^. Following 72 h from the exposure, the MTT assay was performed to assess cell viability. Cell viability was expressed as the optical density percentage in treated cells compared with that of vehicle controls undergoing the same photoexposition.

To evaluate the phototoxicity as a function of exposure time, MEL-501 and A549 cells were seeded Falcon 96-well clear flat-bottom microplates (Becton, Dickinson and Company, Franklin Lakes, NJ), in 200 μL of culture medium (DMEM). The following day, the medium was substituted with a fresh medium containing 10 μM of the isomer compounds. A quadruplet was kept as control (200 μL of DMEM) and another quadruplet was used to take into account the contribution of DMSO (vehicle control 198 μL of DMEM + 2 μL of DMSO or 197 μL of DMEM + 3 μL of DMSO). The cells were photoexposed for 6′ 15″, 12′ 30″; 25′, and 50′ in the same LED chamber used before. Following 72 h from the exposure, the MTT assay was again carried out to determine the phototoxic effect at different exposure times. Cell viability was expressed as the optical density percentage in treated cells compared with that of vehicle controls undergoing the same photoexposition. All measurements were performed in quadruplicate in two independent experiments.

### Evaluation of Intracellular ROS Production

Intracellular ROS levels were measured using the H_2_DCFHDA method.^54^ DCFH-DA is a nonfluorescent probe that readily diffuses through the cell membrane and is hydrolyzed by the activity of intracellular esterases to form DCFH, which is then rapidly oxidized to form highly fluorescent DCF in the presence of ROS. Therefore, the intensity of the fluorescence signal is proportional to ROS production. MEL-501 and A549 cells were seeded in Corning 96-well black round bottom polystyrene microplates (Corning Incorporated, Corning, NY) (10 × 10^3^ cells/well), in 200 μL of culture medium (DMEM). The following day, the medium was substituted with a fresh medium containing different concentrations of ***o*-PTZ** and ***p*-PTZ** isomer compounds. A quadruplet was kept as vehicle control to take into account the effect of DMSO (198 μL of DMEM + 2 μL of DMSO or 197 μL of DMEM + 3 μL of DMSO) and another quadruplet was used to monitor the possible contribution of the isomer compounds’ autofluorescence. The cells were photoexposed for 25′ or 50′ in the LED chamber corresponding to irradiation energies of 2.55 and 5.04 J/cm^2^, respectively. After 30′, cells were washed with 100 μL of PBS and incubated with 10 μM H2DCFDA for 60 min, in a humidified atmosphere, at 37 °C, 5% CO_2_. Then, wells were washed with 100 μL of PBS and filled with 200 μL of PBS. The fluorescence intensity of the oxidized form of DCF was measured at excitation/emission wavelengths of 485/530 nm, respectively, using a microplate reader (Beckman Coulter DTX880, Beckman Coulter, Inc., Brea, CA). Data (expressed as the percentage of DCF fluorescence intensity with respect to vehicle control) were normalized to cell viability evaluated by the MTT assay performed on another Falcon 96-well clear flat-bottom microplates (Becton, Dickinson and Company, Franklin Lakes, NJ) seeded with cells simultaneously treated and photoexposed under the same experimental conditions. All measurements were performed in quadruplicate in two independent experiments.

## Results and Discussion

### Fluorescence and Triplet Properties

The fluorescence properties of the three donor–acceptor isomers in solution were investigated in many solvents of different polarities. A significant positive solvatochromism of the emission was observed, as the fluorescence spectrum was found to lose its vibrational structure and largely red-shift upon increasing the solvent polarity (Figures S1–S3).^55,56^ This resulted in a color change of the isomers’ fluorescence from blue in the least polar solvents, to green and yellow in the medium polarity solvents, to red in the most polar media (Figure S4). In some cases, a clear and peculiar dual emission was revealed for these push–pull systems.^57^ This spectral behavior clearly suggests the presence of two emitting states of different nature, whose relative energy is highly sensitive to the solvent. The fluorescence quantum yields and lifetimes were also investigated in many different solvents (Table S1). The *ortho* and *meta* isomers were generally found to be poorly fluorescent (quantum yields lower than 19 and 13%, respectively), while the *para* isomer is instead highly fluorescent in all of the investigated solvents (quantum yields higher than 21%), as detailed in Table S1. Interestingly, the fluorescence efficiency clearly shows a trend with the solvent polarity: it generally increases when passing from Pentane (Pent) to Toluene (Tol), (e.g., ϕ_F_ from 0.037 to 0.19 for ***o*-PTZ**) and subsequently decreases from Tol to dimethyl sulfoxide (DMSO), (see Tables S1 and 1). The fluorescence quenching observed in the most polar solvents is by 1 order of magnitude for ***o*-PTZ** (e.g., ϕ_F_ is 0.19 in Tol and 0.028 in DMSO), by 2 orders of magnitude for ***m*-PTZ** (e.g., ϕ_F_ is 0.13 in Tol and 0.0023 in DMSO) and just by ∼4 times for ***p*-PTZ** (e.g., ϕ_F_ is 0.81 in Tol and 0.22 in DMSO). A similar trend with the solvent polarity was observed for the fluorescence kinetics (Figure S5) and lifetimes (Table S1). The lifetimes, generally of a few nanoseconds, were found to increase when going from Pent to Ethyl Acetate (EtAc) and then decrease from EtAc to DMSO. Altogether, the results of the fluorescence quantum yields and lifetimes suggest that for these isomers there may be two deactivation pathways competitive with the fluorescence, one operative in nonpolar solvents and the other operative in highly polar media, respectively. The deactivation process leading to the fluorescence quenching observed in the most polar solvents and to the positive fluorosolvatochromism for these push–pull compounds may be photoinduced intramolecular charge transfer (ICT). This would also be in agreement with the results of the quantum chemical simulations about the frontier molecular orbitals (Figure S10). Indeed, the HOMO is mainly confined on the donor phenothiazine unit extending toward the phenyl π-linker, while the LUMO is localized on the benzothiazole acceptor moiety.

**Table 1:** Fluorescence, Triplet, and Singlet Oxygen Quantum Yields for the Isomers in Solvents of Different Polarities^a^

	***o*-PTZ**			***m*-PTZ**			***p*-PTZ**		
solvent	ϕ_F_	ϕ_T_	ϕ_Δ_	ϕ_F_	ϕ_T_	ϕ_Δ_	ϕ_F_	ϕ_T_	ϕ_Δ_
Pent	0.037	1.05	0.31^b^	0.15	1.03	0.27^b^	0.42	0.36	0.12^b^
Tol	0.19	0.57	0.39	0.13	0.88	0.39	0.81	0.10	0.10
DMSO	0.028	0.10		0.0023	0.013		0.22	0.042	

a Uncertainties are estimated to be about ±10% on ϕ_F_, ±15% on ϕ_T_, and ±10% on ϕ_Δ_.

b In cyclohexane.

**Figure 1:**
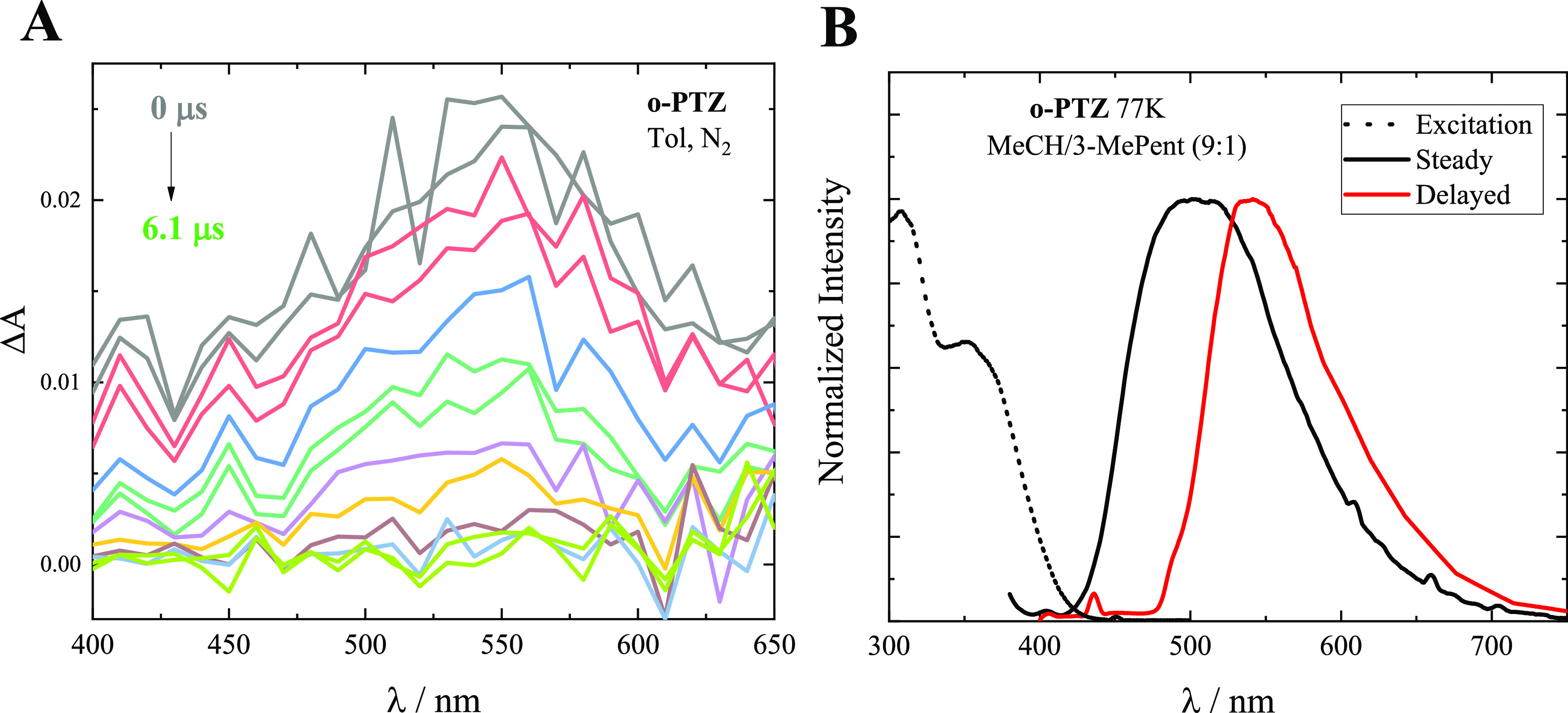
(A) Triplet spectra obtained by nanosecond laser flash photolysis for ***o*-PTZ** in nitrogen-purged toluene (Tol). (B) Steady (fluorescence) and delayed (phosphorescence) emission as well as excitation spectra of ***o*-PTZ** in methylcyclohexane/3-methylpentane mixture (MeCH/3-MePent 9:1) at 77 K.

To investigate the nature of the deactivation process competitive to the emission in nonpolar media, following the hypothesis that this may be the intersystem crossing (ISC), nanosecond laser flash photolysis measurements were carried out. Significant excited-state absorption signals were revealed for the three isomers in Tol solutions, with the transient spectra being peaked around 550 nm for ***o*-PTZ**, 560 nm for ***m*-PTZ**, and 500 nm for ***p*-PTZ**, respectively (see Figures 1A and S6). This transient species showed a lifetime of tens of nanoseconds in air-equilibrated Tol and a few/tens of microseconds in nitrogen-purged Tol, as detailed in Table S2. The significant oxygen effect observed on its lifetimes together with its ability to be sensitized by high-energy triplet donors (such as 2,2′-dithienylketo, DTK, in acetonitrile) allowed this transient species to be assigned to the lowest excited triplet state of the isomers. The quantitative sensitization experiments also allowed obtaining the triplet extinction coefficients for the three isomers (ε_T_ in Table S2), found to be somehow higher for the *meta* and *para* isomers compared to the *ortho* derivative. Relative actinometry measurements were performed in three crucial solvents of different polarities (Pent, Tol, and DMSO) to obtain the ϕ_T_ × ε_T_ product (see Table S2), and consequently the triplet yield values ϕ_T_ for the isomers (see Tables S2 and 1). The triplet production in Pent was found to be quantitative (ϕ_T_ ca. 1) for ***o*-PTZ** and ***m*-PTZ**, while being lower for the case of ***p*-PTZ** (ϕ_T_ =0.36). This behavior is in line with the much higher fluorescence yields obtained for ***p*-PTZ** relative to the other two isomers. The triplet yield showed a decrease with the solvent polarity for all three compounds. A more significant decrease in the triplet efficiency was observed when passing from Pent to DMSO for ***m*-PTZ** (by 2 orders of magnitude) relative to ***o*-PTZ** and ***p*-PTZ** (by 1 order of magnitude).

Given the large triplet production observed for the isomers in a nonpolar environment, phosphorescence measurements were carried out in a low polarity solvent mixture (methylcyclohexane/3-methylpentane 9:1) able to form a transparent solid matrix at 77 K. A distinct phosphorescence band was detected in the delayed spectrum as a clearly red-shifted band relative to the corresponding steady fluorescence emission (Figures 1B and S7). The phosphorescence, which peaked between 520 and 565 nm depending on the isomer (Table S3), showed a slow decay in the hundreds of millisecond time scale (Figure S8).

In nonpolar solvents, where ISC efficiently takes place, a significant production of singlet oxygen was also observed, through the detection of its infrared phosphorescence at 1270 nm (Figure S9). The singlet oxygen quantum yield in Tol was measured to be ca. 40% for ***o*-PTZ** and ***m*-PTZ** and ca. 10% for ***p*-PTZ**, in line with the results obtained for the triplet quantum yields (Table 1). Hence, on the one hand, the remarkable fluorescence properties of these isomers make them suitable as markers for imaging applications, on the other hand, their ability to efficiently give ISC and produce singlet oxygen upon irradiation put them forward for phototherapeutic applications.

### Ultrafast Excited-State Dynamics

To understand the behavior of the isomers upon photoexcitation in terms of preferential deactivation pathways, the excited-state dynamics of the isomers in both a nonpolar (Tol) and a polar (DMSO) solvent were comprehensively investigated by means of ultrafast spectroscopic experiments, in emission through femtosecond broadband fluorescence up-conversion as well as in absorption through femtosecond transient absorption.

**Figure 2:**
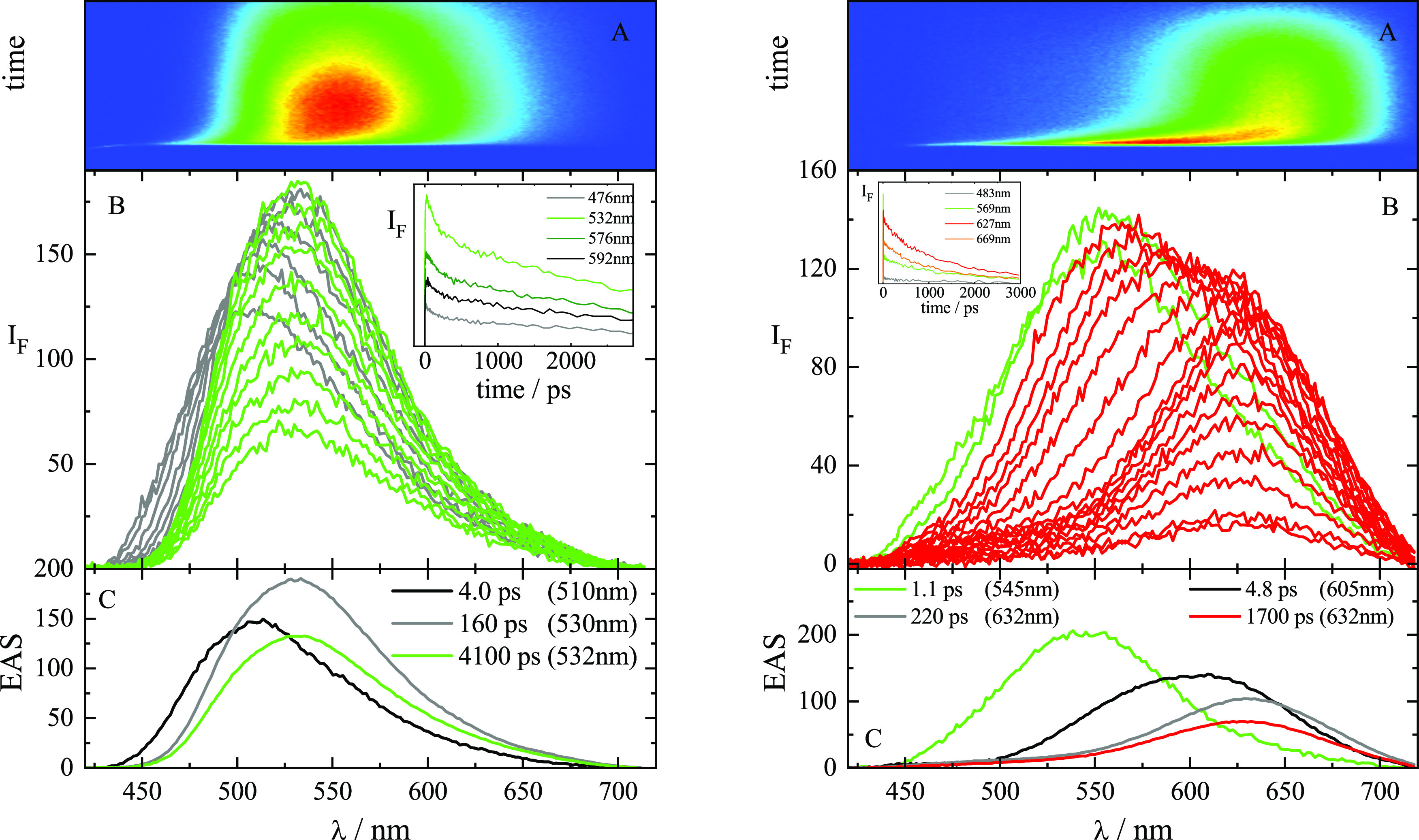
Femtosecond broadband fluorescence up-conversion spectroscopy of ***p*-PTZ** in Tol (left) and DMSO (right).

The fluorescence up-conversion results are reported in Figure 2 for the representative case of ***p*-PTZ** and in Figures S15 and S16 for the other two isomers. The time-resolved emission spectra in Tol (Figure 2B, left) highlight the presence of one emitting species whose fluorescence peaks at ca. 540 nm (in green) and undergoes just a small redshift at early time delays likely due to solvent relaxation. The global fitting of these data revealed three exponential components (Figure 2C, left and Table 2) associated with solvent and structural relaxation (black and gray, respectively), and to the lowest excited singlet state S_1_ (green).

**Table 2:**
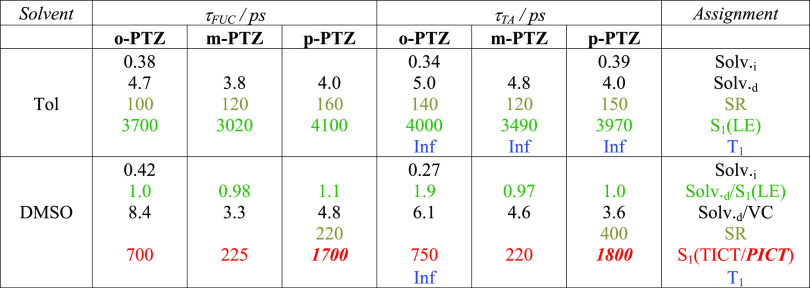
Results of Global Analysis of the Femtosecond Broadband Fluorescence Up-Conversion (FUC) and Transient Absorption (TA) Data in Toluene (Tol) and Dimethyl Sulfoxide (DMSO)^a^

a Experimental uncertainty on the lifetime values is ca. ±10% for the decay of the relaxed excited states while being larger (up to ca. ±30%) for the faster relaxation components.

The fluorescence up-conversion spectra for ***p*-PTZ** in DMSO (Figure 2B, right) show instead a large redshift of the emission spectra with time. These results suggest that the locally excited S_1_ state produced upon light absorption (LE, in green) and emitting at ca. 530 nm converts into the intramolecular charge transfer excited state (ICT, in red) emitting at ca. 640 nm and stabilized in this polar solvent. This two-excited-state model gives a justification for the dual emission observed in some of the steady-state fluorescence spectra. The global fitting of the data obtained from the ultrafast experiments in DMSO revealed in all cases a component characterized by a lifetime of ca. 1 ps which was associated with the S_1_ (LE) state (see the green species in Figure 2C and Table 2). The components characterized by lifetimes of few and hundreds of picoseconds were assigned to diffusive solvation and structural relaxation processes (represented in black and gray, respectively). The longest component resulting from the fitting was associated with the fully relaxed S_1_ (ICT) state populated in DMSO (in red), characterized by lifetimes of ca. 700 ps for ***o*-PTZ**, ca. 220 ps for ***m*-PTZ**, and ca. 1700 ps for ***p*-PTZ**, respectively. The short lifetime of the ICT state for the *ortho* and *meta* isomers may be due to its twisted structure (TICT state) while the longer lifetime revealed for the *para* isomer may suggest a planar excited-state structure (PICT state) in this case,^55^ in line with the optimized geometries obtained through the theoretical calculations (Figure S11). These findings explain the significant decrease of the fluorescence quantum yields observed in polar solvents for ***o*-PTZ** and ***m*-PTZ**, in that twisted structures generally prefer nonradiative deactivation to the ground state by internal conversion. On the other hand, the significant fluorescence efficiency measured for ***p*-PTZ** even in highly polar solvents may be related to its planar excited-state structure.

**Figure 3:**
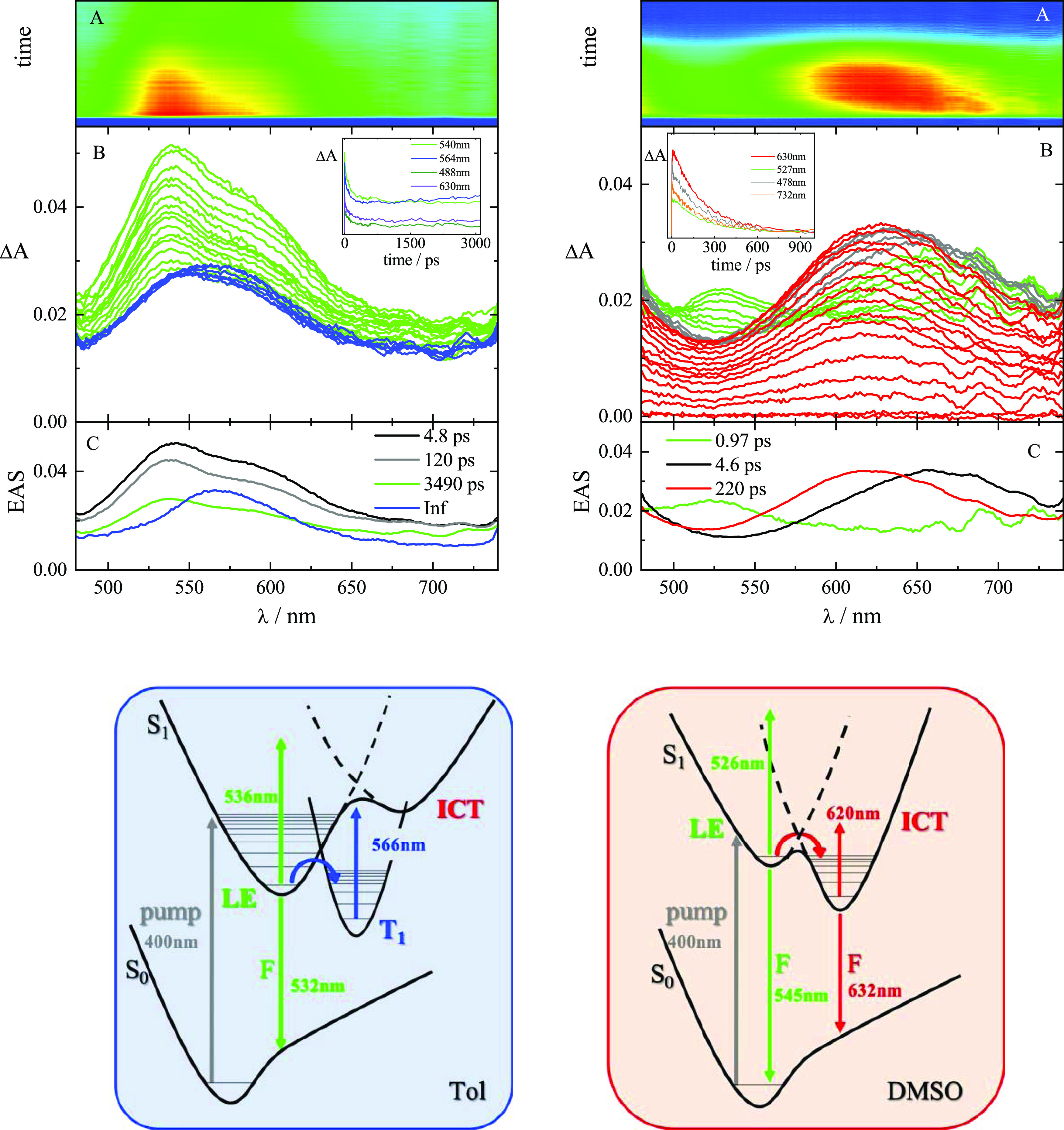
Top: Femtosecond transient absorption spectroscopy of ***m*-PTZ** in Tol (left) and DMSO (right). Bottom: Sketch of the excited-state dynamics in Tol (left) and DMSO (right).

The femtosecond transient absorption results are shown for the representative case of ***m*-PTZ** in Figure 3. Analogous results for the other two isomers are reported in Figures S13 and S14. The transient absorption spectra for ***m*-PTZ** in Tol (Figure 3B, left) show an initial structured excited-state absorption peaked at 530 nm (in green). With the decay of this species, a new broad excited-state absorption spectrum is formed centered at 560 nm (in blue) not decaying in the investigated time window of ca. 3000 ps. The global fitting of these data revealed the presence of four components (Figure 3C, left, and Table 2). The first two components of 4.8 and 120 ps (black and gray, respectively) were assigned to solvent and structural relaxation, respectively. The third component (green) characterized by a lifetime of 3.5 ns was associated with the relaxed fluorescent S_1_ state, consistently with the single-photon counting experiments (Table S1). The fourth component (blue), characterized by an infinite lifetime, was assigned to the lowest excited triplet state T_1_, in agreement with the nanosecond transient absorption experiments (Figure S6). In summary, the ultrafast absorption measurements in Tol disclosed the ISC dynamics for these isomers. These results are in step with the ultrafast emission experiments, being the T_1_ state not emissive at room temperature and thus only detectable with the transient absorption investigation.

Conversely, the transient absorption spectra recorded for ***m*-PTZ** in the polar solvent DMSO (Figure 3B, right) show that the excited-state absorption initially peaked at 530 nm (in green) quickly decays to form another transient species, which was not observed in Tol, characterized by a broad absorption spectrum centered around 630 nm (in red) and assigned to the ICT state. No sign of the population of the T_1_ state is revealed in this highly polar solvent, where ICT becomes the favored deactivation process.

In summary, the ultrafast spectroscopic experiments clearly disclosed the dynamics of the two processes competitive to the emission in these phenothiazine-based isomers: ISC in nonpolar media and ICT in highly polar solvents, respectively,^30^ as visually outlined in the sketches reported on the bottom of Figure 3. The trend of the dipole moment difference between the excited and the ground state (Δμ = |μ_ES_ – μ_GS_|) among the three isomers is Δμ(***m*-PTZ**) > Δμ(***p*-PTZ**) ≫ Δμ(***o*-PTZ**) (see Figure S12 and Table S4), as obtained from a quantitative analysis of their solvatochromism. These results suggest that the *meta* relative position of the donor and acceptor units is the one favoring the largest intramolecular charge transfer. On the other hand, the *ortho* position leads to a small Δμ due to the short distance between the donor and acceptor portions. However, the steric hindrance between the two chromophores implies a significantly twisted geometry (Figure S11) thus resulting in remarkable charge separation in the excited state. Therefore, ***m*-PTZ** and ***o*-PTZ** show a more significant ICT character in the excited states relative to ***p*-PTZ** (Table 2), but they do for two different reasons, namely, a favored charge flow at the *meta* position and an enhanced twisting of the molecular structure if the units are bridged at the *ortho* position, respectively. The stronger ICT character revealed for the *ortho* and particularly for the *meta* derivatives relative to the *para* is consistent with the more efficient ISC observed for the first two isomers, suggesting that spin–orbit charge transfer-induced ISC effectively takes place in these push–pull systems.^13,58,59^

### Aggregation and Room-Temperature Phosphorescence

**Figure 4:**
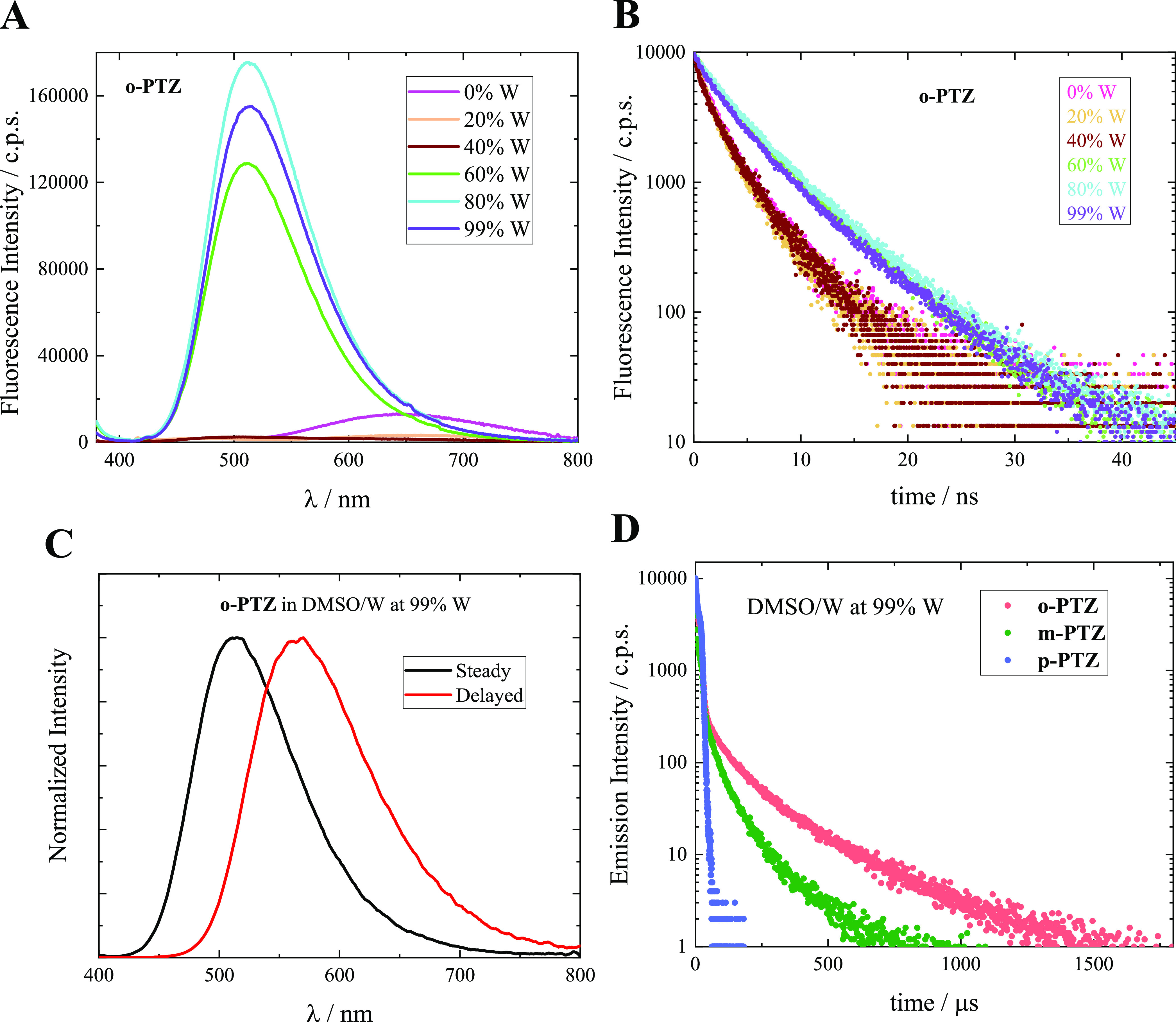
Fluorescence spectra (A) and kinetics (B) of the investigated isomers in DMSO/W mixtures containing different water amounts. (C) Prompt and delayed emission spectra for ***o*-PTZ** in DMSO/W mixture at 99% W. (D) Delayed emission kinetics for the isomers in DMSO/W mixture at 99% W.

The three phenothiazine-based isomers were also investigated in DMSO/water mixtures of different compositions. While the isomers show good solubility in DMSO, their solubility in water (W) is low, and when the amount of W in the mixture increases, this leads to aggregate formation.^29,30,60^ The size of the aggregates formed in the mixtures at 99% water amount was estimated to be tens of nanometers by means of dynamic light scattering measurements (Figure S17 and Table S5). A wider size distribution was obtained for the ***p*-PTZ** nanoaggregates in agreement with the findings of a previous work,^29^ where the presence of two different crystal structures was highlighted for this isomer in the aggregated state. The optical properties of these aggregates were studied by steady-state absorption and emission spectroscopy. Small changes were observed in the absorption spectra by varying the percentage of water in the mixture, while very significant variations were revealed in the emission spectra (Figures 4A and S18–S20). A clear aggregation-induced emission (AIE) behavior was observed for both ***o*-PTZ** and ***m*-PTZ**: weak emission was detected for the monomer species at 0, 20, and 40% W, while enhanced emission was revealed for the aggregates at 60, 80, and 99% W. The fluorescence quantum yields for the *ortho* and *meta* monomer species (at 0% W) were measured to be 1.8 and 0.4%, respectively, which were enhanced in the corresponding aggregate species (at 99% W) to 11 and 3.2%, respectively (Figure S21 and Table S6). In the case of the *para* isomer, a significant fluorescence efficiency was obtained for both the monomer at 0% W (ϕ_F_ = 22%) and the aggregate at 99% W (ϕ_F_ = 15%). It is noteworthy that clear AIE occurs for those molecules showing TICT excited states in polar environments, as the AIE may be activated by restriction of intramolecular rotations in these cases.

Fluorescence kinetics were acquired through nanosecond-resolved single-photon counting for the three isomers in DMSO/W mixtures of different compositions (Figures 4B and S22). Analysis of these kinetics clearly revealed a faster and generally monoexponential decay for the monomer species, as opposed to a slower and polyexponential decay for the aggregates (Table S6).^60^ Two groups of fluorescence kinetics were distinctly observed for ***o*-PTZ** (Figure 4B): the kinetics at 0, 20, and 40%W reflect the monomer faster decay, and the kinetics at 60, 80, and 99% reflect the aggregate slower decay. For ***m*-PTZ** and ***p*-PTZ** (Figure S22), a more gradual lengthening of the fluorescence lifetimes with increasing the water amount in the mixture was observed. The triexponential decays observed for the aggregate species of all of the isomers in water dispersions (Table S6) unambiguously reflect the heterogeneous nature of the produced nanosystems, as also reported in other literature studies.^60^ The excited-state dynamics of the aggregates formed by the three isomers in water dispersion was further investigated by means of femtosecond transient absorption and fluorescence up-conversion experiments (Figures S23 and S24). The signal-to-noise ratio of these data is quite poor due to scattering interference of the probe light by the big nanoaggregates. However, the ultrafast spectroscopic results seem to indicate that a transition occurs from a LE state characterized by a lifetime of few/tens picoseconds (in green) to an ICT excited state (in red) characterized by long lifetimes of several nanoseconds and likely a planar structure (Table S7). In water dispersion, which is a highly polar medium, the LE → ICT transition takes place for the aggregates (in 7–27 ps, see Table S7) but on longer time scales relative to the monomer species in DMSO solution (1–2 ps, see Table 2). The restriction of intramolecular rotations in the aggregates makes the ICT process slower and the relaxed ICT state a highly emissive PICT state characterized by a very long lifetime for all of the investigated isomers. Two-photon excited fluorescence experiments were carried out to investigate the possibility of inducing an excited-state population for the isomers also under biphotonic excitation. The two-photon absorption cross sections, only accessible with our experimental setup in the spectral region of the visible absorption tail, were not exceptionally high but still significant, particularly for the aggregate species (see Figure S25 and Table S8).

The most interesting result obtained from the investigation of the aggregates produced in water dispersions of the phenothiazine-based isomers was their ability to give room-temperature phosphorescence (RTP). A delayed emission band peaked around 560 nm distinct from the steady fluorescence was clearly detected for the ***o*-PTZ** and ***m*-PTZ** aggregates produced at 99% W (Figures 4C and S26). This band appears to be in the same spectral region where the phosphorescence was observed for the corresponding monomers at 77 K. Moreover, the delayed emission kinetics showed a long decay in the hundreds of microseconds time scale (Figure 4D). The best fitting of these kinetics was obtained by considering polyexponential functions for ***o*-PTZ** and ***m*-PTZ**, similarly to the prompt fluorescence decay kinetics, likely as a result of the microheterogeneous nature of the aggregates (Table S9). In the case of the ***p*-PTZ** aggregates formed at 99% W, the delayed emission decay occurs relatively faster (Figure 4D) and the best fitting was found to be monoexponential with a lifetime of ca. 5 μs (Table S9). This is probably due to the more planar structure of the *para* isomer, which favors π–π stacking interactions in the aggregate, leading to the quenching of the phosphorescence lifetime. Actually, during the photophysical investigation in solution, the ***p*-PTZ** isomer was found to be the one showing the largest fluorescence quantum yields among the three isomers in the series. For this reason, we believe that the signal detected also in the delayed/gated spectrum in water dispersion (Figure S26) is mainly due to the fluorescence emission of the ***p*-PTZ** aggregates, as it is not possible to disentangle the phosphorescence from the intense fluorescence emission for this sample. The observation of RTP in water dispersions of the isomer aggregates is a very interesting finding, as the long red-shifted emission could be detected in highly biocompatible systems. This has been reported in the literature only in a couple of recent scattered studies^17,19^ and thus constitutes a remarkable result from our investigation.

**Figure 5:**
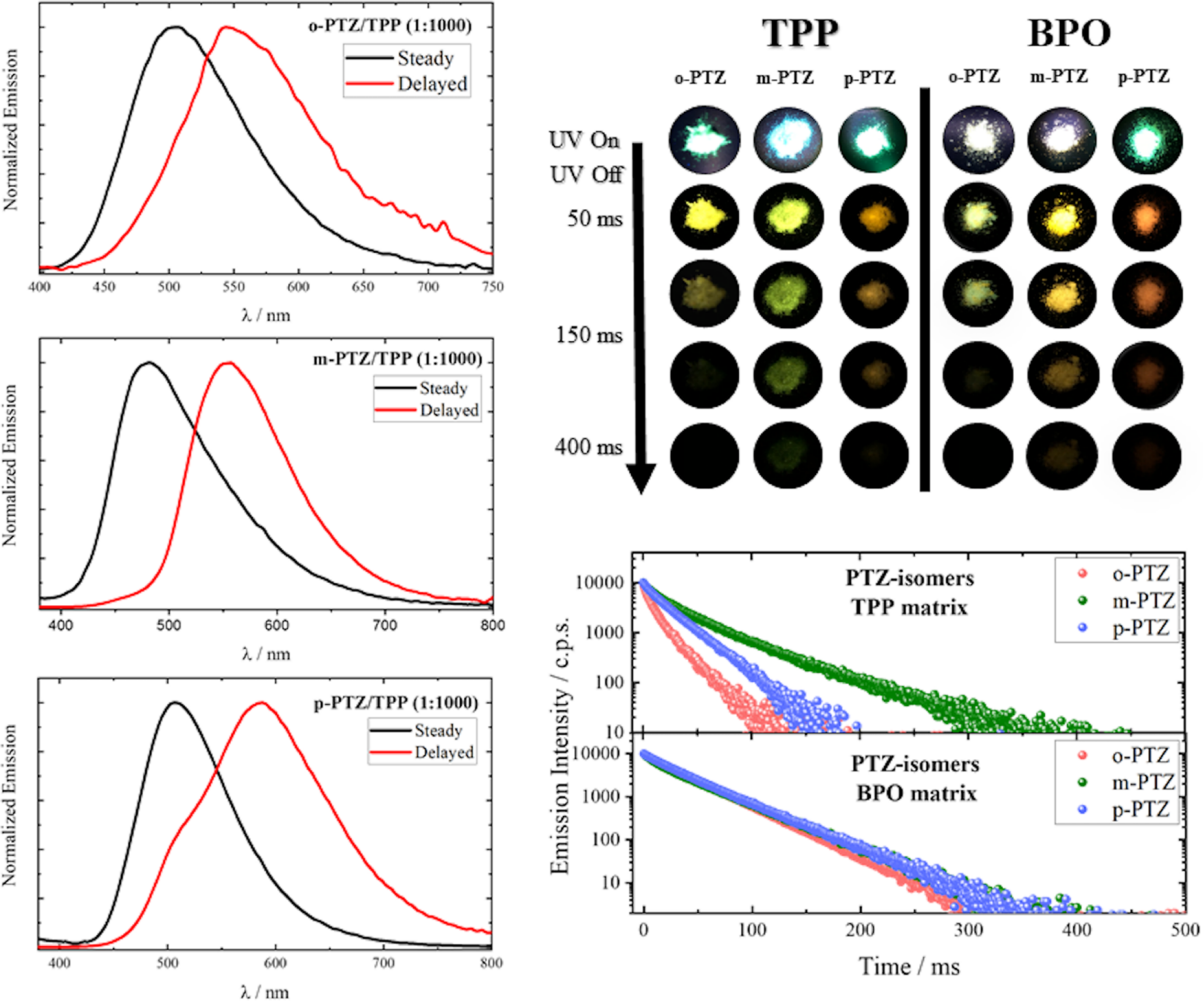
Steady-state and delayed emission spectra for the guest isomers in the TPP host solid matrices (left). Photographs of the solid-state host/guest powders at different delays after UV irradiation (top right). Delayed emission kinetics for the guest isomers in the TPP and BPO host solid matrices (bottom right).

The RTP ability of the phenothiazine-based isomers was also investigated in the solid state, by considering host–guest matrices employing triphenylphosphine (**TPP**) or benzophenone (**BPO**) as the host (Figures 5 and S27). When **TPP** was used as the host matrix, a phosphorescence band peaked between 540 and 590 nm was detected in the delayed spectra apparently distinct from the fluorescence at 480–500 nm recorded in the steady spectrum. The spectral behavior observed in the presence of **BPO** was found to be quite different, as it was more difficult to obtain the well-separated fluorescence and phosphorescence spectra. This may be due to the significant absorption of the excitation light (at 370 nm) by the **BPO** host,^20^ while the **TPP** absorption at the same wavelength is negligible.^30^ Consequently, the excited-state mechanism through which the S_1_ and T_1_ of the guest are populated implies preferential photoexcitation of the **BPO** followed by quantitative ISC to its high-energy triplet, leading to the observation of longer-lived and superimposed fluorescence and phosphorescence of the isomers. Indeed, the triplet state of the **BPO** lies well above both the singlet and the triplet excited states of the phenothiazine derivatives (Table S11 and Figures S28 and S29); Förster resonance energy transfer between S_1_ states as well as triplet–triplet energy transfer between host and guest may take place.^61^ On the other hand, **TPP** acts as a host providing a rigid and oxygen-poor environment where the phosphorescence of the guest is significant, with the guest S_1_ state being the only excited state populated upon light absorption. Figure 5 (at the bottom right) shows the recorded decay kinetics for the phosphorescence emission of the host–guest matrices. All of the kinetics are found to decay in the hundreds of millisecond time scale, revealing a persistent phosphorescence for the investigated samples (Table 3 and Table S10). It is noteworthy that, for the case of the **TPP** matrices, a longer emission lifetime was found for the case of the isomer guest with the singlet energy closer to the **TPP** triplet (***m*-PTZ**). The smaller energy gap between the ***m*-PTZ** singlet and the **TPP** triplet likely optimizes the role of the host in favoring the effective production of a long-lived triplet of the *meta* isomer. Photographs of the solid-state host/guest powders taken at different delays after UV irradiation are also reported in Figure 5 (top right). Interestingly, the matrices obtained considering the three isomers showed long-lasting emissions of different colors. While green and yellow emissions are observed for the ***o*-PTZ**- and ***m*-PTZ**-based powders, the phosphorescence detected for ***p*-PTZ** is orange/red, thus falling in the most interesting and most rarely spanned region of the visible spectrum with a view to imaging applications.

**Table 3:** Emission Properties of PTZ Isomers in TPP and BPO Matrices^a^

guest	host	λ_delayed_/nm	τ_delayed_/ms	host	λ_delayed_/nm	τ_delayed_/ms
***o*-PTZ**	**TPP**	544	8.55 (45%)	**BPO**	544	35.7
			27.1 (55%)			
***m*-PTZ**		554	20.7 (34%)		558	42.5
			61.6 (66%)			
***p*-PTZ**		587	21.1 (45%)		587	43.2
			36.1 (55%)			

a Experimental uncertainty on the lifetime values is ca. ±5%.

### Bioimaging and Cellular Phototoxicity

**Figure 6:**
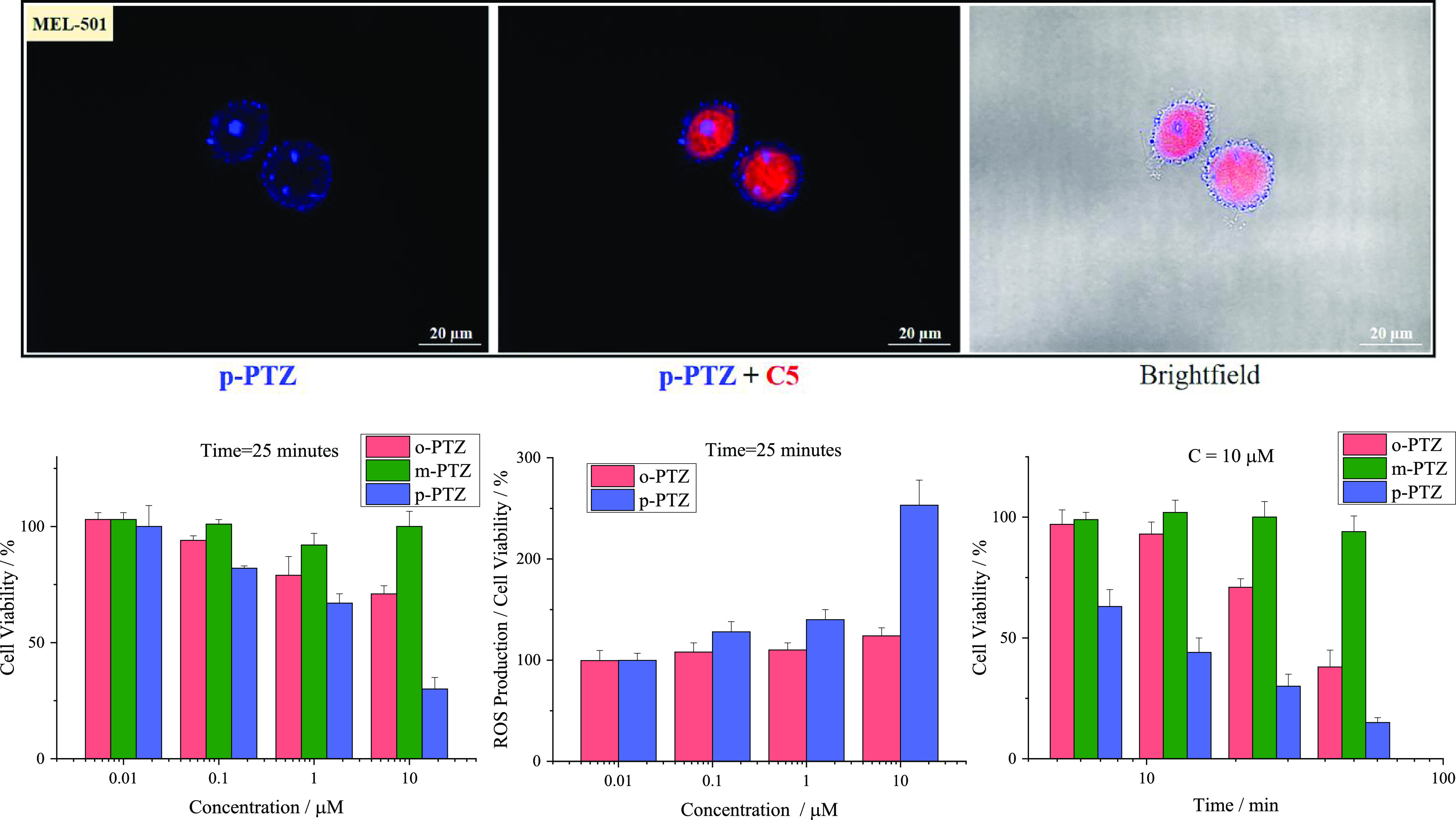
Top: Representative fluorescence microscopy images of fixed MEL-501 cells stained with 10 μM ***p*-PTZ** (blue, DAPI filter), 10 μM ***p*-PTZ** and the nuclei marker C5 (red, TRITC filter), and relative bright field merged images (image magnification: 60×). Bottom: Phototoxicity (left) and ROS production (central) of different concentrations of the isomers on MEL-501 cells with 25 min of irradiation time (corresponding to irradiation energy of 2.55 J/cm^2^, λ_exc_ = 390–400 nm); phototoxicity of 10 μM solutions of the isomers on MEL-501 cells at different irradiation times of 6, 12.5, 25, and 50 min (right). Cell viability is expressed as the mean of two independent experiments of four replicas each ±SD; 100% corresponds to control mean values.

With the aim of exploiting the great potential of the aggregates in biological applications, water dispersions of the three isomers at 99% W, with just a small amount of DMSO (<2%), were incubated with human alveolar basal epithelial adenocarcinoma cells (A549) and human melanoma cells (MEL-501). Subsequently, fluorescence images of the cells were taken through a wide-field fluorescence microscope (Figures 6 and S30), also staining the nuclei with a nuclear fluorescent marker, i.e., the C5 dye,^53^ for localization purposes. The fluorescence microscopy images showed that all three isomers were internalized by the cells and the dot emission of the aggregates was observed in the cytoplasmic and perinuclear cellular regions. Moreover, MTT assays revealed that the isomers, for any concentration in the range between 0.01 and 10 μM, exerted no cytotoxic effect on both the A549 and MEL-501 cells (Figures S31 and S33). The lack of dark cytotoxicity may be related to both the absence of localization for these dyes in a particular cellular compartment (e.g., nuclei or mitochondria) and their inability to interact with specific biological targets, such as nucleic acids.

Experiments to test the cellular phototoxicity of the investigated phenothiazine derivatives were also carried out, by varying either the concentration of the isomers or the time of irradiation (Figures 6, S32, and S34–S36 and Table S12). All three isomers were found to be phototoxic toward the tumor cells at a concentration of 10 μM and after 25 min of irradiation (or more). The *para* isomer resulted phototoxic even when used in more dilute solutions (1 μM) or by employing shorter irradiation times (6 min). This phototoxicity, effective also under milder experimental conditions, was also observed for the *ortho* compound, although to a lesser extent. The origin of the observed phototoxicity toward lung cancer cells (A549) and melanoma cells (MEL-501) was investigated through experiments aimed at searching for Reactive Oxygen Species (ROS) in the case of the most phototoxic ***p*-PTZ** and ***o*-PTZ** isomers. ROS were found to be generated in the investigated tumor cells well above the control levels upon 25 or 50 min of irradiation when solutions of the isomers at 10 μM concentration were considered (Figures 6, S32, and S35). These results obtained in the cellular environment are in good agreement with the spectroscopic measurements performed for the isomers in solution, where significant triplet and singlet oxygen production were revealed (Table 1). The particularly effective ROS production by ***p*-PTZ** in an *in vitro* cellular environment can be justified by the photophysics of this isomer which was found to be less sensitive to the solvent polarity compared to the *ortho* and particularly to the *meta* isomers (Table 1). Therefore, the triplet yields are expected to be maintained quite significant for ***p*-PTZ** even in highly polar aqueous solutions (and thus under biological conditions), while being drastically reduced for ***o*-PTZ**, and particularly for ***m*-PTZ**. The results obtained about the fluorescence imaging, cellular phototoxicity and light-induced ROS generation of the phenothiazine-based isomers in lung cancer and melanoma cells suggest that they may be excellent candidates as new photosensitizers for imaging-guided photodynamic cancer therapy (PDT). Moreover, the results of the two-photon excited fluorescence experiments suggest that excitation of the isomer-based aggregates may be performed in the biologically transparent window of the electromagnetic spectrum (in the red portion of the visible) and may be highly focused due to its nonlinearity. This may lead to improvement of both the penetration depth and the illumination selectivity, which are highly desirable for effective PDT.

## Conclusions

In summary, we report here a comparative study of three push–pull isomers where the electron-donor phenothiazine and the electron-acceptor benzothiazole units are connected at the *ortho*, *meta*, or *para* position of a phenyl π-bridge. An insightful investigation of the excited-state dynamics was performed through advanced time-resolved spectroscopies, with both nanosecond and femtosecond temporal resolution, revealing intersystem crossing and intramolecular charge transfer as the relevant deactivation pathways competitive with the fluorescence emission. Highly efficient spin–orbit charge transfer-induced intersystem crossing was found for these all-organic compounds in solution, with consequent remarkable singlet oxygen generation capability. Our time-resolved spectroscopic results show clear evidence of room-temperature phosphorescence not only in solid-state host–guest matrices but also in aggregates of the isomers produced in water dispersions, as rarely reported in the literature.^6,17,19^ A long phosphorescence emission persistent for hundreds of milliseconds was observed in the solid-state powders, characterized by a noticeable orange/red color in the case of the *para* isomer. Aggregates of all three isomers could be successfully internalized in lung cancer and melanoma cells, where their significant emission disclosed localization in the cytoplasmic and perinuclear regions while exerting no dark cytotoxic effect. Conversely, significant cellular phototoxicity toward the tumor cells was exhibited by the three isomers under light irradiation, clearly related to the reactive oxygen species photo-driven production. Our overall results strongly suggest that the highly biocompatible aggregates of the investigated isomers may be promising as new photosensitizers for imaging-guided photodynamic therapy.

## Supporting Information Available

Details about the experimental methods; additional spectral, photophysical, and ultrafast spectroscopic results; results from the theoretical calculations; and additional results obtained for the solid-state host–guest powders and in cellular environment (PDF)

## References

[ref1] Zhang Z.; Kang M.; Tan H.; Song N.; Li M.; Xiao P.; Yan D.; Zhang L.; Wang D.; Tang B. Z. The Fast-Growing Field of Photo-Driven Theranostics Based on Aggregation-Induced Emission. Chem. Soc. Rev. 2022, 51, 1983–2030. 10.1039/D1CS01138C. 35226010

[ref2] Wei X.; Zhang C.; He S.; Huang J.; Huang J.; Liew S. S.; Zeng Z.; Pu K. A Dual-Locked Activatable Phototheranostic Probe for Biomarker-Regulated Photodynamic and Photothermal Cancer Therapy. Angew. Chem., Int. Ed. 2022, 61, e202202966 10.1002/anie.202202966. 35396786

[ref3] Dolmans D. E. J. G. J.; Fukumura D.; Jain R. K. Photodynamic Therapy for Cancer. Nat. Rev. Cancer 2003, 3, 380–387. 10.1038/nrc1071. 12724736

[ref4] Li X.; Lovell J. F.; Yoon J.; Chen X. Clinical Development and Potential of Photothermal and Photodynamic Therapies for Cancer. Nat. Rev. Clin. Oncol. 2020, 17, 657–674. 10.1038/s41571-020-0410-2. 32699309

[ref5] Liu S.; Feng G.; Tang B. Z.; Liu B. Recent Advances of AIE Light-up Probes for Photodynamic Therapy. Chem. Sci. 2021, 12, 6488–6506. 10.1039/D1SC00045D. 34040725 PMC8132949

[ref6] Kenry; Chen C.; Liu B. Enhancing the Performance of Pure Organic Room-Temperature Phosphorescent Luminophores. Nat. Commun. 2019, 10, 2111 10.1038/s41467-019-10033-2. 31068598 PMC6506551

[ref7] Zhi J.; Zhou Q.; Shi H.; An Z.; Huang W. Organic Room Temperature Phosphorescence Materials for Biomedical Applications. Chem. – Asian J. 2020, 15, 947–957. 10.1002/asia.201901658. 32031734

[ref8] Zhao W.; He Z.; Tang B. Z. Room-Temperature Phosphorescence from Organic Aggregates. Nat. Rev. Mater. 2020, 5, 869–885. 10.1038/s41578-020-0223-z.

[ref9] Zhang T.; Ma X.; Wu H.; Zhu L.; Zhao Y.; Tian H. Molecular Engineering for Metal-Free Amorphous Materials with Room-Temperature Phosphorescence. Angew. Chem., Int. Ed. 2020, 59, 11206–11216. 10.1002/anie.201915433. 31876988

[ref10] Yan X.; Peng H.; Xiang Y.; Wang J.; Yu L.; Tao Y.; Li H.; Huang W.; Chen R. Recent Advances on Host–Guest Material Systems toward Organic Room Temperature Phosphorescence. Small 2022, 18, 2104073 10.1002/smll.202104073. 34725921

[ref11] Yang J.; Zhang Y.; Wu X.; Dai W.; Chen D.; Shi J.; Tong B.; Peng Q.; Xie H.; Cai Z.; Dong Y.; Zhang X. Rational Design of Pyrrole Derivatives with Aggregation-Induced Phosphorescence Characteristics for Time-Resolved and Two-Photon Luminescence Imaging. Nat. Commun. 2021, 12, 4883 10.1038/s41467-021-25174-6. 34385449 PMC8361132

[ref12] Hou Y.; Biskup T.; Rein S.; Wang Z.; Bussotti L.; Russo N.; Foggi P.; Zhao J.; Di Donato M.; Mazzone G.; Weber S. Spin–Orbit Charge Recombination Intersystem Crossing in Phenothiazine–Anthracene Compact Dyads: Effect of Molecular Conformation on Electronic Coupling, Electronic Transitions, and Electron Spin Polarizations of the Triplet States. J. Phys. Chem. C 2018, 122, 27850–27865. 10.1021/acs.jpcc.8b08965.

[ref13] Wang Z.; Ivanov M.; Gao Y.; Bussotti L.; Foggi P.; Zhang H.; Russo N.; Dick B.; Zhao J.; Di Donato M.; Mazzone G.; Luo L.; Fedin M. Spin–Orbit Charge-Transfer Intersystem Crossing (ISC) in Compact Electron Donor–Acceptor Dyads: ISC Mechanism and Application as Novel and Potent Photodynamic Therapy Reagents. Chem. – Eur. J. 2020, 26, 1091–1102. 10.1002/chem.201904306. 31743947

[ref14] Pan G.; Yang Z.; Liu H.; Wen Y.; Zhang X.; Shen Y.; Zhou C.; Zhang S.-T.; Yang B. Folding-Induced Spin–Orbit Coupling Enhancement for Efficient Pure Organic Room-Temperature Phosphorescence. J. Phys. Chem. Lett. 2022, 13, 1563–1570. 10.1021/acs.jpclett.1c04180. 35138107

[ref15] Lei Y.; Dai W.; Tian Y.; Yang J.; Li P.; Shi J.; Tong B.; Cai Z.; Dong Y. Revealing Insight into Long-Lived Room-Temperature Phosphorescence of Host–Guest Systems. J. Phys. Chem. Lett. 2019, 10, 6019–6025. 10.1021/acs.jpclett.9b02411. 31545040

[ref16] Yang J.; Wu X.; Shi J.; Tong B.; Lei Y.; Cai Z.; Dong Y. Achieving Efficient Phosphorescence and Mechanoluminescence in Organic Host–Guest System by Energy Transfer. Adv. Funct. Mater. 2021, 31, 2108072 10.1002/adfm.202108072.

[ref17] Fateminia S. M. A.; Mao Z.; Xu S.; Yang Z.; Chi Z.; Liu B. Organic Nanocrystals with Bright Red Persistent Room-Temperature Phosphorescence for Biological Applications. Angew. Chem. 2017, 129, 12328–12332. 10.1002/ange.201705945. 28771963

[ref18] Dai W.; Zhang Y.; Wu X.; Guo S.; Ma J.; Shi J.; Tong B.; Cai Z.; Xie H.; Dong Y. Red-Emissive Organic Room-Temperature Phosphorescence Material for Time-Resolved Luminescence Bioimaging. CCS Chem. 2022, 4, 2550–2559. 10.31635/ccschem.021.202101120.

[ref19] Ono T.; Kimura K.; Ihara M.; Yamanaka Y.; Sasaki M.; Mori H.; Hisaeda Y. Room-Temperature Phosphorescence Emitters Exhibiting Red to Near-Infrared Emission Derived from Intermolecular Charge-Transfer Triplet States of Naphthalenediimide–Halobenzoate Triad Molecules. Chem. – Eur. J. 2021, 27, 9535–9541. 10.1002/chem.202100906. 33780081

[ref20] Xiao F.; Gao H.; Lei Y.; Dai W.; Liu M.; Zheng X.; Cai Z.; Huang X.; Wu H.; Ding D. Guest-Host Doped Strategy for Constructing Ultralong-Lifetime near-Infrared Organic Phosphorescence Materials for Bioimaging. Nat. Commun. 2022, 13, 186 10.1038/s41467-021-27914-0. 35013474 PMC8748955

[ref21] Zhang X.; Du L.; Zhao W.; Zhao Z.; Xiong Y.; He X.; Gao P. F.; Alam P.; Wang C.; Li Z.; Leng J.; Liu J.; Zhou C.; Lam J. W. Y.; Phillips D. L.; Zhang G.; Tang B. Z. Ultralong UV/Mechano-Excited Room Temperature Phosphorescence from Purely Organic Cluster Excitons. Nat. Commun. 2019, 10, 5161 10.1038/s41467-019-13048-x. 31727890 PMC6856348

[ref22] Mao Z.; Yang Z.; Xu C.; Xie Z.; Jiang L.; Long Gu F.; Zhao J.; Zhang Y.; P Aldred M.; Chi Z. Two-Photon-Excited Ultralong Organic Room Temperature Phosphorescence by Dual-Channel Triplet Harvesting. Chem. Sci. 2019, 10, 7352–7357. 10.1039/C9SC02282A. 31489156 PMC6713867

[ref23] Alam P.; Cheung T. S.; Leung N. L. C.; Zhang J.; Guo J.; Du L.; Kwok R. T. K.; Lam J. W. Y.; Zeng Z.; Phillips D. L.; Sung H. H. Y.; Williams I. D.; Tang B. Z. Organic Long-Persistent Luminescence from a Single-Component Aggregate. J. Am. Chem. Soc. 2022, 144, 3050–3062. 10.1021/jacs.1c11480. 35049304

[ref24] Yang J.; Zhen X.; Wang B.; Gao X.; Ren Z.; Wang J.; Xie Y.; Li J.; Peng Q.; Pu K.; Li Z. The Influence of the Molecular Packing on the Room Temperature Phosphorescence of Purely Organic Luminogens. Nat. Commun. 2018, 9, 840 10.1038/s41467-018-03236-6. 29483501 PMC5826932

[ref25] Tian S.; Ma H.; Wang X.; Lv A.; Shi H.; Geng Y.; Li J.; Liang F.; Su Z.-M.; An Z.; Huang W. Utilizing d–Pπ Bonds for Ultralong Organic Phosphorescence. Angew. Chem., Int. Ed. 2019, 58, 6645–6649. 10.1002/anie.201901546. 30801896

[ref26] Wang Y.; Yang J.; Tian Y.; Fang M.; Liao Q.; Wang L.; Hu W.; Zhong Tang B.; Li Z. Persistent Organic Room Temperature Phosphorescence: What Is the Role of Molecular Dimers?. Chem. Sci. 2020, 11, 833–838. 10.1039/C9SC04632A. PMC8146318 34123059

[ref27] Wang Y.; Yang J.; Fang M.; Gong Y.; Ren J.; Tu L.; Tang B. Z.; Li Z. New Phenothiazine Derivatives That Exhibit Photoinduced Room-Temperature Phosphorescence. Adv. Funct. Mater. 2021, 31, 2101719 10.1002/adfm.202101719.

[ref28] Gao M.; Tian Y.; Yang J.; Li X.; Fang M.; Li Z. The Same Molecule but a Different Molecular Conformation Results in a Different Room Temperature Phosphorescence in Phenothiazine Derivatives. J. Mater. Chem. C 2021, 9, 15375–15380. 10.1039/D1TC03460J.

[ref29] Ekbote A.; Mobin S. M.; Misra R. Stimuli-Responsive Phenothiazine-Based Donor–Acceptor Isomers: AIE, Mechanochromism and Polymorphism. J. Mater. Chem. C 2020, 8, 3589–3602. 10.1039/C9TC05192A.

[ref30] Dai W.; Bianconi T.; Ferraguzzi E.; Wu X.; Lei Y.; Shi J.; Tong B.; Carlotti B.; Cai Z.; Dong Y. Excited-State Modulation of Aggregation-Induced Emission Molecules for High-Efficiency Triplet Exciton Generation. ACS Mater. Lett. 2021, 3, 1767–1777. 10.1021/acsmaterialslett.1c00528.

[ref31] Chen Y.; Lam J. W. Y.; Kwok R. T. K.; Liu B.; Tang B. Z. Aggregation-Induced Emission: Fundamental Understanding and Future Developments. Mater. Horiz. 2019, 6, 428–433. 10.1039/C8MH01331D.

[ref32] Zhao Z.; Zhang H.; Lam J. W. Y.; Tang B. Z. Aggregation-Induced Emission: New Vistas at the Aggregate Level. Angew. Chem., Int. Ed. 2020, 59, 9888–9907. 10.1002/anie.201916729. 32048428

[ref33] Chen B.; Zhang X.; Wang Y.; Miao H.; Zhang G. Aggregation-Induced Emission with Long-Lived Room-Temperature Phosphorescence from Methylene-Linked Organic Donor–Acceptor Structures. Chem. – Asian J. 2019, 14, 751–754. 10.1002/asia.201801002. 30025201

[ref34] Wang C.; Chen Y.; Xu Y.; Ran G.; He Y.; Song Q. Aggregation-Induced Room-Temperature Phosphorescence Obtained from Water-Dispersible Carbon Dot-Based Composite Materials. ACS Appl. Mater. Interfaces 2020, 12, 10791–10800. 10.1021/acsami.9b20500. 32037791

[ref35] Zhuang W.; Yang L.; Ma B.; Kong Q.; Li G.; Wang Y.; Tang B. Z. Multifunctional Two-Photon AIE Luminogens for Highly Mitochondria-Specific Bioimaging and Efficient Photodynamic Therapy. ACS Appl. Mater. Interfaces 2019, 11, 20715–20724. 10.1021/acsami.9b04813. 31144501

[ref36] Cao S.; Shao J.; Wu H.; Song S.; De Martino M. T.; Pijpers I. A. B.; Friedrich H.; Abdelmohsen L. K. E. A.; Williams D. S.; van Hest J. C. M. Photoactivated Nanomotors via Aggregation Induced Emission for Enhanced Phototherapy. Nat. Commun. 2021, 12, 2077 10.1038/s41467-021-22279-w. 33824321 PMC8024279

[ref37] Jiang R.; Dai J.; Dong X.; Wang Q.; Meng Z.; Guo J.; Yu Y.; Wang S.; Xia F.; Zhao Z.; Lou X.; Tang B. Z. Improving Image-Guided Surgical and Immunological Tumor Treatment Efficacy by Photothermal and Photodynamic Therapies Based on a Multifunctional NIR AIEgen. Adv. Mater. 2021, 33, 2101158 10.1002/adma.202101158. 33904232

[ref38] Liu Z.; Wang Q.; Qiu W.; Lyu Y.; Zhu Z.; Zhao X.; Zhu W.-H. AIE-Active Luminogens as Highly Efficient Free-Radical ROS Photogenerator for Image-Guided Photodynamic Therapy. Chem. Sci. 2022, 13, 3599–3608. 10.1039/D2SC00067A. 35432854 PMC8943840

[ref39] Xu Z.; Jiang Y.; Shen Y.; Tang L.; Hu Z.; Lin G.; Law W.-C.; Ma M.; Dong B.; Yong K.-T.; Xu G.; Tao Y.; Chen R.; Yang C. A Biocompatible Photosensitizer with a High Intersystem Crossing Efficiency for Precise Two-Photon Photodynamic Therapy. Mater. Horiz. 2022, 9, 1283–1292. 10.1039/D1MH01869H. 35170613

[ref40] Broadwater D.; Bates M.; Jayaram M.; Young M.; He J.; Raithel A. L.; Hamann T. W.; Zhang W.; Borhan B.; Lunt R. R.; Lunt S. Y. Modulating Cellular Cytotoxicity and Phototoxicity of Fluorescent Organic Salts through Counterion Pairing. Sci. Rep. 2019, 9, 15288 10.1038/s41598-019-51593-z. 31653966 PMC6814864

[ref41] Montalti M.; Credi A.; Prodi L.; Gandolfi M. T. Handbook of Photochemistry, 3rd ed.; CRC Press: Boca Raton, 2006.

[ref42] Schmidt R.; Tanielian C.; Dunsbach R.; Wolff C. Phenalenone, a Universal Reference Compound for the Determination of Quantum Yields of Singlet Oxygen O2(1Δg) Sensitization. J. Photochem. Photobiol. A 1994, 79, 11–17. 10.1016/1010-6030(93)03746-4.

[ref43] Ciorba S.; Carlotti B.; Škorić I.; Šindler-Kulyk M.; Spalletti A. Spectral Properties and Photobehaviour of 2,5-Distyrylfuran Derivatives. J. Photochem. Photobiol. A 2011, 219, 1–9. 10.1016/j.jphotochem.2011.01.009.

[ref44] Ortica F.; Romani A.; Favaro G. Light-Induced Hydrogen Abstraction from Isobutanol by Thienyl Phenyl, Dithienyl, and Thienyl Pyridyl Ketones. J. Phys. Chem. A 1999, 103, 1335–1341. 10.1021/jp983839y.

[ref45] Carmichael I.; Hug G. L. Triplet–Triplet Absorption Spectra of Organic Molecules in Condensed Phases. J. Phys. Chem. Ref. Data 1986, 15, 1–250. 10.1063/1.555770.

[ref46] Carlotti B.; Poddar M.; Elisei F.; Spalletti A.; Misra R. Energy-Transfer and Charge-Transfer Dynamics in Highly Fluorescent Naphthalimide–BODIPY Dyads: Effect of BODIPY Orientation. J. Phys. Chem. C 2019, 123, 24362–24374. 10.1021/acs.jpcc.9b05851.

[ref47] Carlotti B.; Cesaretti A.; Gentili P. L.; Marrocchi A.; Elisei F.; Spalletti A. A Two Excited State Model to Explain the Peculiar Photobehaviour of a Flexible Quadrupolar D−π–D Anthracene Derivative. Phys. Chem. Chem. Phys. 2016, 18, 23389–23399. 10.1039/C6CP03985E. 27499254

[ref48] del Giacco T.; Carlotti B.; Solis S. D.; Barbafina A.; Elisei F. Steady-State and Time-Resolved Investigations of a Crown Thioether Conjugated with Methylacridinium and Its Complexes with Metal Ions. Phys. Chem. Chem. Phys. 2011, 13, 2188–2195. 10.1039/C0CP01411G. 21127778

[ref49] Cesaretti A.; Carlotti B.; Consiglio G.; Del Giacco T.; Spalletti A.; Elisei F. Inclusion of Two Push–Pull *N* -Methylpyridinium Salts in Anionic Surfactant Solutions: A Comprehensive Photophysical Investigation. J. Phys. Chem. B 2015, 119, 6658–6667. 10.1021/acs.jpcb.5b02336. 25945687

[ref50] Snellenburg J. J.; Laptenok S. P.; Seger R.; Mullen K. M.; van Stokkum I. H. M. **Glotaran**: A *Java* -Based Graphical User Interface for the *R* Package **TIMP**. J. Stat. Software 2012, 49, 1–22. 10.18637/jss.v049.i03.

[ref51] Calzoni E.; Cesaretti A.; Montegiove N.; Di Michele A.; Pellegrino R. M.; Emiliani C. HexA-Enzyme Coated Polymer Nanoparticles for the Development of a Drug-Delivery System in the Treatment of Sandhoff Lysosomal Storage Disease. J. Funct. Biomater. 2022, 13, 37 10.3390/jfb13020037. 35466219 PMC9036261

[ref52] Botti V.; Cesaretti A.; Ban Ž.; Crnolatac I.; Consiglio G.; Elisei F.; Piantanida I. Fine Structural Tuning of Styryl-Based Dyes for Fluorescence and CD-Based Sensing of Various Ds-DNA/RNA Sequences. Org. Biomol. Chem. 2019, 17, 8243–8258. 10.1039/C9OB01186B. 31464340

[ref53] Cesaretti A.; Mencaroni L.; Bonaccorso C.; Botti V.; Calzoni E.; Carlotti B.; Fortuna C. G.; Montegiove N.; Spalletti A.; Elisei F. Amphiphilicity-Controlled Localization of Red Emitting Bicationic Fluorophores in Tumor Cells Acting as Bio-Probes and Anticancer Drugs. Molecules 2022, 27, 3713 10.3390/molecules27123713. 35744843 PMC9230006

[ref54] LeBel C. P.; Ischiropoulos H.; Bondy S. C. Evaluation of the Probe 2′,7′-Dichlorofluorescin as an Indicator of Reactive Oxygen Species Formation and Oxidative Stress. Chem. Res. Toxicol. 1992, 5, 227–231. 10.1021/tx00026a012. 1322737

[ref55] Rout Y.; Montanari C.; Pasciucco E.; Misra R.; Carlotti B. Tuning the Fluorescence and the Intramolecular Charge Transfer of Phenothiazine Dipolar and Quadrupolar Derivatives by Oxygen Functionalization. J. Am. Chem. Soc. 2021, 143, 9933–9943. 10.1021/jacs.1c04173. 34161725 PMC8297855

[ref56] Poddar M.; Cesaretti A.; Ferraguzzi E.; Carlotti B.; Misra R. Singlet and Triplet Excited-State Dynamics of 3,7-Bis(Arylethynyl)Phenothiazines: Intramolecular Charge Transfer and Reverse Intersystem Crossing. J. Phys. Chem. C 2020, 124, 17864–17878. 10.1021/acs.jpcc.0c01786.

[ref57] Rout Y.; Cesaretti A.; Ferraguzzi E.; Carlotti B.; Misra R. Multiple Intramolecular Charge Transfers in Multimodular Donor–Acceptor Chromophores with Large Two-Photon Absorption. J. Phys. Chem. C 2020, 124, 24631–24643. 10.1021/acs.jpcc.0c07616.

[ref58] Dong Y.; Sukhanov A. A.; Zhao J.; Elmali A.; Li X.; Dick B.; Karatay A.; Voronkova V. K. Spin–Orbit Charge-Transfer Intersystem Crossing (SOCT-ISC) in Bodipy-Phenoxazine Dyads: Effect of Chromophore Orientation and Conformation Restriction on the Photophysical Properties. J. Phys. Chem. C 2019, 123, 22793–22811. 10.1021/acs.jpcc.9b06170.

[ref59] Imran M.; Pang J.; Zhao J.; Li M.-D. Efficient Symmetry Breaking Spin–Orbit Charge Transfer-Induced Intersystem Crossing in Compact Orthogonal Perylene-Phenothiazine or -Phenoxazine Triads and Observation of the Delayed Fluorescence. J. Mater. Chem. C 2022, 10, 9758–9772. 10.1039/D2TC01640K.

[ref60] Cesaretti A.; Bianconi T.; Coccimiglio M.; Montegiove N.; Rout Y.; Gentili P. L.; Misra R.; Carlotti B. Aggregation-Induced Emission in Phenothiazine-Based Fluorophores: An Insight into the Excited State and Aggregate Formation Mechanism. J. Phys. Chem. C 2022, 126, 10429–10440. 10.1021/acs.jpcc.2c01423.

[ref61] Iwakiri S.; Hasegawa R.; Kubo Y. Near-Infrared Room-Temperature Phosphorescence in Arylselanyl BODIPY-Doped Materials. ChemPhotoChem 2022, 6, e202200073 10.1002/cptc.202200073.

