# Supplementary material for: Room-Temperature Phosphorescence and Cellular Phototoxicity Activated by Triplet Dynamics in Aggregates of Push–Pull Phenothiazine-Based Isomers
Source: J Phys Chem B. 2023 Feb 3;127(6):1385–98. doi: 10.1021/acs.jpcb.2c07717 (PMC9940226; doi:10.1021/acs.jpcb.2c07717)

SUPPORTING INFORMATION

Room Temperature Phosphorescence and Cellular Phototoxicity Activated by Triplet Dynamics in Aggregates of Push-Pull Phenothiazine-Based Isomers

Tommaso Bianconi,[1] Alessio Cesaretti,[1] Pietro Mancini,[1] Nicolò Montegiove,[1] Eleonora Calzoni,[1] Anupama Ekbote,[2] Rajneesh Misra,[2,*] Benedetta Carlotti[1,*]

[1] Department of Chemistry, Biology and Biotechnology, University of Perugia, via Elce di Sotto 8, 06123 Perugia, Italy. E-mail: benedetta.carlotti@unipg.it

[2] Department of Chemistry, Indian Institute of Technology, Indore 453552, India. E-mail: rajneeshmisra@iiti.ac.in

Fluorescence and Triplet Properties

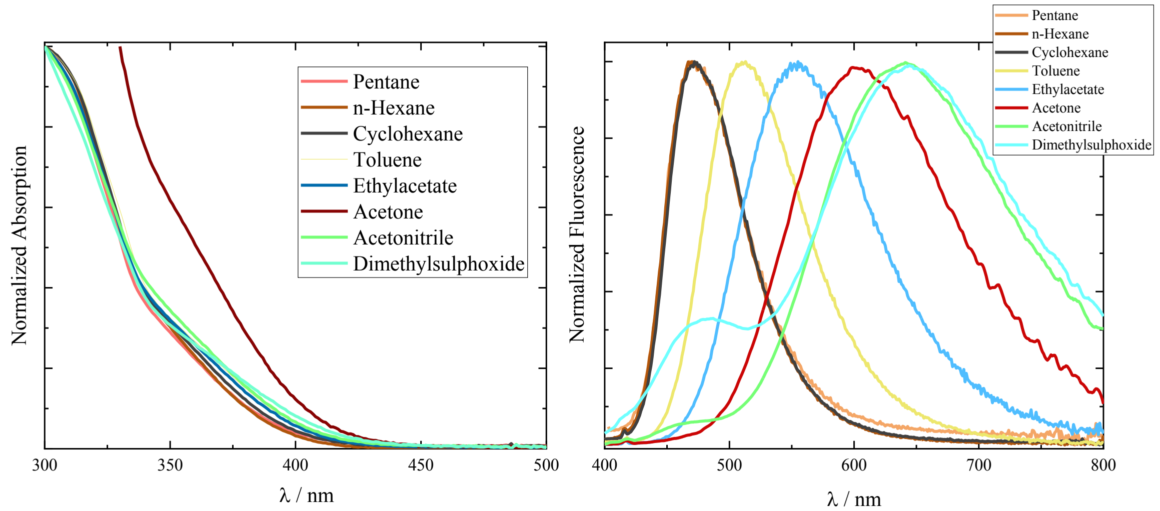

Figure S1. Solvent effect on the absorption (left) and emission (right) spectra of **o-PTZ**.

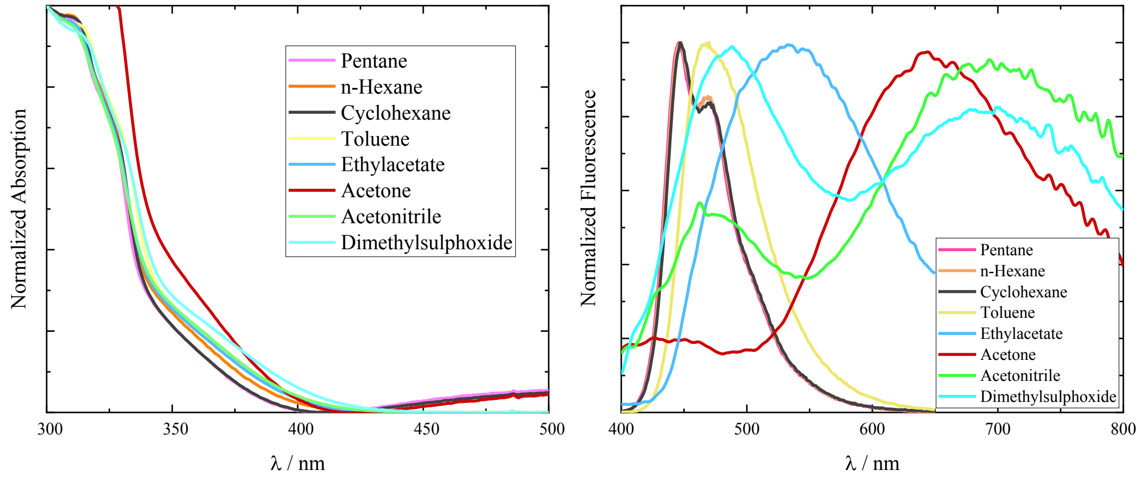

Figure S2. Solvent effect on the absorption (left) and emission (right) spectra of **m-PTZ**.

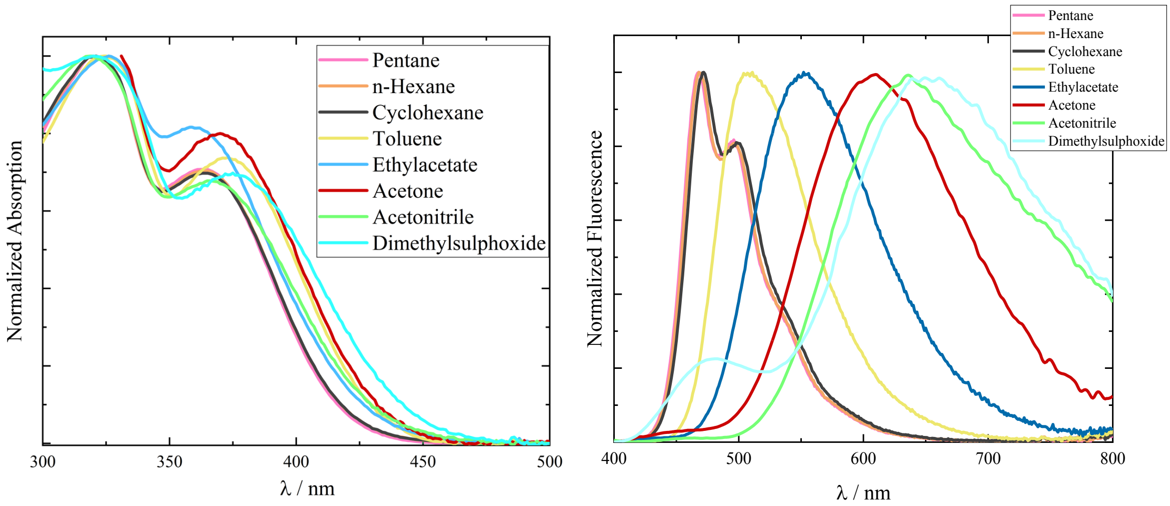

Figure S3. Solvent effect on the absorption (left) and emission (right) spectra of **p-PTZ**.

o-PTZ

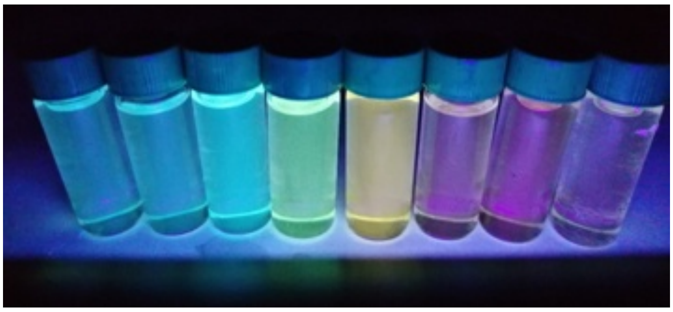

m-PTZ

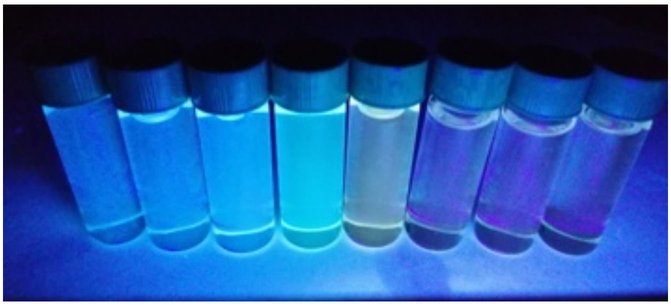

p-PTZ

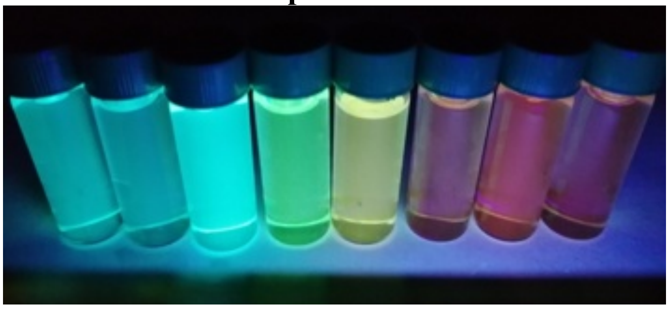

Figure S4. Fluorescence of the investigated isomers under UV light in solvents of different polarity. From left to right: pentane, n-hexane, cyclohexane, toluene, ethylacetate, acetone, acetonitrile, dimethylsulphoxide.

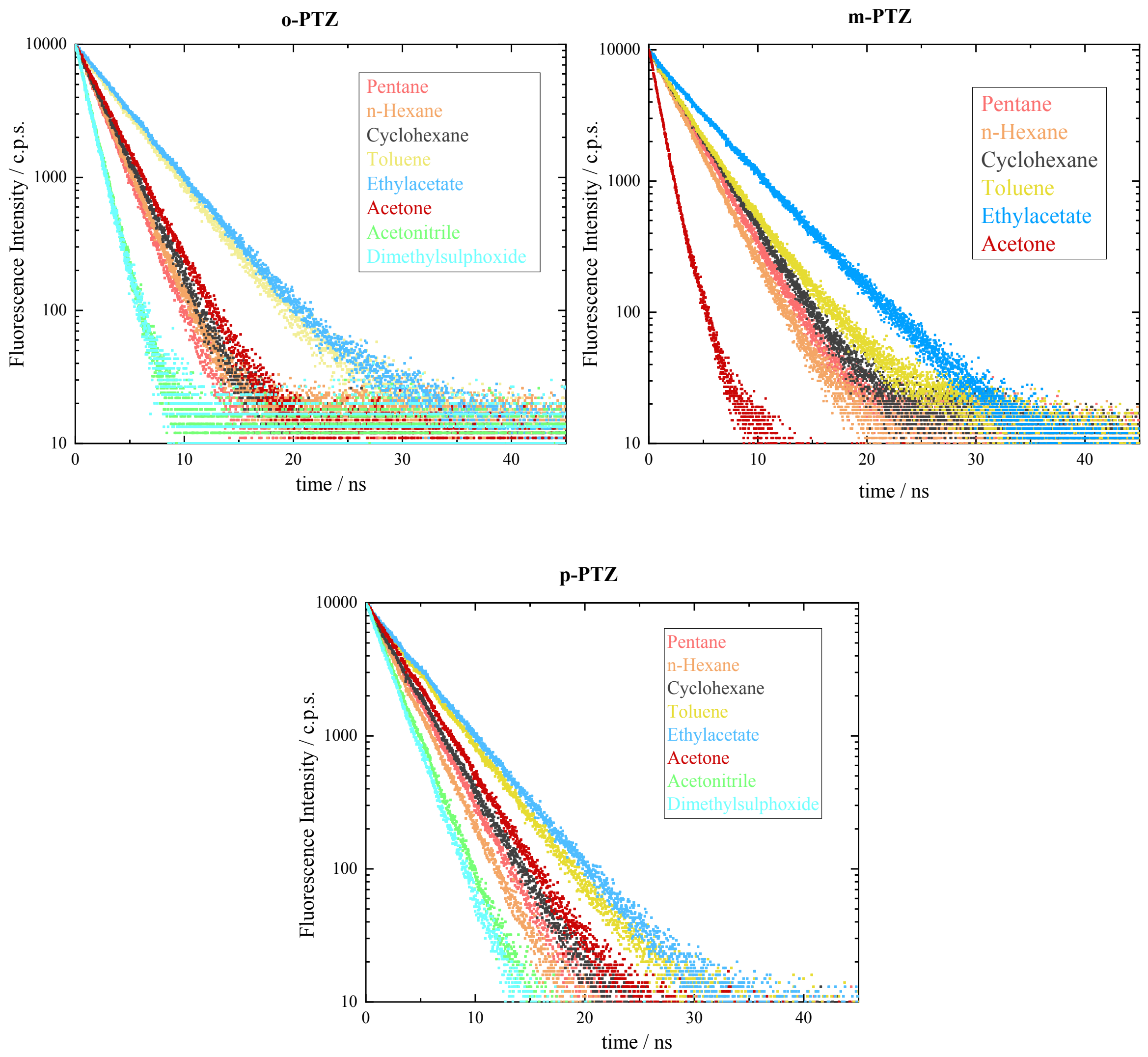

Figure S5. Fluorescence decay kinetics obtained by ns TC-SPC for the three isomers in different solvents.

Table S1. Fluorescence properties of the investigated isomers in solvents of different polarity.

	**o-PTZ**			**m-PTZ**			**p-PTZ**		
Solvent	ϕ_F	τ_F / ns	k_F/ s^{-1}	ϕ_F	τ_F / ns	k_F/ s^{-1}	ϕ_F	τ_F / ns	k_F/ s^{-1}
Pentane	0.037	1.95	1.9×10^7	0.15	2.79	5.2×10^7	0.42	2.78	1.5×10^8
n-Hexane	0.12	2.21	5.4×10^7	0.11	2.55	4.3×10^7	0.39	2.50	1.6×10^8
Me-Cyclohexane/ 3-Me-Pentane (9:1)	0.14	2.44	5.7×10^7	0.17	2.94	5.8×10^7	0.53	2.88	1.8×10^8
Cyclohexane	0.17	2.38	7.1×10^7	0.11	3.05	3.6×10^7	0.54	3.01	1.8×10^8
Toluene	0.19	4.17	4.6×10^7	0.13	3.49	3.7×10^7	0.81	3.97	2.0×10^8
Ethyl Acetate	0.18	4.30	4.2×10^7	0.10	4.62	2.2×10^7	0.70	4.28	1.6×10^8
Acetone	0.062	2.65	2.3×10^7	0.0064	1.04	6.2×10^6	0.39	3.24	1.2×10^8
Acetonitrile	0.024	1.16	2.1×10^7	0.0052	-	-	0.21	2.01	1.0×10^8
Dimethylsulphoxide	0.018	1.10	1.6×10^7	0.0040	0.22*	1.8×10^7	0.22	1.88	1.1×10^8

*from femtosecond measurements; uncertainties are estimated to be about ±10% on ϕ_F and ± 5% on τ_F.

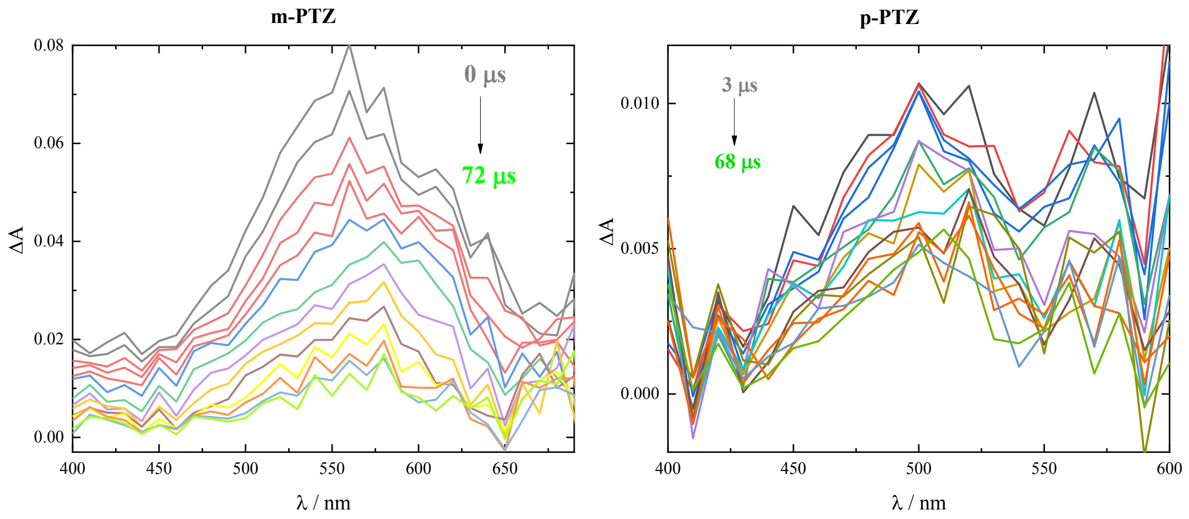

Figure S6. Triplet spectra obtained by ns laser flash photolysis for the investigated isomers in nitrogen purged Tol.

Table S2. Triplet properties of the investigated isomers in solvents of different polarity.

Sample	*Solvent*	λ_T / nm	$\tau_{T,air}$ / ns	$\tau_{T,N2}$ / μs	$(\phi\times\varepsilon)_T$ / $M^{-1}cm^{-1}$	ε_T / $M^{-1}cm^{-1}$	ϕ_T
**o-PTZ**	Pent	550	27	1.4	7850		1.05
	Tol				4340		0.57
	DMSO				790	7500*	0.10
**m-PTZ**	Pent	560	33	18	14400		1.03
	Tol				12200		0.88
	DMSO	580			180	13950*	0.013
**p-PTZ**	Pent	500	35	21	4100		0.36
	Tol				1180		0.10
	DMSO				475	11300*	0.042

*in acetonitrile; uncertainties are estimated to be about ±15% on ϕ_T and ± 10% on $(\phi\times\varepsilon)_T$.

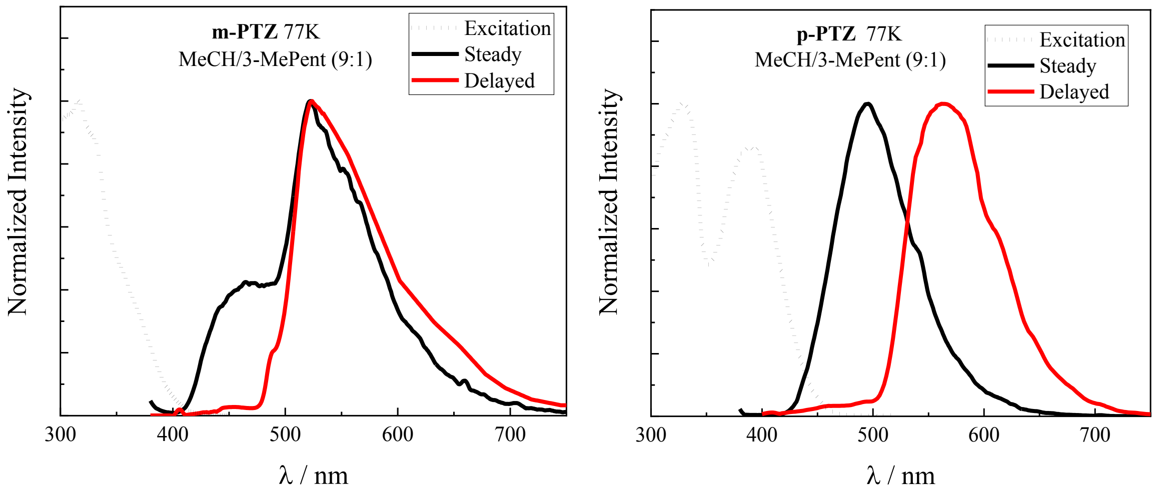

Figure S7. Excitation spectra (dotted line), steady (black line), and delayed (red line) emission of the isomers in MethylCyclohexane/3-MethylPentane mixture, at 77 K.

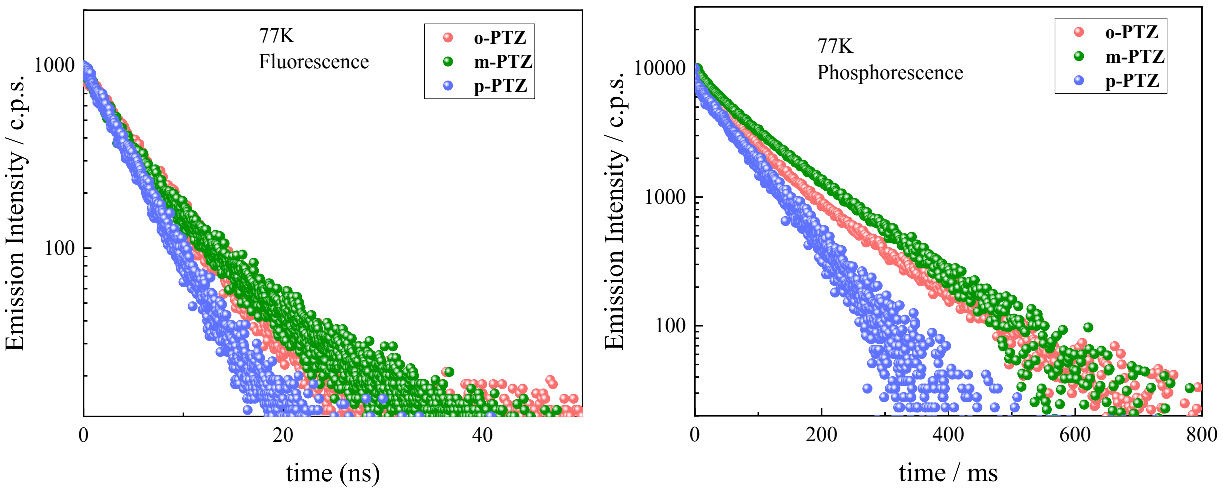

Figure S8. Fluorescence and phosphorescence decay in methylcyclohexane/3-methylpentane mixture, at 77 K, of **o-PTZ**, **m-PTZ**, and **p-PTZ**, respectively.

Table S3. Emission properties in methylcyclohexane/3-methylpentane mixture, at 77 K.

Compound	λ_{steady} / *nm*	$\lambda_{delayed}$/ *nm*	τ_{steady}/ *ns*	$\tau_{delayed}$/ *ms*
**o-PTZ**	506	541	4.08 (41%)<br>6.63 (59%)	49.5 (35%)<br>131 (65%)
**m-PTZ**	465	523	3.96 (40%)<br>11.3 (60%)	48.8 (16%)<br>131 (84%)
**p-PTZ**	494	565	4.32	72.3

Experimental uncertainty on the lifetime values is ca. ±5%.

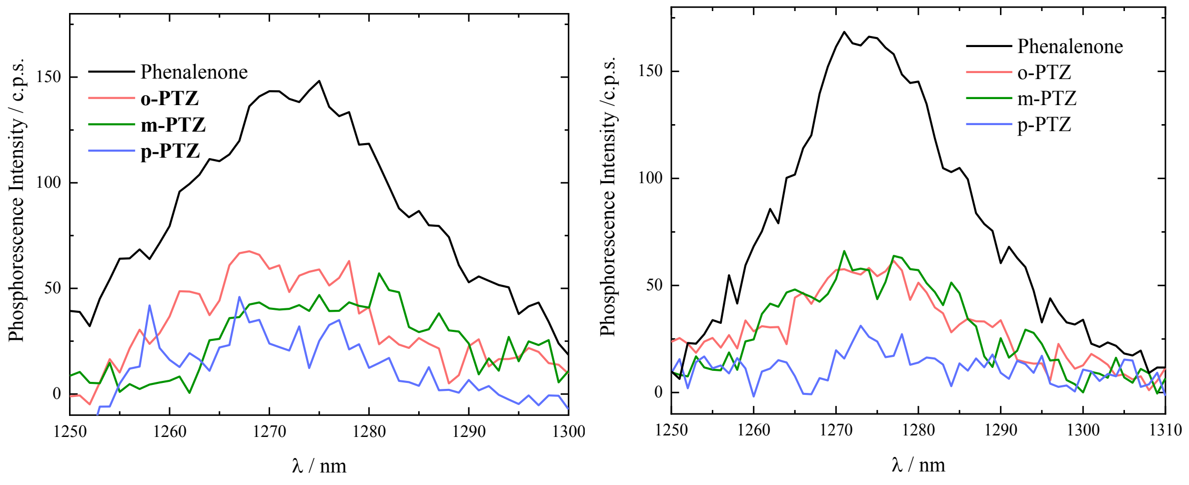

Figure S9. Singlet oxygen phosphorescence spectra in cyclohexane (left) and Tol (right).

Quantum Mechanical Calculations

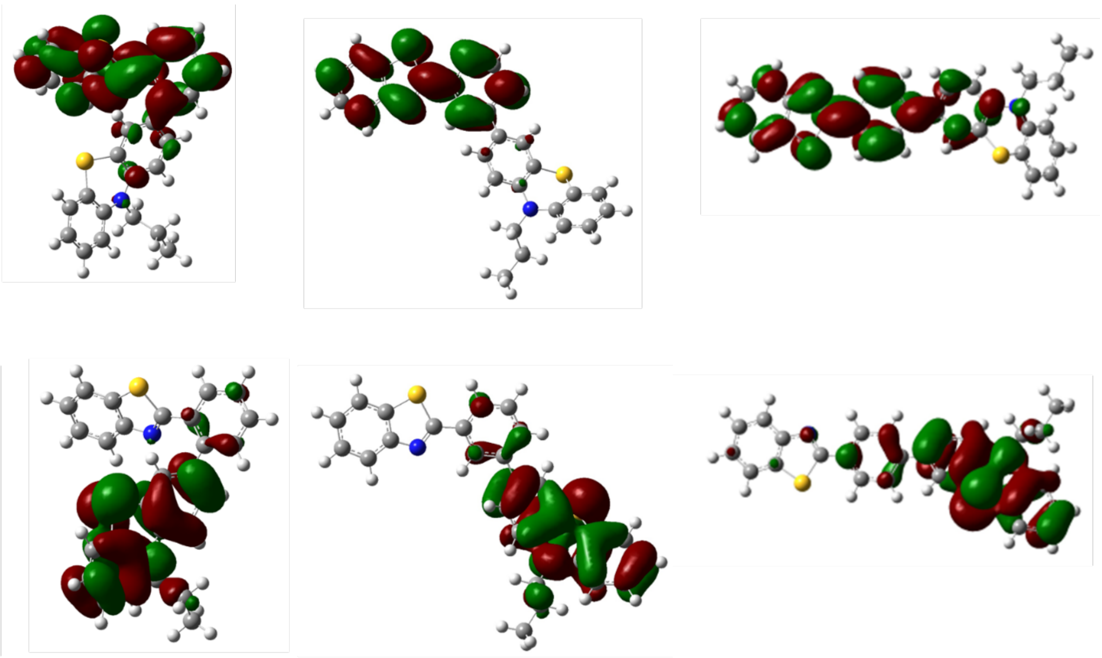

o-PTZ *m*-PTZ *p*-PTZ

Figure S10. HOMO (bottom) and LUMO (top) of ***o*-PTZ**, ***m*-PTZ**, and ***p*-PTZ** as determined at the B3LYP/6-31G +(d,p) level on their ground state optimized structures.

o-PTZ *m*-PTZ *p*-PTZ

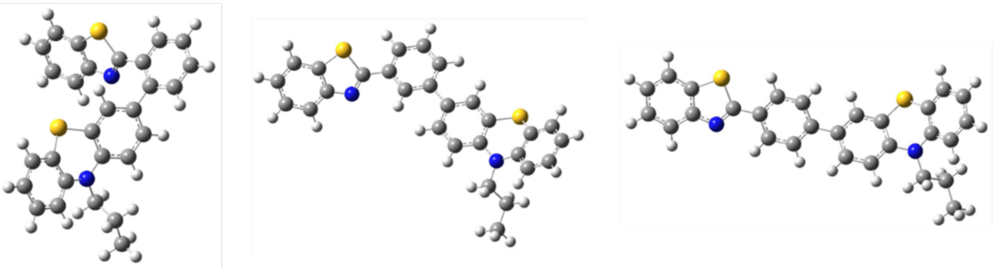

Isomer	PTZ-phenyl	Phenyl-benzothiazole
**o-PTZ**	53.36°	45.39°
***m*-PTZ**	38.47°	2.99°
***p*-PTZ**	37.93°	2.20°

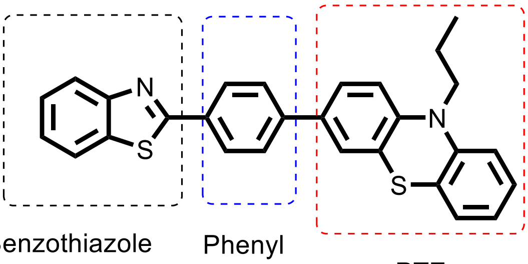

Figure S11. Optimized geometries for the ground state of ***o*-PTZ**, ***m*-PTZ**, and ***p*-PTZ** and detailed relative dihedral angles.

The solvatochromic method based on the McRae's theory (*J. Photochem. Photobiol. A, Chem.*, 2019, 368, 190–199; *Chem. Phys.*, 2012, 407, 9–19) was used to describe the linear correlation of the Stokes Shifts *vs.* the function of solvent properties, f(ε, n^2) equal to $\left(\frac{\varepsilon-1}{\varepsilon+2}+\frac{n^2-1}{n^2+1}\right)$, and to get the approximated difference between the excited and ground state dipole moments ($\Delta\mu = |\mu_{ES} - \mu_{GS}|$), according to the following equation:

$$\Delta\nu = \nu_{abs} - \nu_{em} = (\delta_{abs} + \delta_{em}) + \frac{2\Delta\mu^2}{hca^3}\left(\frac{\varepsilon-1}{\varepsilon+2}+\frac{n^2-1}{n^2+1}\right) \text{ Eq.1}$$

where $\Delta\nu = \nu_{abs} - \nu_{em}$ is the Stokes shift (in cm^{-1}); δ_{abs} and δ_{em} are the differences in the vibrational energy (in cm^{-1}) of the molecules in the excited and ground states, respectively; *a* is the cavity radius within Onsager's model (in cm), and taken as 60% of the calculated diameter along the charge transfer direction resulting from the optimized geometry; *h* is the Planck's constant (in erg × s) and *c* is the light speed (in cm × s^{-1}). In the table reported below the main results obtained from this data analysis are shown.

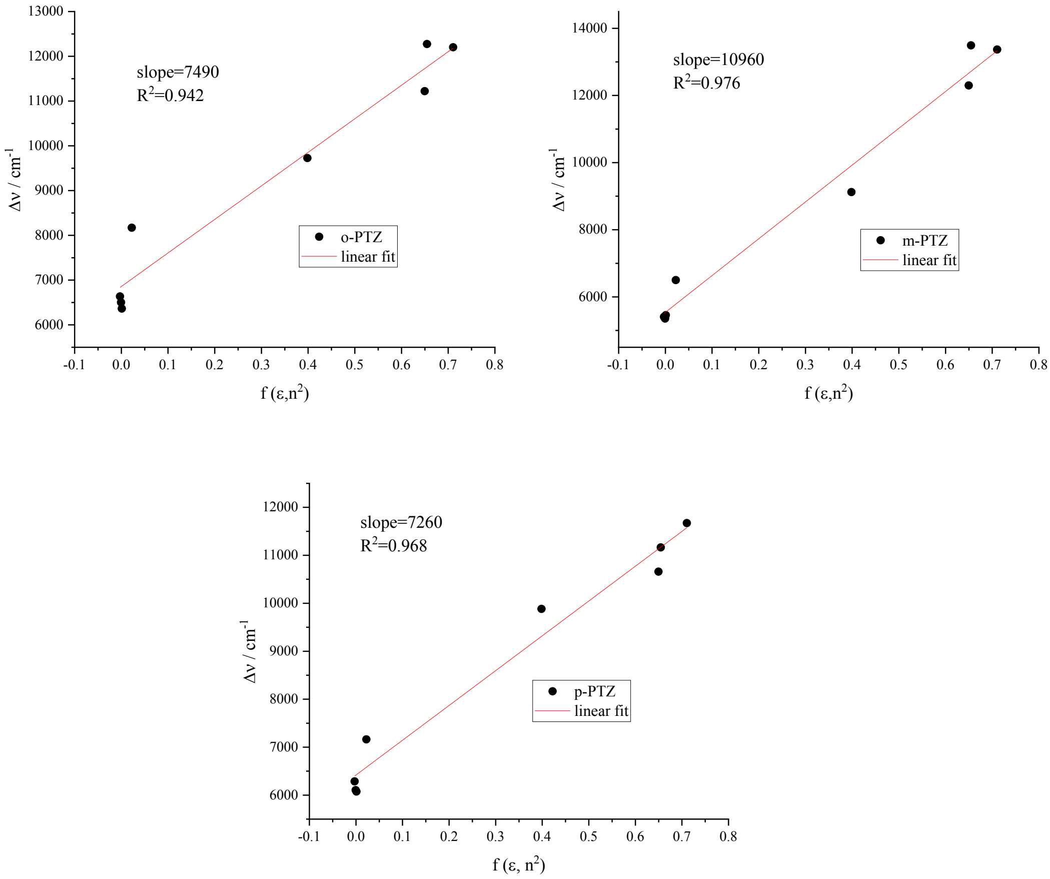

Figure S12. Plot of the Stokes shift as a function of f(ε, n^2) for the three isomers.

Table S4. Parameters for the calculation of the **Δμ** = dipole moment difference of the three investigated compounds (***a*** = Onsager radius; **slope** = from the linear fit of the Stokes Shifts *vs.* f(ε, n) plot).

**Compound**	**slope**	***a*** **(Å)**	**Δμ (D)**
**o-PTZ**	7490	5.98	12.6
**m-PTZ**	10960	9.78	31.9
**p-PTZ**	7260	10.3	28.0

Ultrafast Excited State Dynamics

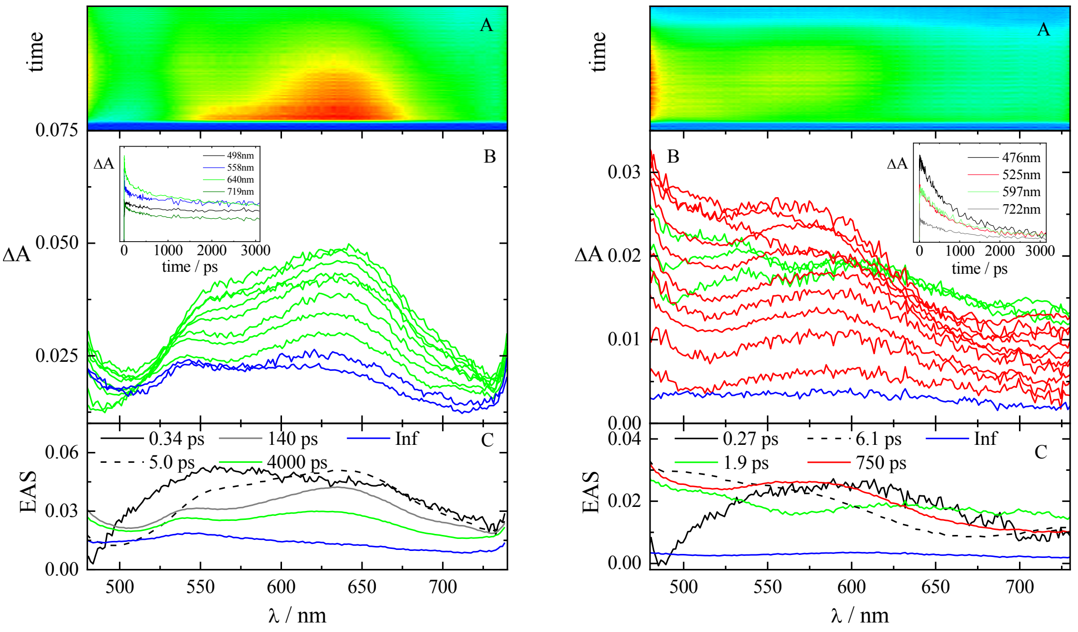

Figure S13. Femtosecond transient absorption spectroscopy of **o-PTZ** in Tol (left) and DMSO (right).

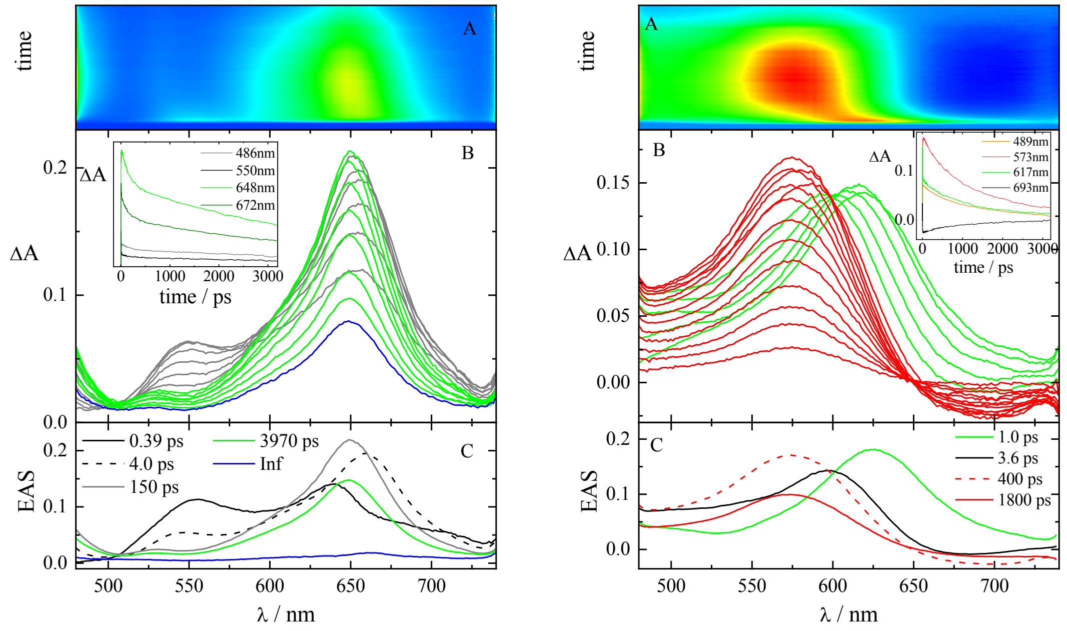

Figure S14. Femtosecond transient absorption spectroscopy of **p-PTZ** in Tol (left) and DMSO (right).

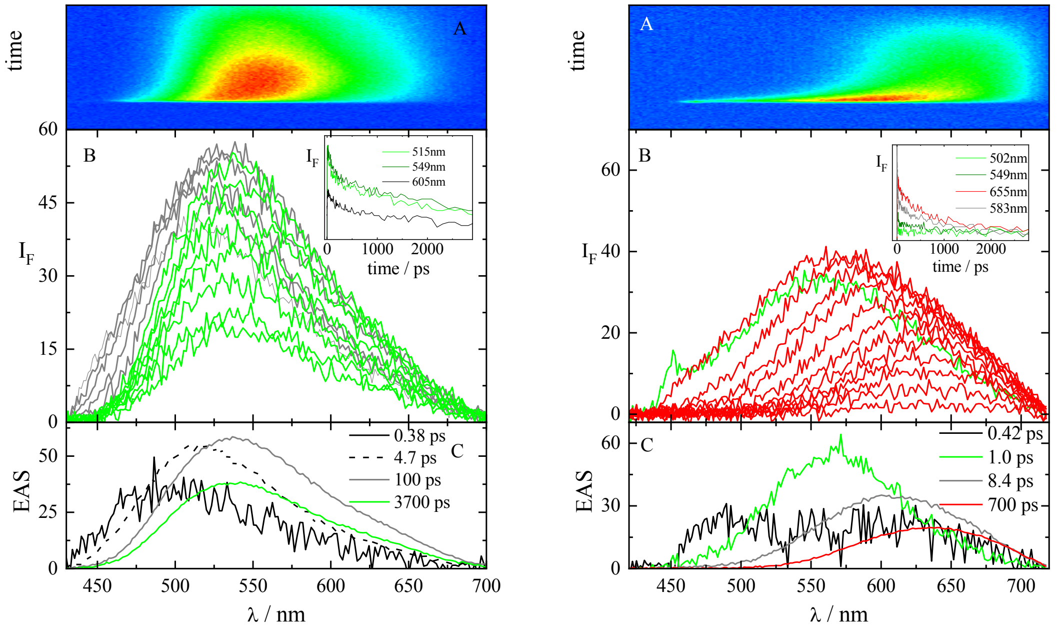

Figure S15. Femtosecond broadband fluorescence up-conversion spectroscopy of **o-PTZ** in Tol (left) and DMSO (right).

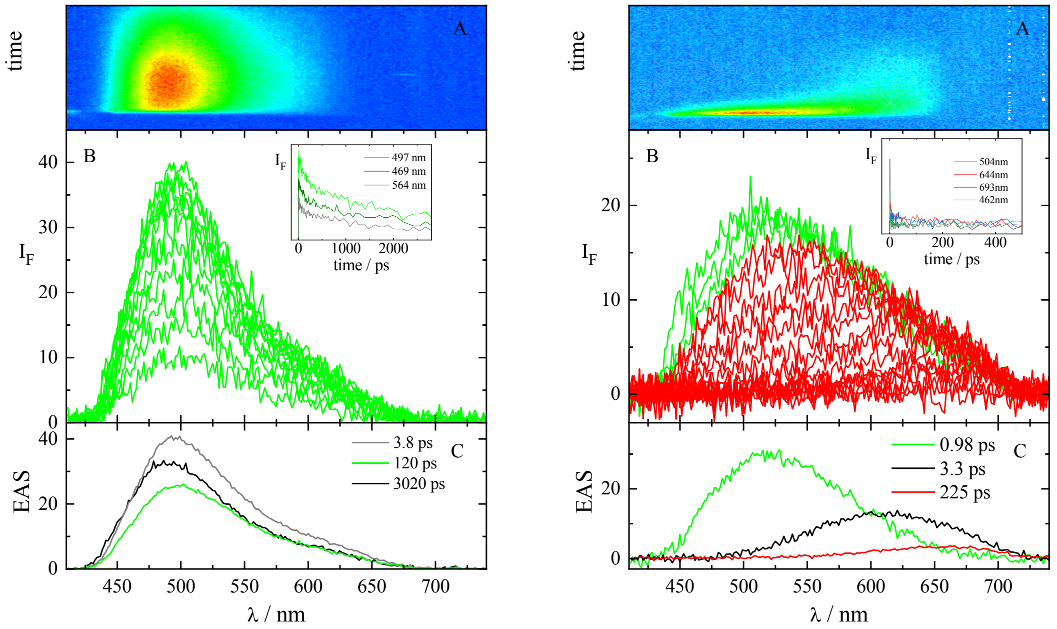

Figure S16. Femtosecond broadband fluorescence up-conversion spectroscopy of **m-PTZ** in Tol (left) and DMSO (right).

Aggregation Induced Emission

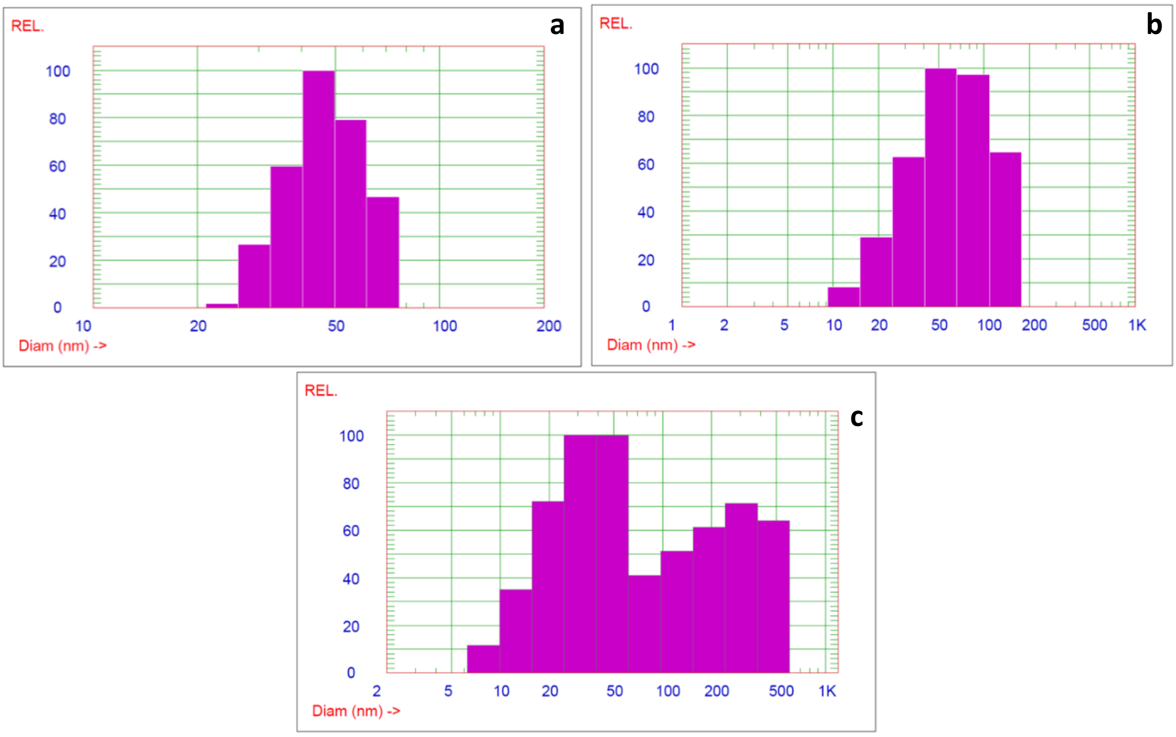

Figure S17. Dynamic Light Scattering particle size distribution obtained in DMSO/W mixtures (98.5% W) of (a) ortho, (b) meta and (c) para PTZ isomers.

Table S5. Aggregates dimension extrapolated from Dynamic Light Scattering measurements and Z-potential associated.

	DMSO/W mixtures (98.5% W)		
	**o-PTZ**	**m-PTZ**	**p-PTZ**
DLS particle size (nm)	48 ± 12	71 ± 36	37 ± 20<br>360 ± 110
Poly-Diversity Index*	0.063	0.26	>0.4
Z-potential (mV)	-24.9	-9.56	-10.6

*PDI=$(Standard\ Deviation\ /\ Average)^2$

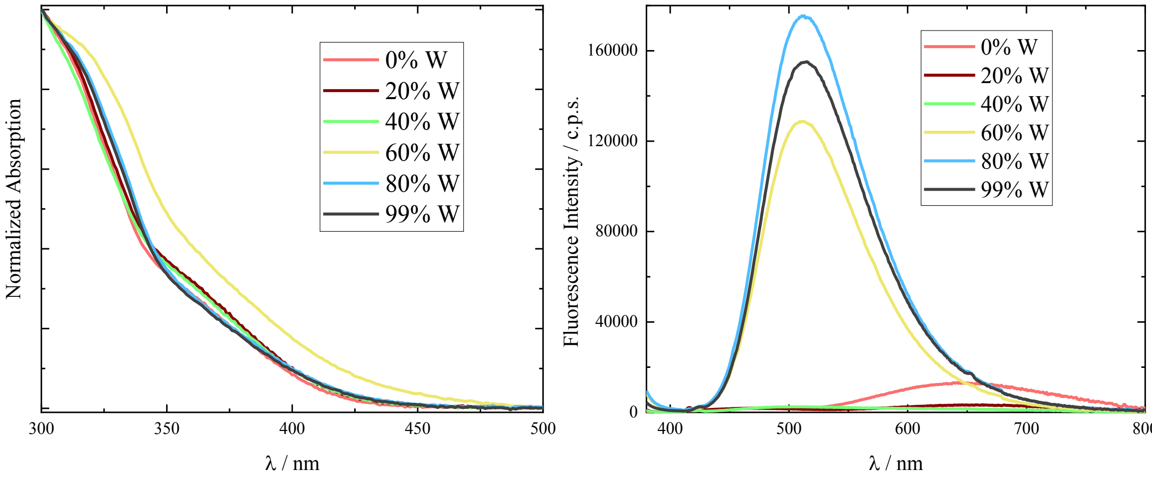

Figure S18. Absorption (left) and emission (right) spectra of **o-PTZ** in DMSO/W mixtures containing different water amounts.

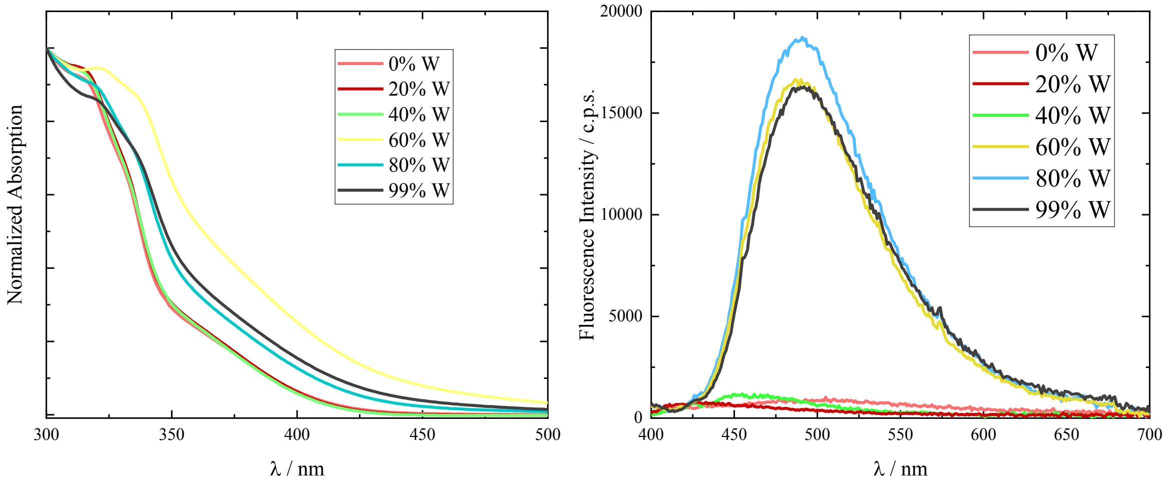

Figure S19. Absorption (left) and emission (right) spectra of **m-PTZ** in DMSO/W mixtures containing different water amounts.

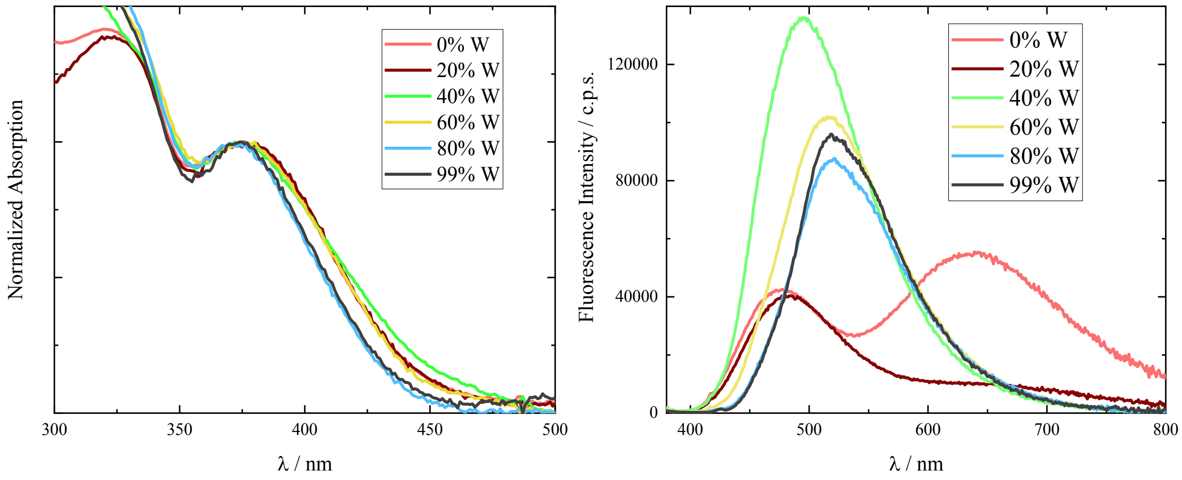

Figure S20. Absorption (left) and emission (right) spectra of **p-PTZ** in DMSO/W mixtures containing different water amounts.

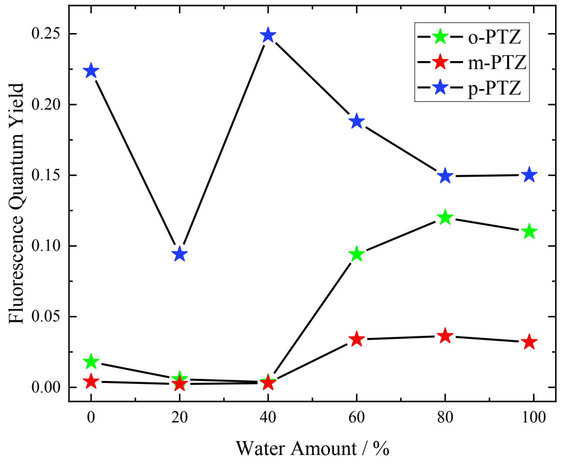

Figure S21. Fluorescence quantum yield of the investigated isomers in DMSO/W mixtures containing different water amounts.

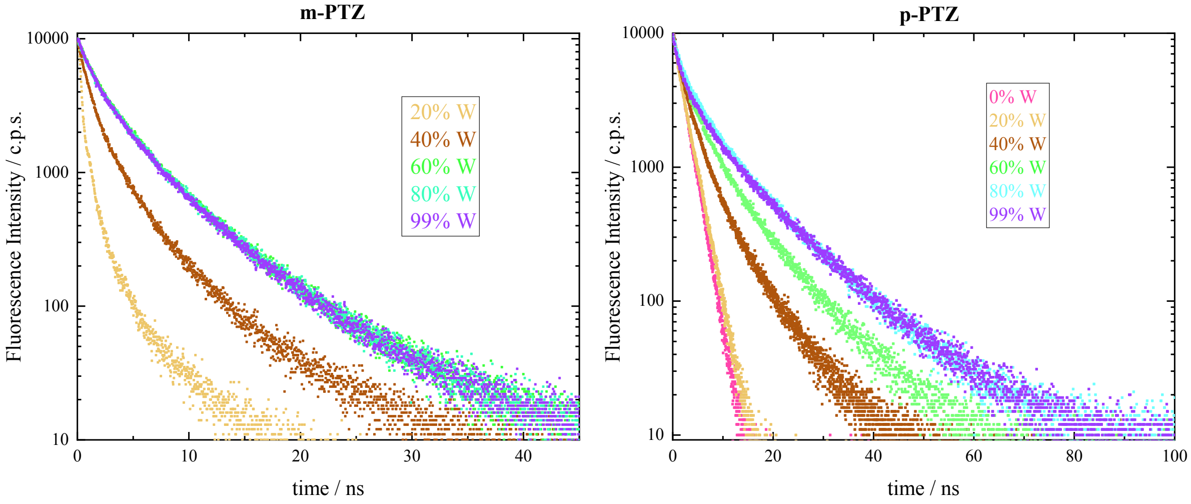

Figure S22. Fluorescence decay kinetics obtained by ns TC-SPC for the investigated isomers in DMSO/W mixtures containing different water amounts.

Table S6. Fluorescence properties of the investigated isomers in DMSO/W mixtures with different water amounts.

	o-PTZ				m-PTZ				p-PTZ			
Water	λ_F/nm	ϕ_F	τ_F/ns	k_F/s^{-1}	λ_F/nm	ϕ_F	τ_F/ns	k_F/s^{-1}	λ_F/nm	ϕ_F	τ_F/ns	k_F/s^{-1}
0%	462,644	0.018	1.10	1.6×10^7	500	0.0040	-	-	478,642	0.22	1.82	1.2×10^8
20%	478,658	0.0057	0.29(96%)<br>2.46(4%)	2.3×10^6	426	0.0024	0.11(65%)<br>1.12(26%)<br>6.17(9%)	4.0×10^5	484	0.094	2.13	4.4×10^7
40%	504	0.0037	0.38(15%)<br>2.00(43%)<br>4.80(42%)	8.0×10^5	457	0.0030	0.62(37%)<br>2.63(49%)<br>8.95(14%)	3.4×10^5	495	0.25	0.40(7%)<br>2.60(63%)<br>7.99(30%)	3.1×10^7
60%	511	0.094	0.72(6%)<br>3.40(46%)<br>6.60(48%)	1.4×10^7	490	0.034	0.31(10%)<br>2.70(51%)<br>7.66(39%)	4.4×10^6	516	0.19	0.78(10%)<br>3.53(48%)<br>10.3(42%)	1.8×10^7
80%	511	0.12	0.68(6%)<br>3.60(49%)<br>7.20(45%)	1.7×10^7	490	0.036	0.34(11%)<br>2.83(53%)<br>8.02(36%)	4.5×10^6	521	0.15	0.80(8%)<br>4.54(42%)<br>12.9(50%)	1.2×10^7
99%	515	0.11	0.61(8%)<br>3.20(49%)<br>7.00(43%)	1.6×10^7	490	0.032	0.29(10%)<br>2.72(53%)<br>7.86(37%)	4.0×10^6	519	0.15	0.70(10%)<br>4.23(37%)<br>13.2(53%)	1.1×10^7

Uncertainties are estimated to be about ±10% on ϕ_F and ± 5% on τ_F.

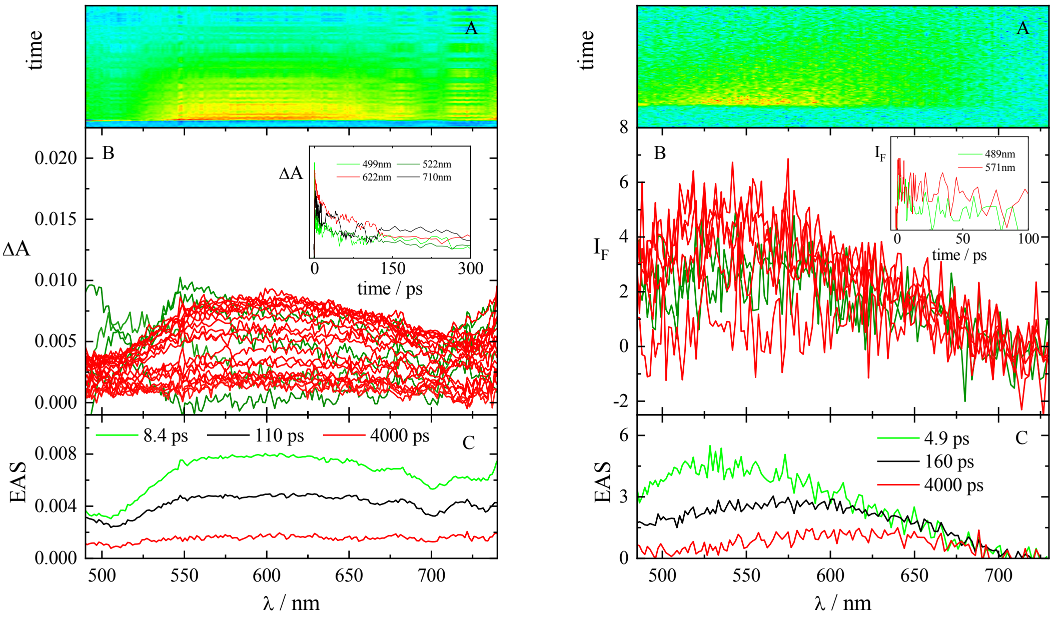

Figure S23. Femtosecond transient absorption (left) and broadband fluorescence up-conversion (right) spectroscopy of **o-PTZ** in a DMSO/W mixture with 80% W.

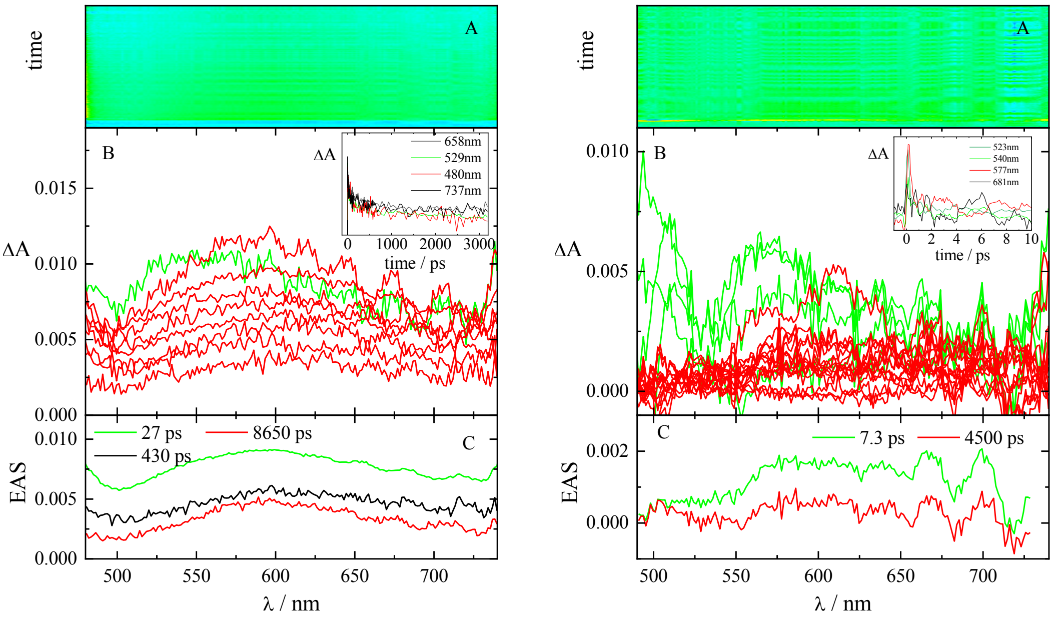

Figure S24. Femtosecond transient absorption spectroscopy of **m-PTZ** (left) and **p-PTZ** (right) in a DMSO/W mixture with 80% W.

Table S7. Results of Global Analysis of the femtosecond transient absorption (TA) and broadband fluorescence up-conversion (FUC) data for the investigated isomers in a DMSO/W mixture with 80% W.

Solvent	τ_{TA} / *ps*			τ_{FUC} / *ps*			*Assignment*
	**o-PTZ**	**m-PTZ**	**p-PTZ**	**o-PTZ**	**m-PTZ**	**p-PTZ**	
DMSO/W<br>20:80	8.4<br>110<br>***4000***	27<br>430<br>***8650***	7.3<br><br>***4500***	4.9<br>160<br>***4000***			S_1(LE)<br>SR<br>S_1(***PICT***)

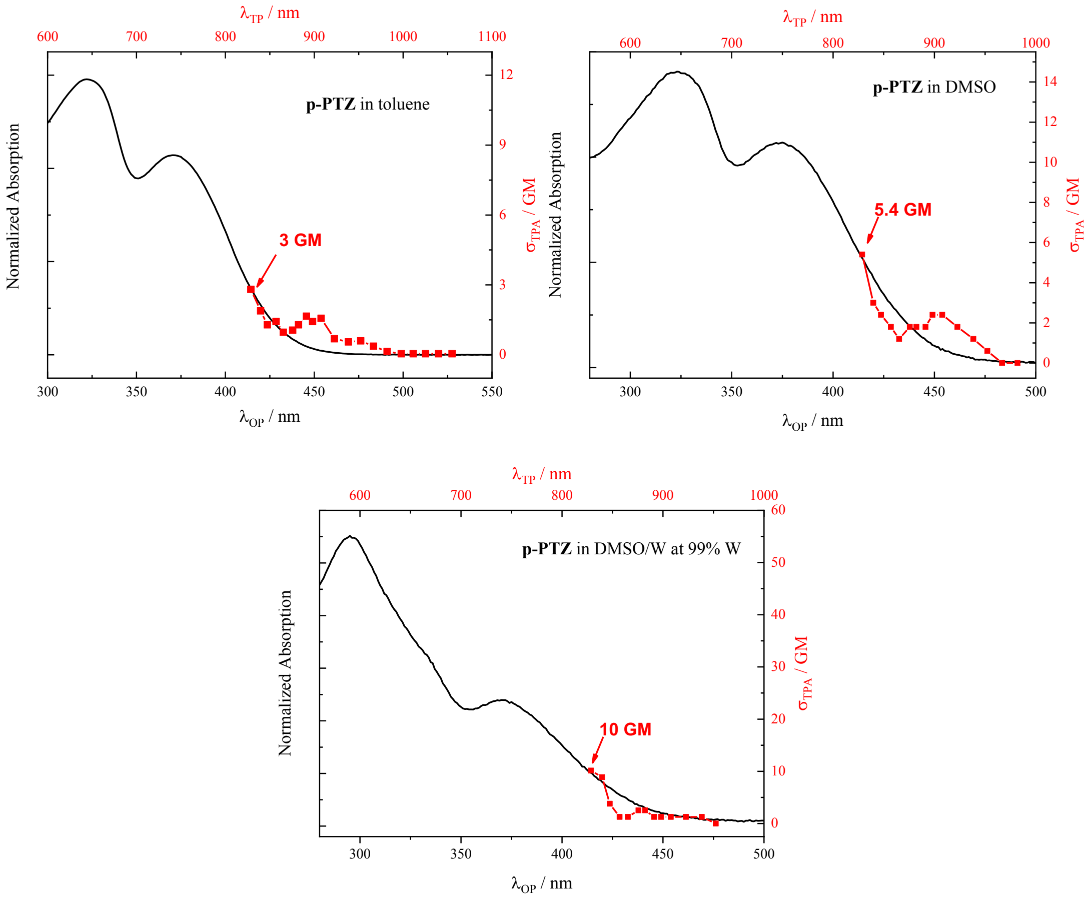

Figure S25. Two photon excitation spectra of **p-PTZ** in Tol, DMSO and DMSO/W at 99%W obtained through two photon excited fluorescence experiments.

Table S8. Two photon absorption cross sections of **p-PTZ** in Tol, DMSO and DMSO/W at 99%W obtained through two photon excited fluorescence experiments.

	σ_{TPA} / GM @ 830 nm
Tol	3.0
DMSO	5.4
99% W	10

Room Temperature Phosphorescence

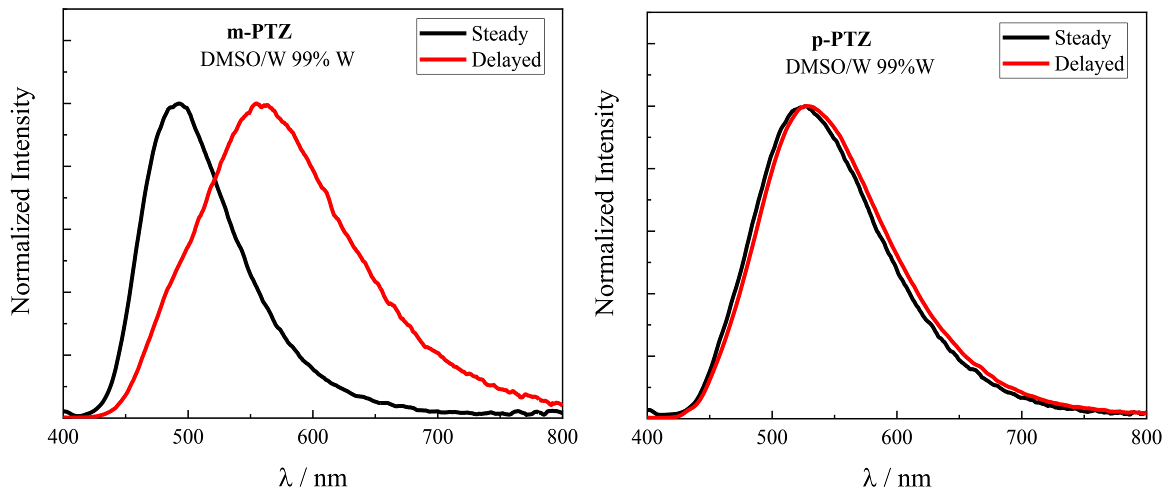

Figure S26. Steady (black line) and delayed (red line) emission spectra of the isomers in DMSO/W mixtures at 99% W.

Table S9. Emission properties of the isomers in DMSO/W mixtures at 99% W.

Compound	λ_{steady} / *nm*	$\lambda_{delayed}$ / *nm*	τ_{steady} / *ns*	$\tau_{delayed}$ / μs
**o-PTZ**	511	560	0.60 (6%)<br>3.57 (50%)<br>7.48 (44%)	33.6 (10%)<br>118 (49%)<br>353 (41%)
**m-PTZ**	493	559	0.29 (10%)<br>2.72 (53%)<br>7.86 (37%)	29.2 (37%)<br>82.1 (51%)<br>237 (12%)
**p-PTZ**	526	//	0.69 (10%)<br>4.23 (37%)<br>13.2 (53%)	5.3

Experimental uncertainty on the lifetime values is ca. ±5%.

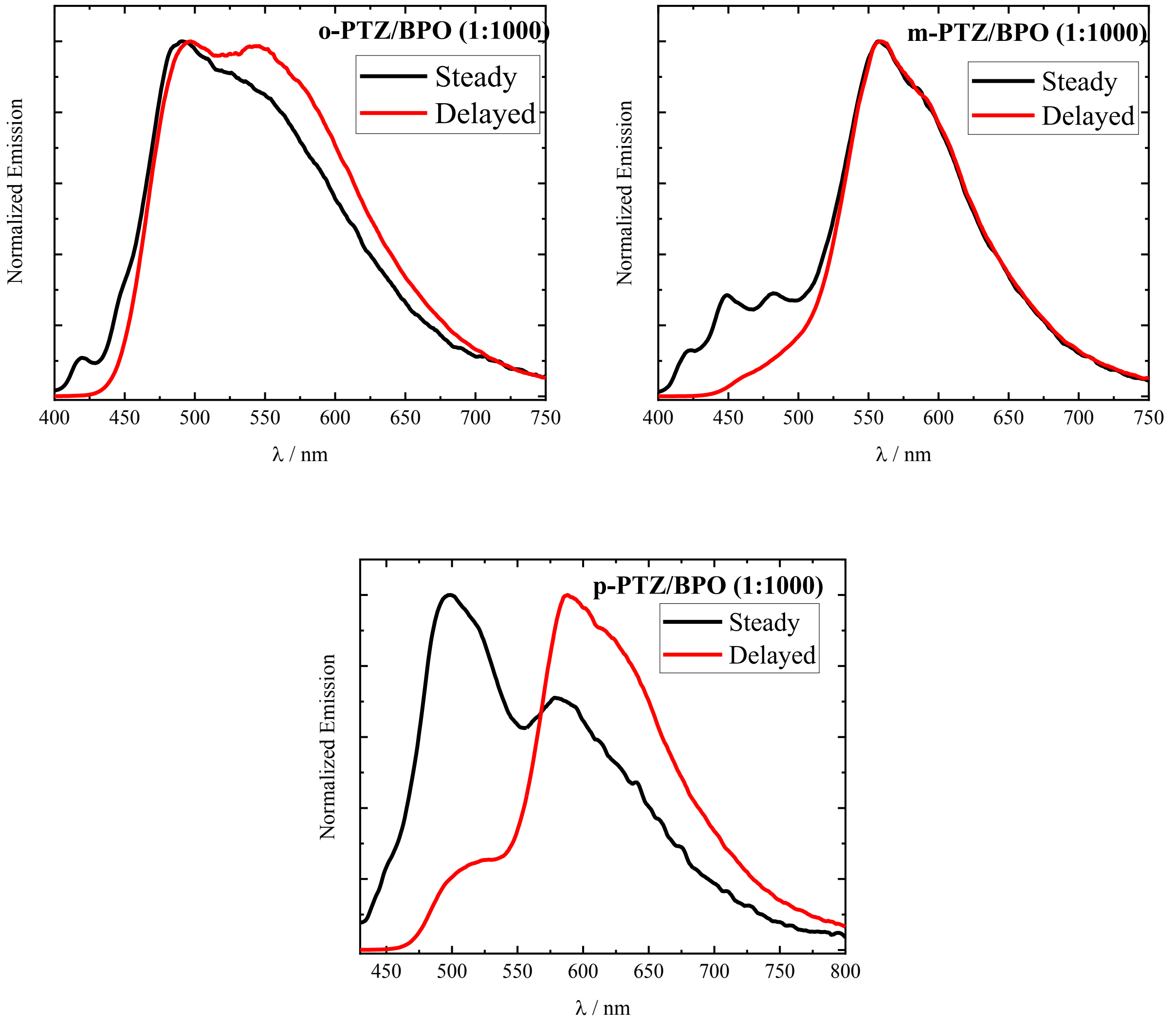

Figure S27. Steady-state and Delayed emission spectra for the guest isomers in the BPO host solid matrices.

Table S10. Emission properties of the guest isomers in the solid **TPP** and **BPO** host matrices.

Guest	*Host*	λ_{steady} / *nm*	$\lambda_{delayed}$ / *nm*	τ_{steady} / *ns*	$\tau_{delayed}$ /*ms*
**o-PTZ**	**TPP**	504	544	3.90 (57%)<br>7.34 (43%)	8.55 (45%)<br>27.1 (55%)
**m-PTZ**		480	554	2.56 (64%)<br>7.03 (36%)	20.7 (34%)<br>61.6 (66%)
**p-PTZ**		506	587	4.80 (69%)<br>10.6 (31%)	21.1 (45%)<br>36.1 (55%)
**o-PTZ**	**BPO**	489	544	//	35.7
**m-PTZ**		558	558	//	42.5
**p-PTZ**		499	587	0.76 (11%)<br>3.30 (53%)<br>7.69 (36%)	43.2

Experimental uncertainty on the lifetime values is ca. ±5%.

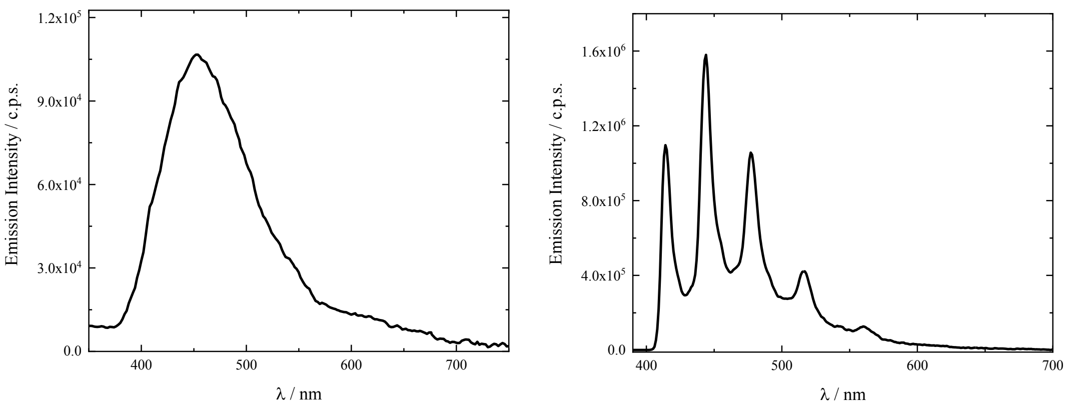

Figure S28. Phosphorescence spectra of **TPP** in MeCH/3-MePent and **BPO** in EPA at 77K.

Table S11. Emission properties in MeCH/3-MePent at 77K.

Compound	λ_F/nm (E_{S1} / eV)	λ_{Ph}/nm (E_{T1} / eV)
**TPP**	//	454 (2.73)
**BPO**	//	414 (3.00)<br>444<br>477<br>516
**o-PTZ**	506 (2.45)	541 (2.30)
**m-PTZ**	465 (2.66)	523 (2.37)
**p-PTZ**	494(2.50)	565 (2.19)

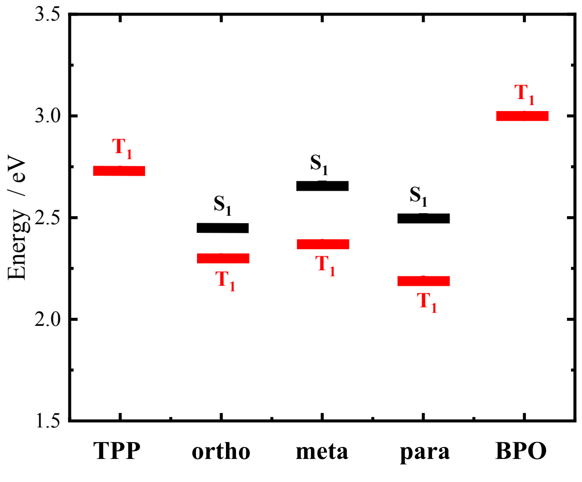

Figure S29. Sketch of the singlet and triplet excited-state energies of **TPP**, **BPO**, **o-PTZ**, **m-PTZ**, and **p-PTZ** as obtained from the emission measurements at 77 K (Table S9).

Bioimaging and Cellular Phototoxicity

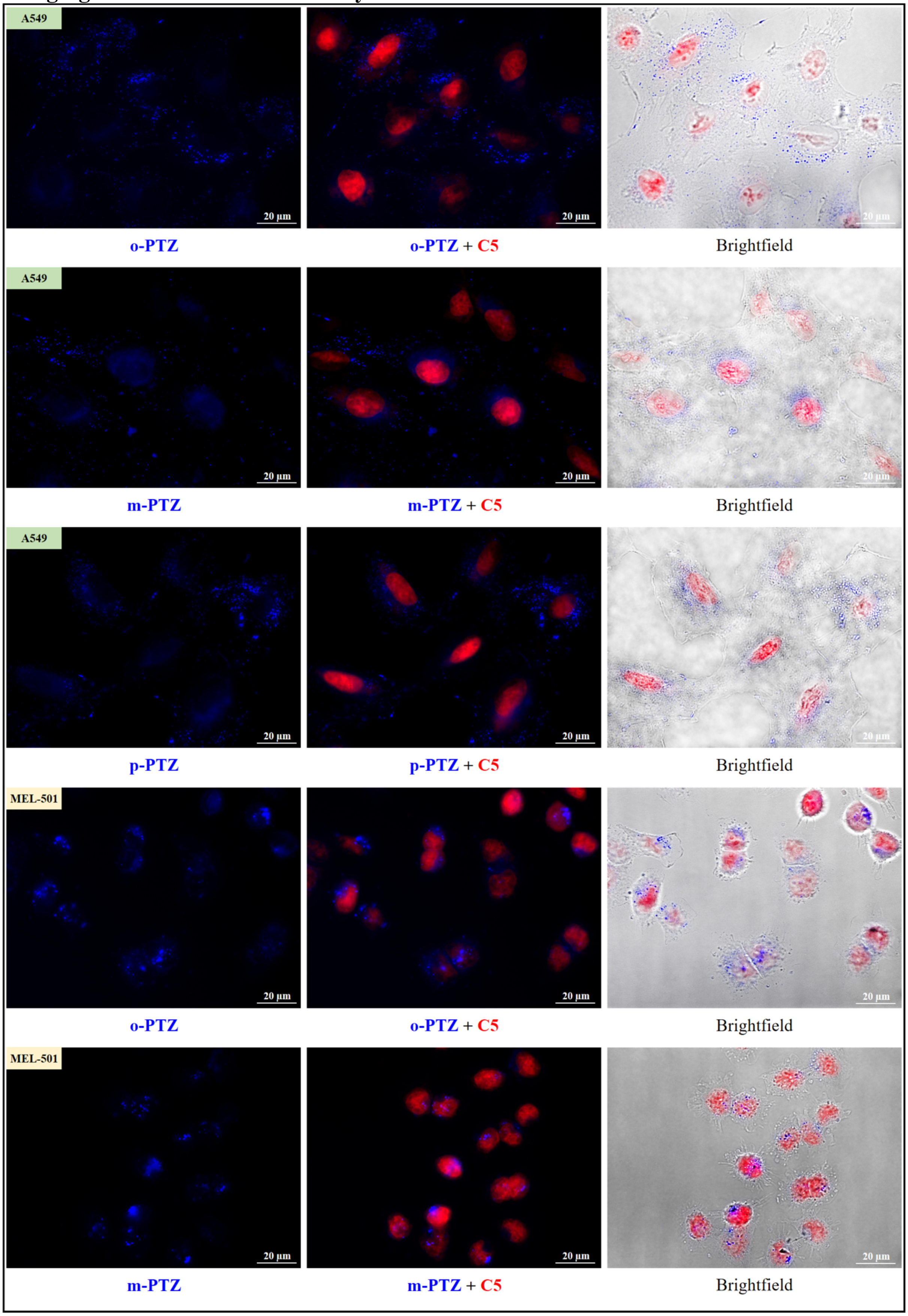

Figure S30. Representative fluorescence microscopy images of the isomers (blue, DAPI filter), the isomers and the nuclei marker **C5** (red, TRITC filter), and relative brightfield merged images of A549 and MEL-501 cells (Image magnification: 60×).

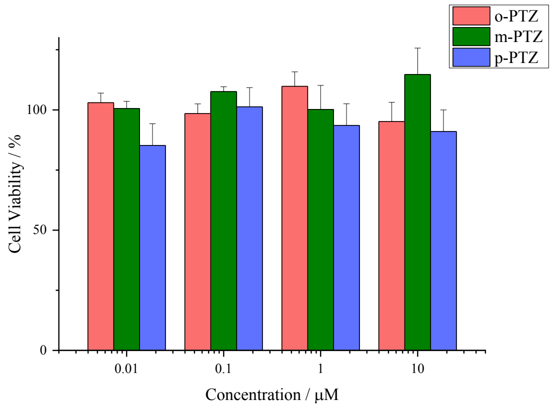

Figure S31. Isomer effect on MEL-501 cell growth, expressed as mean of two independent experiments of four replicas each ± SD. 100% cell growth corresponds to control mean values.

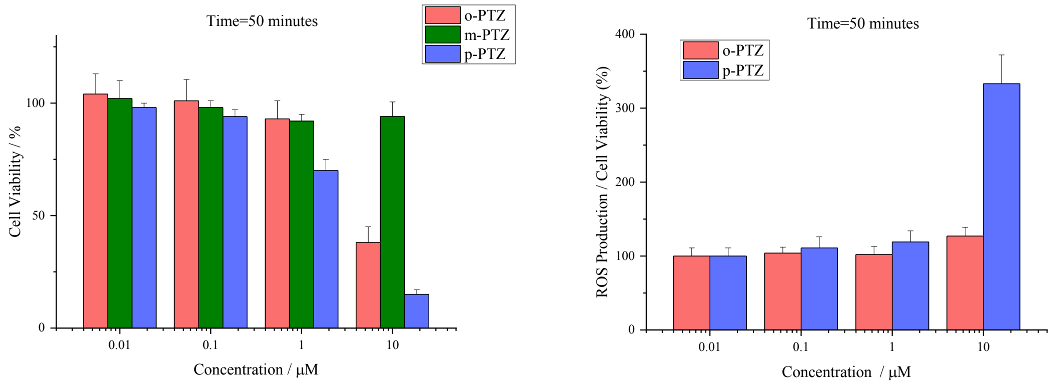

Figure S32. Phototoxicity (left) and ROS production (right) of different concentrations of the isomers on MEL-501 cells with 50 minutes irradiation time (corresponding to an irradiation energy of 5.10 J/cm^2), expressed as mean of two independent experiments of four replicas each ± SD. 100% corresponds to control mean values.

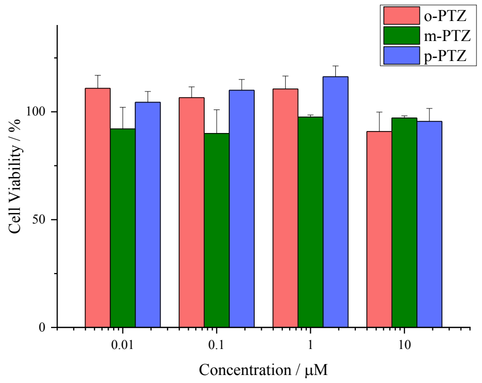

Figure S33. Isomer effect on A549 cell growth, expressed as mean of two independent experiments of four replicas each ± SD. 100% cell growth corresponds to control mean values.

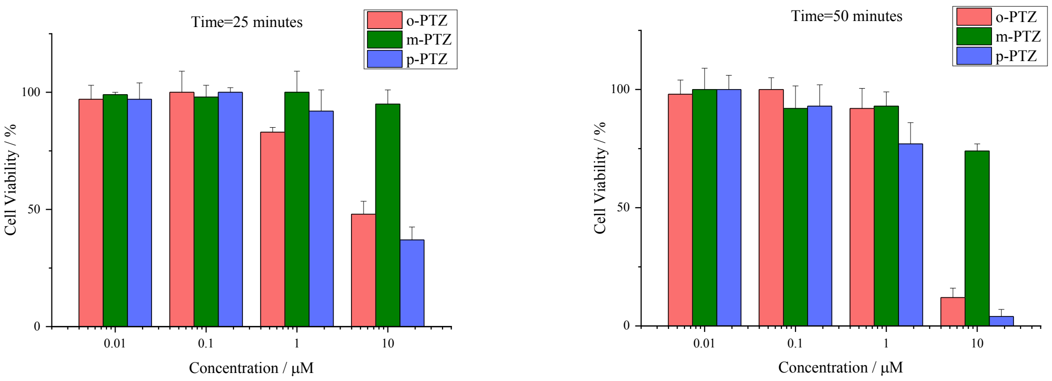

Figure S34. Phototoxicity of different concentrations of the isomers on A549 cells with 25 (left) and 50 (right) minutes of irradiation time (corresponding to irradiation energies of 2.55 and 5.10 J/cm^2, respectively), expressed as mean of two independent experiments of four replicas each ± SD. 100% cell growth corresponds to control mean values.

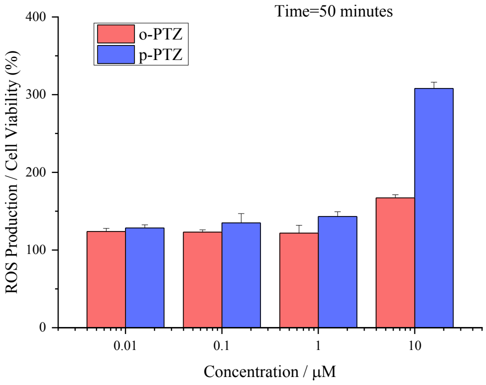

Figure S35. ROS production of different concentrations of the isomers on A549 cells with 50 minutes of irradiation time, expressed as mean of two independent experiments of four replicas each ± SD. 100% corresponds to control mean values.

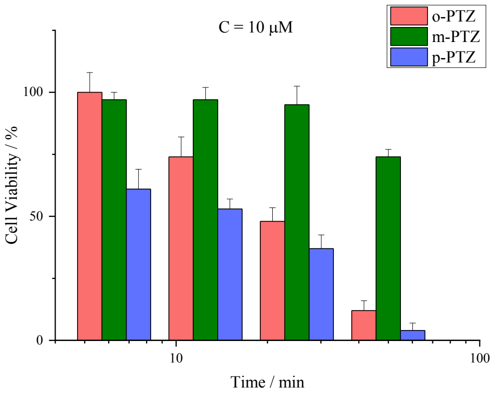

Figure S36. Phototoxicity of 10 µM solutions of the isomers on A549 cells at different irradiation times of 6, 12.5, 25, and 50 minutes, expressed as mean of two independent experiments of four replicas each ± SD. 100% cell growth corresponds to control mean values.

Table S12. Phototoxicity of the isomers on MEL-501 and A549 cells at different irradiation times, expressed as IC50 values.

	Irradiation Time	IC50		
		**o-PTZ**	**m-PTZ**	**p-PTZ**
MEL-501	25’<br>50’	> 10 μM<br>6.6±0.8 μM	-<br>-	3±1 μM<br>2.3±0.1 μM
A549	25’<br>50’	9±2 μM<br>5.7±0.2 μM	-<br>> 10 μM	6±1 μM<br>3±1 μM

Characterization

^{1}H and ^{13}C NMR spectra of the isomers:

***o*-PTZ** Yield: 79%; ^{1}H NMR (400 MHz, $CDCl_3$, 25 ºC): δ 8.05–8.07 (m, 2H), 7.75 (d, J = 8.0 Hz, 1H), 7.43–7.52 (m, 3H), 7.30–7.38 (m, 2H), 7.10–7.16 (m, 3H),6.99 (d, J = 8.0 Hz, 1H), 6.91 (t, J = 8.0 Hz, 1H), 6.85 (d, J = 8.0 Hz, 1H), 6.73 (d, J = 8.0 Hz, 1H), 3.79 (t, J =8.0 Hz, 2H), 1.76–1.88 (m, 2H), 1.01 (t, J =8.0 Hz, 3H); ^{13}C NMR (100 MHz, $CDCl_3$, 25 ºC): δ 167.7, 152.8, 144.9, 144.9, 140.6, 136.7, 134.1, 132.5, 130.8, 130.4, 130.0, 129.2, 128.3, 127.6, 127.4, 127.2, 125.8, 124.8, 124.7, 124.3, 123.2, 122.4, 121.4, 115.3, 114.9, 49.3, 20.0, 11.3, 0.0 ppm.

***m*-PTZ** Yield: 71%; ^{1}H NMR (400 MHz, $CDCl_3$, 25 ºC): δ 8.27 (s,1H), 8.11 (d, J = 8.0 Hz, 1H), 8.02 (d, J = 8.0 Hz, 1H), 7.93 (d, J = 8.0 Hz, 1H), 7.64–7.66(m, 1H), 7.46–7.55 (m, 4H), 7.39–7.43 (m, 1H), 7.15–7.18 (m, 2H), 6.88–6.95 (m, 3H), 3.87 (t, J =8.0 Hz, 2H), 1.83–1.92(m, 2H), 1.04 (t, J =8.0 Hz, 3H); ^{13}C NMR (100 MHz, $CDCl_3$, 25 ºC): δ 168.0, 154.1, 154.1, 144.9, 140.9, 135.1, 134.3, 134.1, 129.4, 129.0, 127.4, 127.3, 126.3, 126.1, 126.0, 125.8, 125.4, 125.4, 125.2, 124.4, 123.2, 122.5, 121.6, 115.5, 115.4, 49.2, 20.1, 11.3, 0.0 ppm.

***p*-PTZ** Yield: 73%; ^{1}H NMR (400 MHz, $CDCl_3$, 25 ºC): δ 8.13 (d, J = 8.0 Hz, 2H), 8.08 (d, J = 8.0 Hz, 1H), 7.92 (d, J = 8.0 Hz, 1H), 7.66 (d, J = 8.0 Hz, 2H), 7.50 (t, J = 8.0 Hz, 1H), 7.37–7.46 (m, 3H), 7.15 (d, J = 8.0 Hz, 2H), 6.87–6.94 (m, 3H), 3.86 (t, J =8.0 Hz, 2H), 1.83–1.91(m,2H), 1.04 (t, J =8.0 Hz, 3H); ^{13}C NMR (100 MHz, $CDCl_3$, 25 ºC): δ 167.7, 154.2, 145.1, 144.8, 142.4, 135.3, 135.0, 134.0, 132.1, 128.9, 128.0, 127.5, 127.3, 126.8, 126.3, 125.9, 125.7, 125.4, 125.1, 124.3, 123.1, 122.5, 121.6, 115.5, 115.4, 49.2, 20.1, 11.3,0.0 ppm.

Copies of NMR spectra

^{1}H NMR of **o-PTZ**:

RM-AE-OPTZ.001.esp

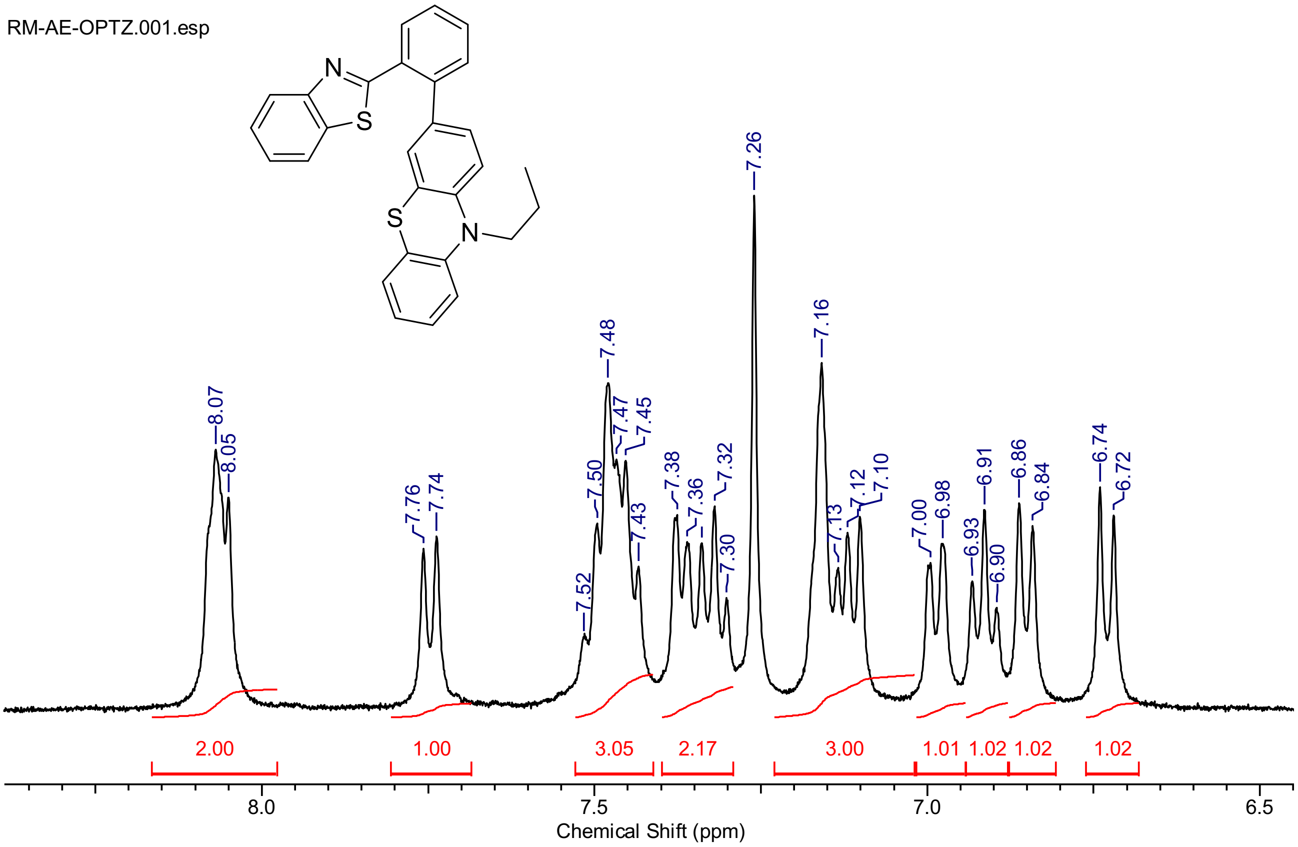

RM-AE-OPTZ.001.esp

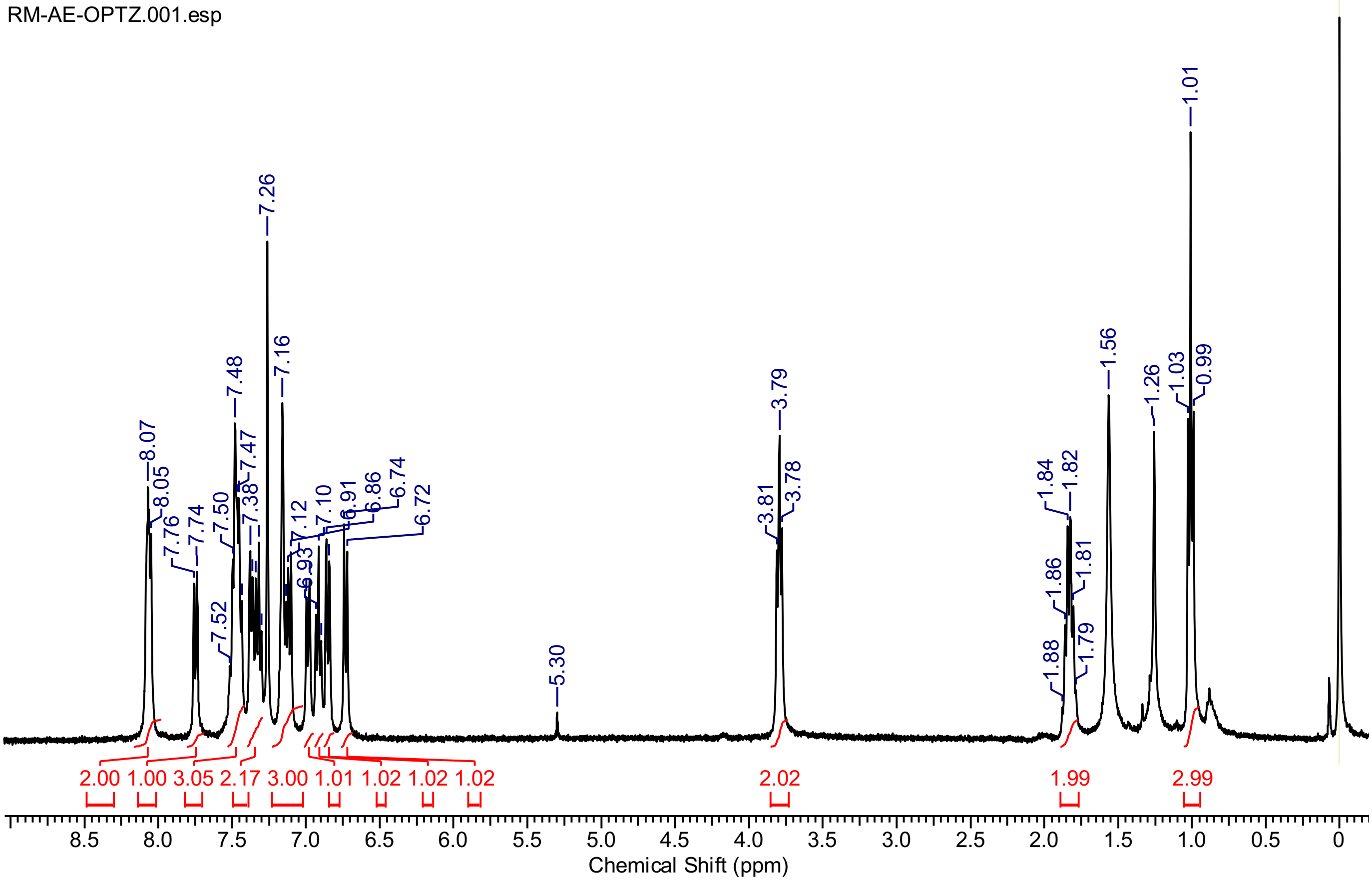

^{13}C NMR of ***o*-PTZ**:

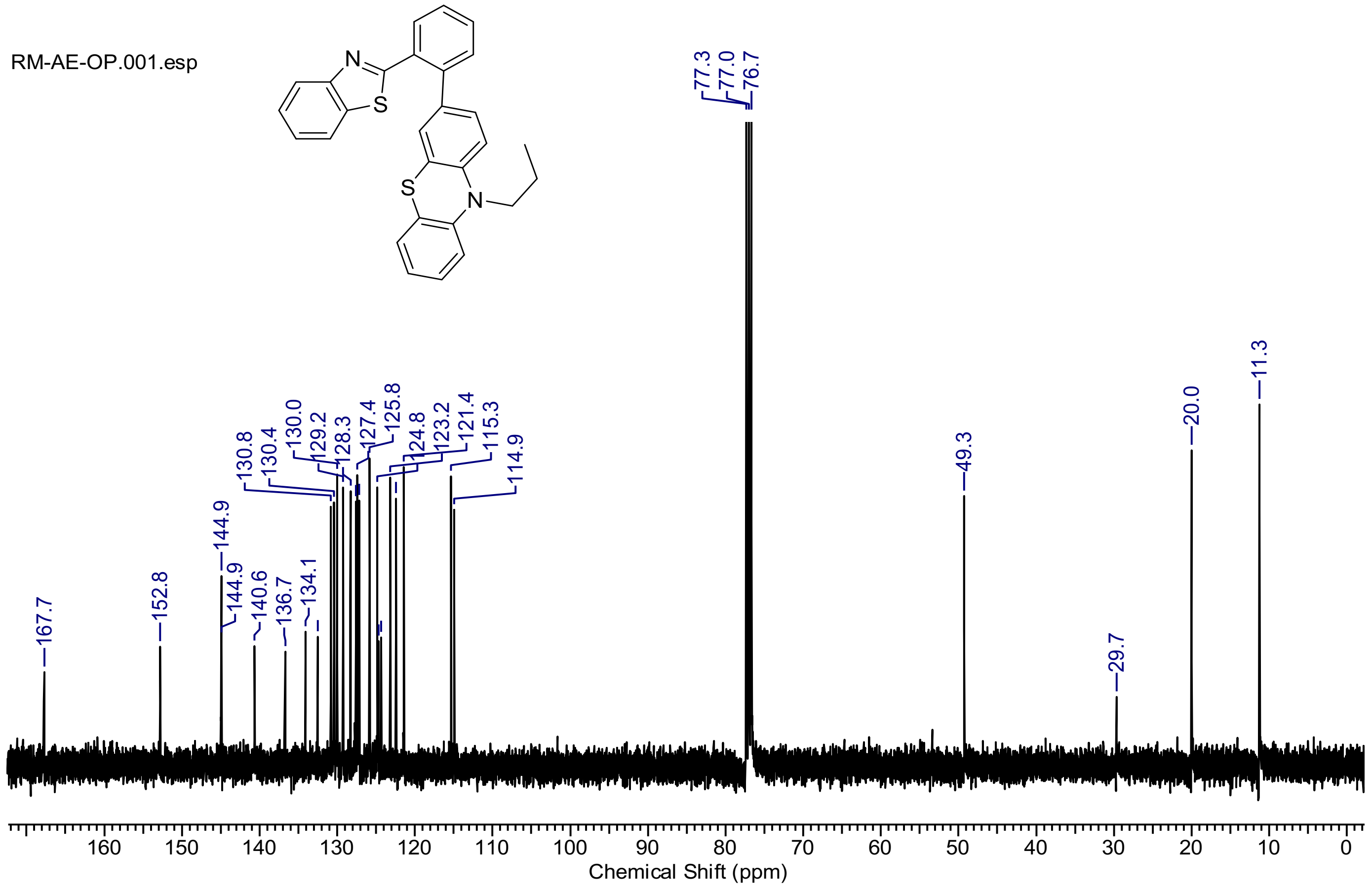

^{1}H NMR of ***m*-PTZ**:

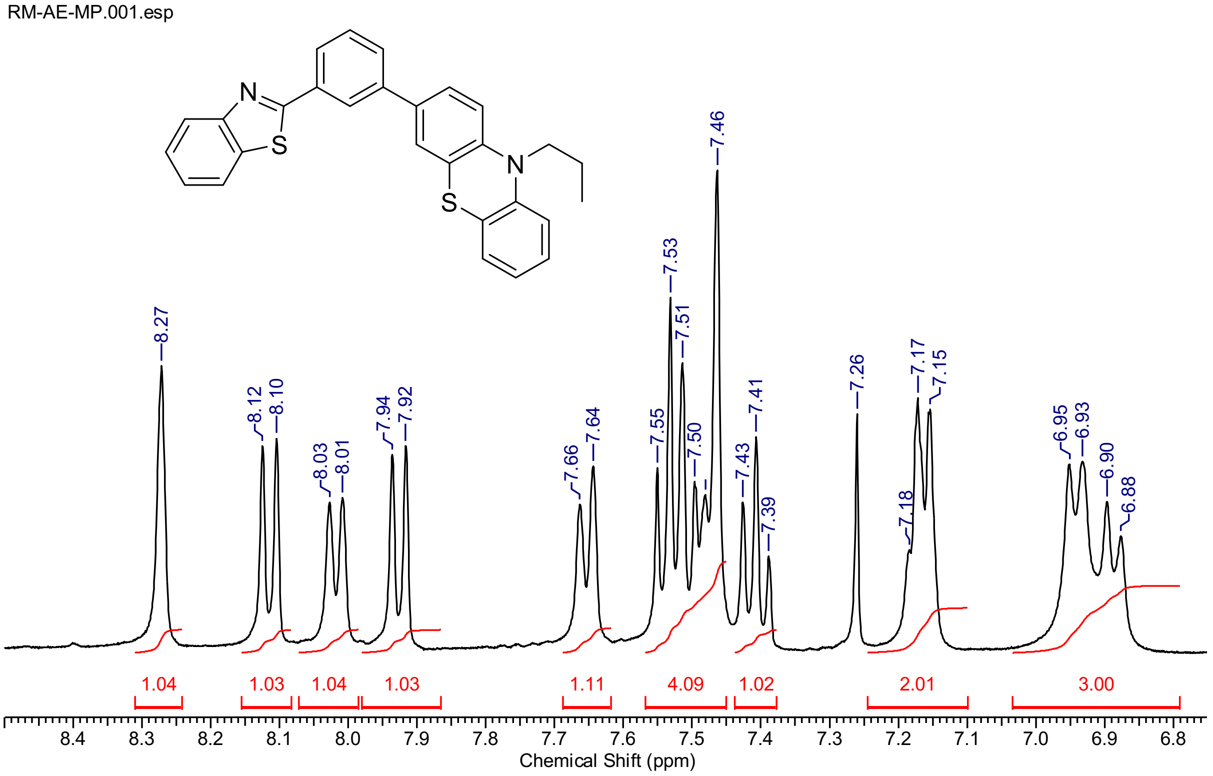

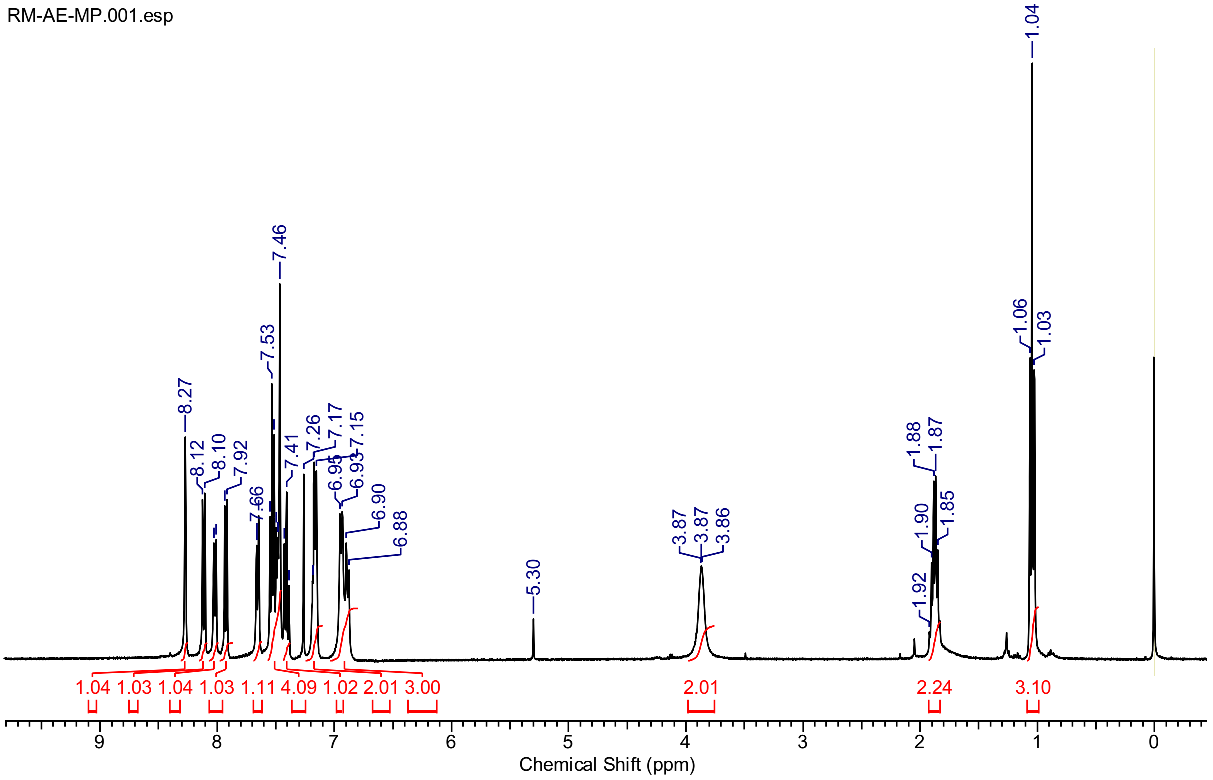

^{13}C NMR of **m-PTZ**:

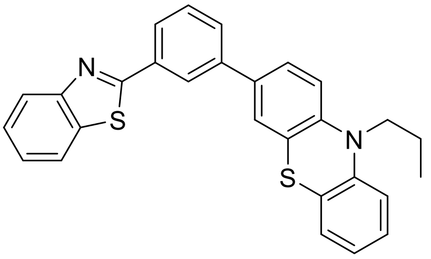

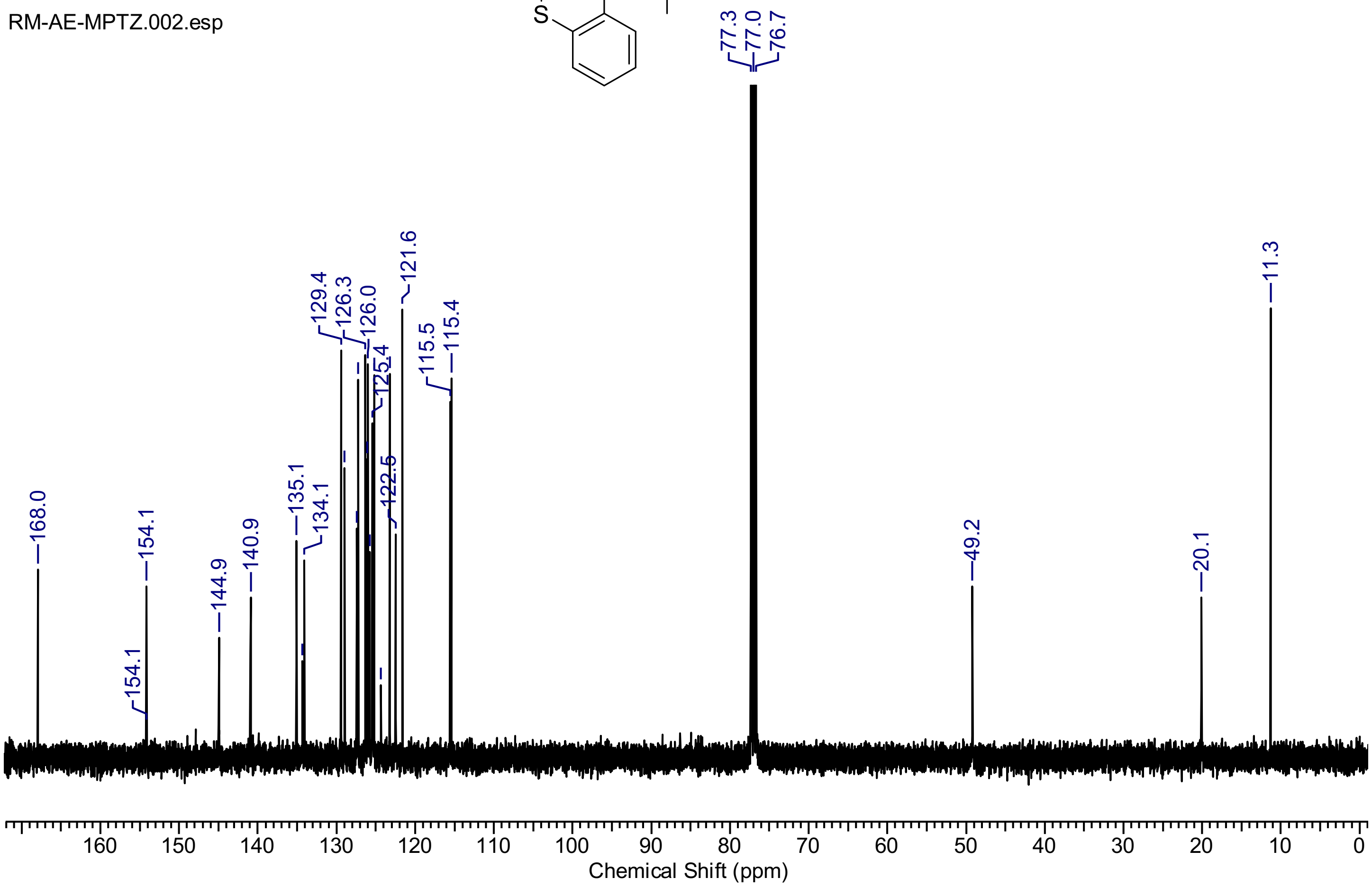

^{1}H NMR of **p-PTZ**:

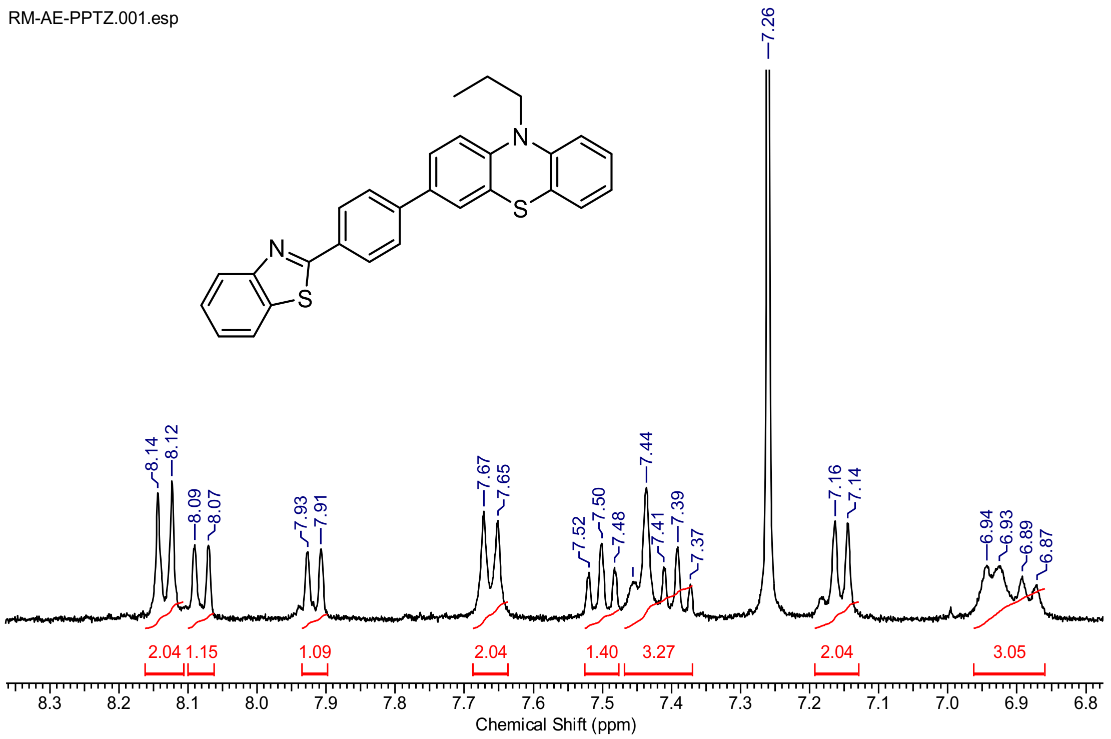

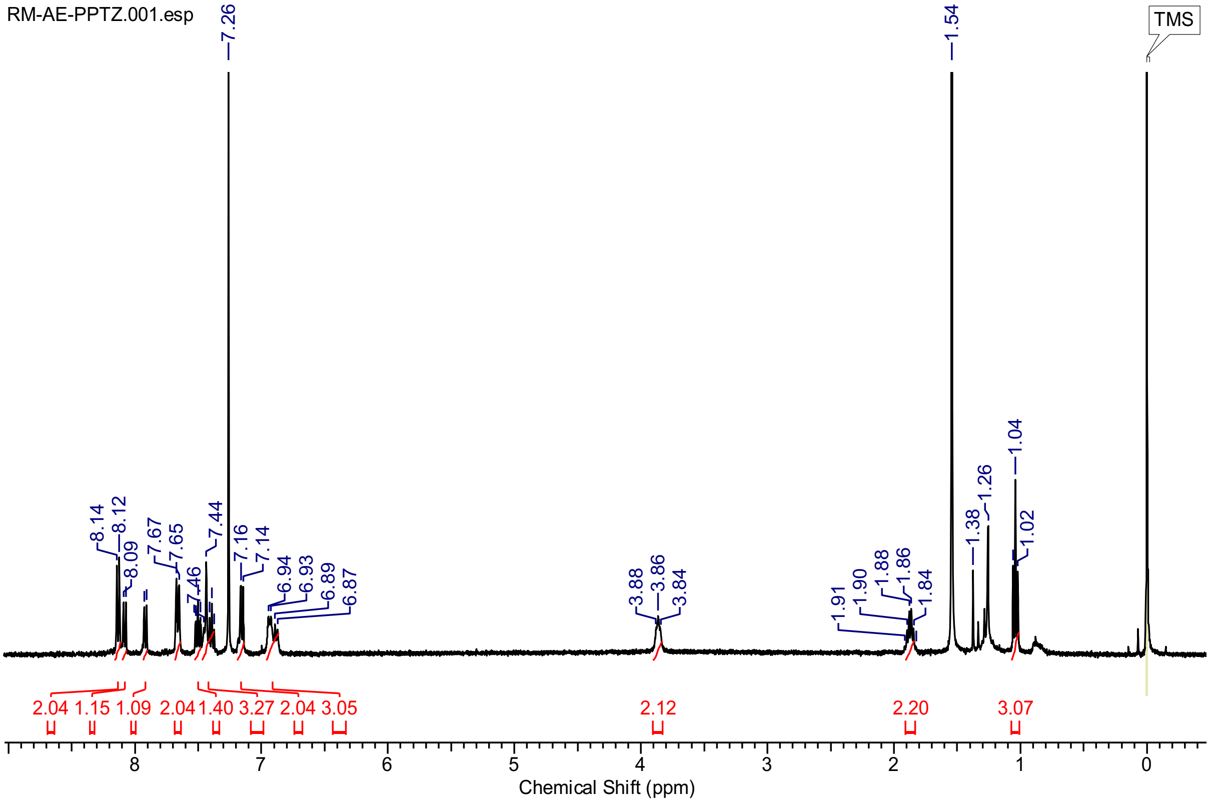

^{13}C NMR of **p-PTZ**:

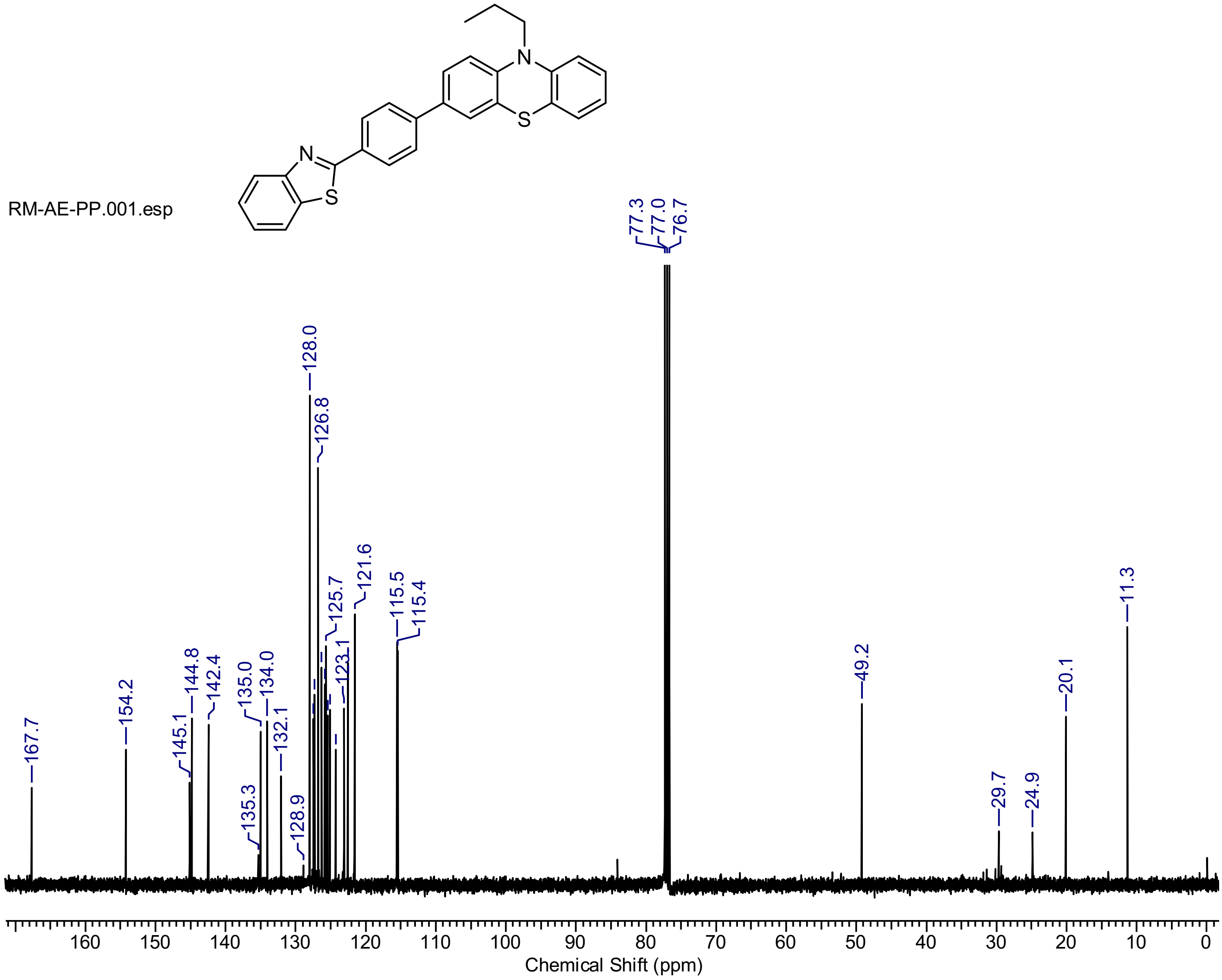

HPLC and % purity of the compounds

***o*-PTZ**

Retention time: 20.308 min

% Purity: 90.36 %

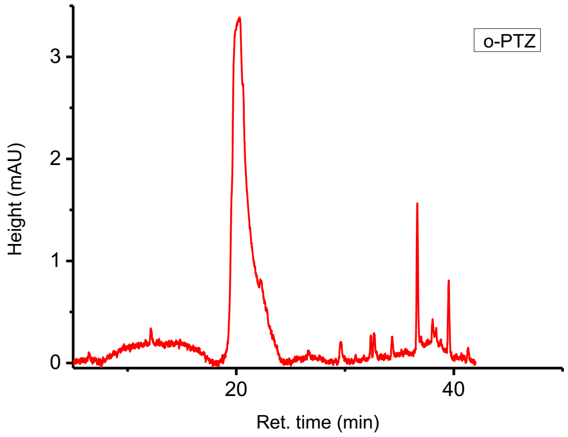

***m*-PTZ**

Retention time: 20.317 min

% Purity: 95.59 %

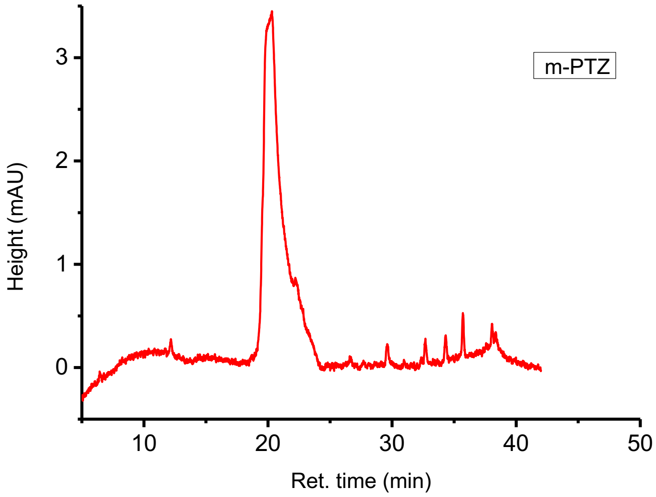

***p*-PTZ**

Retention time: 35.033 min

% Purity: 91.06 %

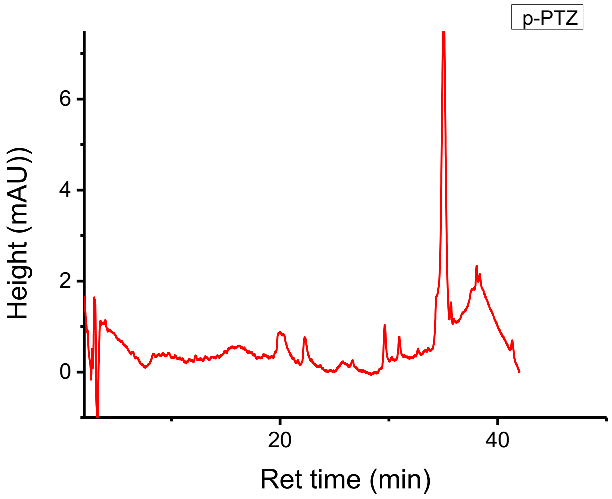

Supplement: Supplementary file 1 — jp2c07717_si_001.pdf [file jp2c07717_si_001.pdf]
